# Projecting tick-borne encephalitis risk in Sweden under climate change scenarios: a high-resolution spatio-temporal modeling approach

**DOI:** 10.1186/s12940-026-01278-8

**Published:** 2026-03-23

**Authors:** Maquins Odhiambo Sewe, Jonas Wallin, Joacim Rocklöv, Shiyu Wang, Torben Koenigk, Jan C. Semenza

**Affiliations:** 1 Department of Epidemiology and Global Health, Norrland University Hospital, Umeå University, 23, Destination F52,Elsa Bolins Gränd 6, 5th floor, Umeå, 90185 Sweden; 2 Heidelberg Institute of Global Health, Heidelberg University, Heidelberg, Germany; 3 Department of Statistics, Lund university, Lund, Sweden; 4 Interdisciplinary Centre for Scientific Computing, Heidelberg University, Heidelberg, Germany; 5 Swedish Meteorological and Hydrological Institute, Norrköping, Sweden

## Abstract

**Background:**

Tick-borne encephalitis (TBE) is a serious vector-borne neurological disease in Europe, with a complex transmission cycle involving ticks of genus *Ixodes*, animal hosts, environmental and climatic determinants.

**Methods:**

We modelled annual Geocoded Swedish TBE case data for the period 2005–2023 as a log-Gaussian Cox process in relation to population, environmental and climate data, and wildlife citizen science reports at high spatial resolution. We used the computationally efficient Integrated Nested Laplace Approximation (INLA) and projected the future TBE incidence using fifteen regional climate models.

**Results:**

The covariates significantly associated with TBE incidence in Sweden, ranked based on predictive capacity, were mean temperature, population density, habitat richness, forest cover, precipitation, relative humidity and roe deer density. Specifically, mean temperature above 12° C degrees in the third quarter of the previous year, habitat richness, precipitation in the third quarter, and higher roe deer density were associated with increased TBE risk. The model performed well on testing data, excluded from model building, demonstrating high predictive accuracy in TBE-endemic areas compared to observed data. Our projections indicate TBE cases will increase by 69% under low emissions (RCP2.6) and 121% under high emissions (RCP8.5) by the 2090s, relative to 2014–2023.

**Conclusion:**

The TBE incidence is projected to rise substantially, even under lower emission scenarios. Our findings highlight the growing influence of climate change on TBE transmission in Sweden and provide actionable evidence to inform surveillance, vaccination strategies, and long-term public health planning. Citizen science initiatives and risk maps can help focus resources on areas most vulnerable to transmission. More broadly, the integration of climate models with high-resolution epidemiological data, offers a template for anticipating climate-sensitive vector-borne diseases. Proactive, evidence-based interventions are essential to mitigate the growing health burden posed by TBE in Sweden and beyond.

**Supplementary Information:**

The online version contains supplementary material available at 10.1186/s12940-026-01278-8.

## Introduction

Tick-borne encephalitis (TBE) is a viral infection of the central nervous system that is endemic across large areas ranging from northern China and Japan to eastern Russia and Europe, including the eastern, central, northern, and, increasingly, western parts of the continent [1]. The disease is caused by the tick-borne encephalitis virus (TBEV), a member of the *Flaviviridae* family, and is primarily transmitted through the bite of infected ticks [2]. Several viral subtypes of TBEV have been identified, but in Europe the predominant strain is TBEV-Eu, for which the principal vector is the hard tick *Ixodes ricinus* (*I. ricinus*) [3, 4]. The risk of infection rises in endemic areas through exposure to infected ticks, often during outdoor recreational or occupational pursuits such as hunting, fishing, camping, forestry, farming, military training, or foraging for mushrooms and berries [5]. Transmission can also occur via the consumption of unpasteurized contaminated milk [6].

TBEV is maintained in nature by a range of vertebrate hosts with transient viremia and is transmitted mainly via bites from infected *Ixodes* ticks, the primary vector [7]. Small mammals, particularly mice and voles, serve as competent reservoir hosts, capable of sustaining viremia sufficient to infect feeding ticks [8, 9]. These ticks, once infected, perpetuate transmission by subsequently feeding on other susceptible mammalian hosts. By contrast, larger mammals such as roe deer typically exhibit only minimal or undetectable viremia and are thus unable to effectively propagate the virus; they are instead regarded as incidental hosts or incompetent reservoir [5]. High levels of viremia sufficient to enable transmission occur only in mammals such as sheep, goats, horses, dogs, and rodents. Humans do not participate in the transmission cycle and are considered incidental and dead-end hosts [5]. In humans, TBE may result in neurological illness, though a considerable proportion of infected individuals remain asymptomatic [10]. The incubation period is typically seven days [10]. Initial symptoms often resemble a nonspecific viral illness, presenting with fever, fatigue, headache, myalgia, and nausea, which usually abate after several days [1, 10]. However, in some cases, a second phase develops within 1 to 33 days, marked by neurological involvement such as meningitis and/or encephalitis [2, 10]. As no specific antiviral therapy for TBEV exists, management is limited to supportive and symptomatic care. Fortunately, an effective vaccine is available and remains the cornerstone of prevention [11].

Within the European Union (EU), TBE is classified as a notifiable disease. In 2022, 22 EU/EEA countries reported 3,516 TBE cases that met the EU case definition [12]. This corresponds to a notification rate of 0.81 per 100 000 population, which is a 14% increase compared to the results from 2021. Sweden, in particular, has experienced a striking upward trend over the past three decades, with a notification rate reaching 3.8 cases per 100,000 population between 2017 and 2021, one of the highest national rates in Europe [13]. Notably, the overwhelming majority of confirmed cases (95%) occurred in unvaccinated individuals, among whom the notification rate reached as high as 12 cases per 100,000 population [14].

Mean air temperature has increased at an unprecedented rate in northern Europe in recent decades, especially after the year 1990 [15]. In northern Europe, the tick development rates of every life cycle process examined have accelerated, including oviposition, incubation rates and molting rates, affecting the development processes of the tick *I. ricinus* [5, 16]. This could result in an increased likelihood of infected ticks transmitting the TBE virus to reservoir hosts as well as humans. Rising temperatures and altered precipitation patterns, can also affect the distribution and abundance of *Ixodes* vector populations [5, 17]. Warmer temperatures and milder winters can expand the geographical range of ticks, allowing them to establish populations in regions previously unsuitable for their survival [5, 18]. This expansion may result in an increased risk of TBE transmission in areas where the disease was previously less common or absent [18, 19].

In Europe, *I. ricinus* ticks, have expanded to northern latitudes and to higher altitudes and are now present virtually throughout the entire country of Sweden [19–21], indicating that climate may be a driving force in TBE transmission [17, 22]. In fact, in Sweden, where TBE is endemic, the increased TBE prevalence has been linked to two consecutive years of less winter cold, earlier springs and later autumns, with elevated mean temperatures [23]. A spatiotemporal range expansion of *I. ricinus* occurred on a greater scale in Sweden, owing to warmer winters and prolonged spring and autumn periods with minimum temperatures ranging between 5 and 8°C [20, 21]. Thus, warmer temperatures can extend the tick activity season, increasing the duration of time during which people may be exposed to tick bites [18, 24]. Additionally, ticks may become active earlier in the spring and remain active later in the autumn, lengthening the tick questing season [18, 24]. Elevated temperatures also increase the number of susceptible ticks (in their larval and nymphal stages) and the number of infected nymphal ticks that co-feed on the same hosts [25]. If an uninfected tick feeds near an infected tick on an animal host, the naïve tick can become infected. During this co-feeding process, the animal host serves as a bridge for transmission of TBEV to naïve ticks [25].

Despite efforts made in quantifying TBE cases that are attributed to climate change, several other significant drivers account for morbidity patterns [26]. To elucidate the true incidence of TBE in Sweden, data on the interplay between vertebrate hosts and climatic and environmental variables are needed when developing predictive models. To date, there is no modelling study that has factored in these complex interactions. This study relies on high-resolution Swedish TBE data to build a robust spatiotemporal predictive model, where the (likely) location of exposure was geocoded and time stamped. These data were linked to quarterly climate data, vegetation greenness such as the normalized difference vegetation index (NDVI) and forest cover, leaf area index (LAI), wildlife data, such as roe deer density and human population data. We use the developed model to compute both short-term predictions and long-term projections of climate change impacts on TBE incidence in Sweden considering different greenhouse gas emission scenarios (Representative Concentration Pathways, or RCPs) from regional climate models (RCMs). These projections will serve as the backbone for estimating the European-wide risk of TBE under climate change scenarios and to target vaccination, surveillance, and early detection of new TBE foci.

## Methods

### Data

We used TBE data reported by the public health agency of Sweden for the years 2005 to 2023. To model the risk of TBE, we included satellite-derived climatic factors, land cover, population density, elevation, roe deer density, and habitat richness (a spatially explicit aspect of biodiversity [27]), as described and listed in the Supplementary material (Table S1: Data sources). We have also included a table describing the reasons for including the predictors in the model, see table S2 (Variable inclusion reason). High-resolution (100 m) maps of roe deer density were obtained from a web-based citizen science platform [28]. The habitat types data at 10 km x 10 km grid were downloaded from the EEA website [29].

To project the impact of climate change on TBE incidence in Sweden, we included climate data from future projections with RCMs considering three greenhouse gas emission scenarios, the optimistic, low emission scenario RCP26, the intermediary RCP45 and the extreme, high-emission scenario RCP85 for the period 2024–2098 provided by the Swedish Meteorological and Hydrological Institute (SMHI) https://www.smhi.se/ (Table S3: Projections data sources).

### Quarterly indicators

For each of the environmental covariates, min temperature, mean temperature, max temperature, precipitation, relative humidity, NDVI, LAI, and forest cover we computed the annual quarterly indicators, (Table S4: Derived quarterly indicators). For each indicator, we also computed the previous year’s value in the same quarter, labelled as LAG1.

### Statistical modelling

The annual TBE point data were modeled as a log-Gaussian Cox process, a hierarchical Poisson process with random intensity assuming a latent Gaussian random field. The covariance structure of the Gaussian latent random field can be estimated using the computationally efficient integrated nested Laplace approximation (INLA) approach introduced by Rue et al. [30] within a Bayesian framework in the R environment [31]. Specifically, we used INLA_25.10.19 and R 4.5.2 [32] version for the analysis. We used the approach by Simpson et al. [33] that utilizes a finite-dimensional continuously indexed random field (mesh) estimated using stochastic partial differential equations (spde) introduced by Lindgren et al. [34] to model the latent Gaussian random field. We created a mesh covering the whole of Sweden. We describe the modelling in detail in Supplementary materials; briefly.

### Constructing of the mesh

To define the Gaussian random field, we constructed a triangulated mesh covering the whole of Sweden as described and shown in Figure S3 using mesh creation functions within R-INLA [31]. Three parameters are crucial to generate a good mesh. These are the max edge, offset and cutoff values. The max edge determines the resolution of the mesh, the offset value controls how far the domain can be extended to adjust for boundary effects while the cutoff value is used to avoid having several vertices within clustered data points. For the TBE model, we chose a maximum edge of 42 km for the inner domain, 210 km for the outer domain and a cutoff value of 9 km. This resulted in 1379 mesh locations. Sensitivity analysis following suggestions by [35] was used in the selection of the best mesh configuration.

### Model selection

The first level of the hierarchical structure is the intensity of TBE cases in each polygon captured by a Poisson likelihood. The second level involves selection of the linear predictor part of the model as detailed below. First, we included each covariate (the environmental indicators) one by one in the base model which included the intercept, spatial random effect, elevation, the year random effect and log population density. We included population as a fixed effect as opposed to an offset. By including log(population) as an estimated fixed effect, rather than strictly as an offset, we allow the model to distinguish between its role as a denominator (incidence per person) and its direct ecological impact on tick populations. For each class of covariate, we selected the best one based on DIC, as shown in Table S16 (Variable selection). We then tested for multicollinearity on the remaining variables based on the variance inflation factor (VIF) estimated with the R package usdm [36]. Using a threshold of 2.5, NDVI and elevation were dropped due to high collinearity, see Table S17 (Multi collinearity test). The selected indicators were then included in a joint model that made up the final model. We tested the influence of each selected predictor on the model’s predictive capacity by iteratively removing each one and computing the effect on the overall model Deviance Information Criterion (DIC) value. The predictors were then ranked based on contribution to the overall predictive capacity.

### Model validation

Based on the selected model above, we evaluated the models’ predictive accuracy by doing out-of-sample predictions for the years 2020 to 2023. Iteratively, we made predictions on a regular grid (5 km X 5 km) for each year in the validation set based on data for the preceding year’s excluding the prediction year (see Supplementary material). As a comparative reference model, we built a negative binomial model of the annual TBE cases aggregated at the municipality level. The reference model included previous TBE cases and a smoothed population covariate, following the approach used in building TBE predictive models in Poland [37].

### Climate impact projections

The final selected model was used to project the potential impact of climate change on TBE incidence. We present the percentage change in mean TBE incidence relative to the baseline years (2014–2023) as a measure of impact. We aggregated and compared the results at a generated regional grouping, as described in Supplementary material and Figure S4.

## Results

**Fig. 1:**
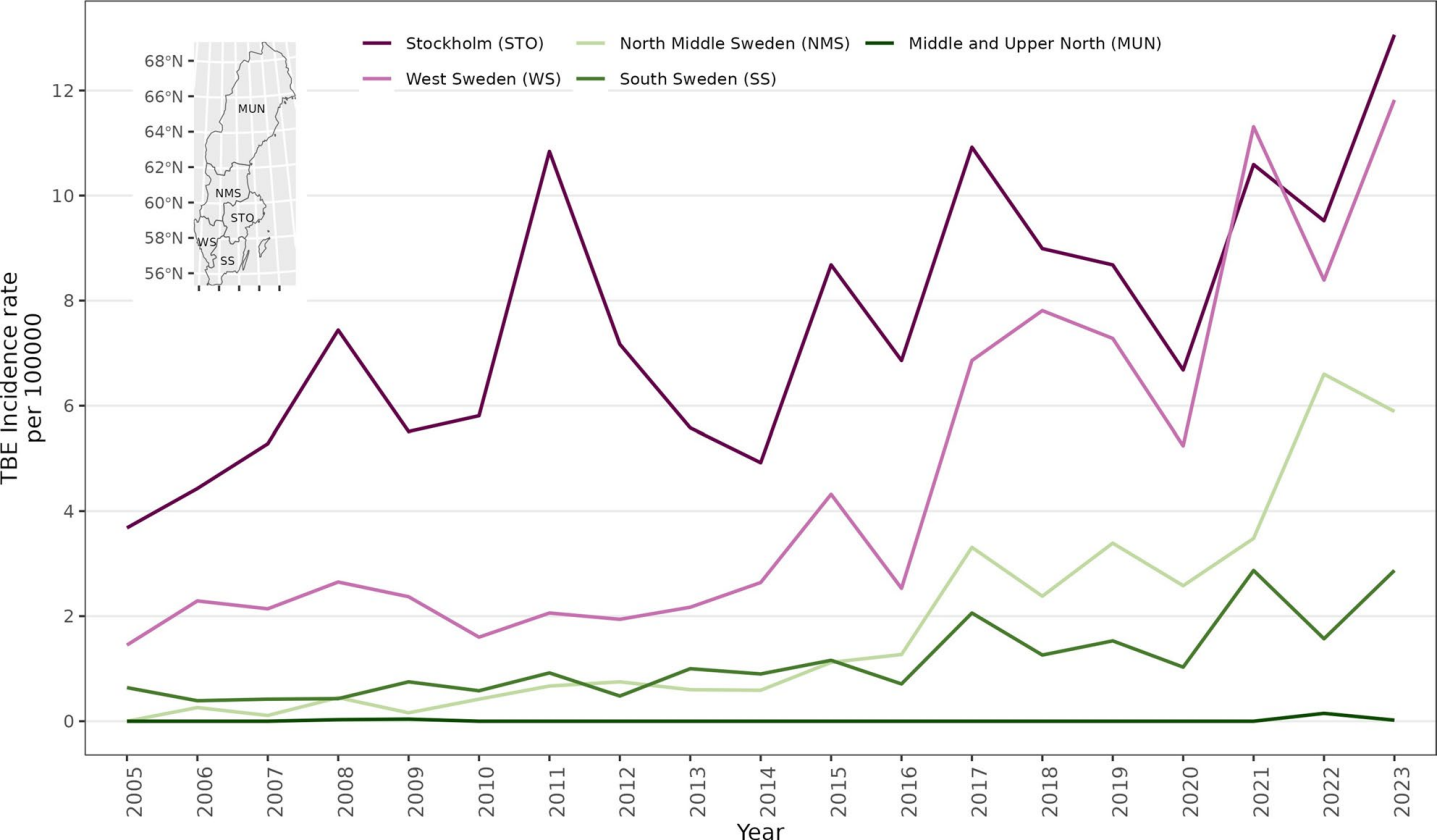
Trends in TBE incidence rates by region, Sweden, 2005–2023

Between 2005 and 2023, there were 4,880 TBE cases reported in Sweden. Figure 1 shows trends in TBE incidence rates by region in Sweden, while Figure S21(a) shows the spatial distribution of TBE cases in Sweden, with most of the cases occurring in the southern part of the country. The Stockholm area is taking the biggest brunt of the TBE disease burden, accounting for about 70% of the reported TBE cases. Summaries for the whole of Sweden are shown in Table 1, while the regional summaries are shown in Tables S5-S9. We observe a steady year-on-year increase in TBE cases over the period. In the period 2021–2023, a total of 1,390 TBE cases (4.4 per 100,000) were reported in Sweden, compared to 414 (1.51 per 100,000) in the period 2005–2007 corresponding to a 70% increase in the reported cases (Table 1). Similarly, we observe an increase of about 60% in cases in the period 2012–2014. During the study period, the population in Sweden increased by 13% from nine million to over ten million. Overall, it appears that the roe deer density has remained stable over time in the whole of Sweden, though we had only four data points during the study period. However, regionally (Supp Tables 5, 6, 7, 8 and 9) the southern part of the country registered the highest density. Similarly, the highest habitat richness (0.61) was observed in the southern part of Sweden (Table S8), while the lowest 0.49 was in northern Sweden (Table S9).

**Table 1:** Descriptive summary of TBE incidence rates per 100,000 population, and covariates by quarter and period, Sweden 2005–2023

	Quarter	Year					
		2005–2007	2008–2011	2012–2014	2015–2017	2018–2020	2021–2023
TBE incidence rate		1.71	2.63	2.3	3.83	4.18	6.33
Population (millions)		9.11	9.37	9.65	9.99	10.31	10.51
Elevation (m)		204.65	204.65	204.65	204.65	204.65	204.65
Roe deer density		0.13	0.13	0.12	0.13	0.14	0.14
Habitat richness		0.56	0.56	0.56	0.56	0.56	0.56
Mean temperature (°C)	Q01	-3.19	-3.65	-2.53	-1.58	-1.82	-1.79
	Q02	9.21	9.52	8.94	8.83	10.23	9.56
	Q03	14.41	13.93	13.97	13.76	14.44	14.29
	Q04	2.56	0.58	1.8	2.18	2.66	0.91
Minimum temperature (°C)	Q01	-6.92	-7.05	-5.9	-4.84	-5.17	-5.43
	Q02	4.01	4.04	3.92	3.92	4.39	3.89
	Q03	9.72	9.73	9.2	9.35	9.71	9.91
	Q04	-0.34	-2.33	-1	-0.7	0.09	-1.91
Maximum temperature (°C)	Q01	0.51	-0.33	0.85	1.74	1.48	1.91
	Q02	14.41	14.88	13.99	13.83	15.88	15.02
	Q03	19.4	18.57	19	18.54	19.49	19.05
	Q04	5.53	3.45	4.5	5.05	5.24	3.69
Precipitation (mm)	Q01	130.56	129.19	113.5	131.07	157.53	144.59
	Q02	158.17	142	179.94	153.1	117.59	124.29
	Q03	225.1	273.66	210.73	218.95	197.69	262.73
	Q04	200.48	188.87	212.3	179.72	201.47	181.04
Relative Humidity (%)	Q01	83.23	84.34	83.19	85.11	82.66	80.87
	Q02	73.03	72.51	73.9	72.8	67.09	67.36
	Q03	80.23	81.97	80.69	80.77	76.86	78.78
	Q04	88.26	88.03	88.93	87.64	88.15	86.9
NDVI	Q01	0.08	0.08	0.09	0.15	0.17	0.12
	Q02	0.44	0.45	0.46	0.46	0.48	0.51
	Q03	0.6	0.59	0.61	0.59	0.61	0.7
	Q04	0.31	0.29	0.22	0.31	0.26	0.29
Forest cover (%)	Q01	0.22	0.22	0.23	0.23	0.22	0.1
	Q02	0.48	0.49	0.5	0.49	0.49	0.42
	Q03	0.57	0.57	0.58	0.59	0.53	0.47
	Q04	0.22	0.23	0.23	0.23	0.18	0.09
Leaf area index	Q01	0.76	0.76	0.81	0.86	0.85	0.35
	Q02	1.99	2.05	2.13	2.12	1.98	1.81
	Q03	2.48	2.49	2.57	2.72	2.35	2.27
	Q04	0.8	0.82	0.83	0.9	0.69	0.32

The scaled parameter estimates of the final model are shown in Table 2. After adjusting for spatial and temporal covariances, the variables that remained significantly associated with TBE incidence in Sweden arranged by predictive capacity were mean temperature in the third quarter of the previous year, population, habitat richness (a proxy for vertebrate host diversity), percentage of forest cover, third quarter precipitation, second quarter relative humidity and roe deer density. Mean temperature, population density, habitat richness, precipitation, and higher density of roe deer population were associated with higher TBE risk, while higher forest cover and relative humidity were associated with lower TBE risk (Table 2).

**Table 2:** Final selected scaled model parameter coefficients with 95% Bayesian credible interval ranked by predictive contribution based on Deviance Information Criterion (DIC), Sweden, 2005–2023

	𝝱	Credible Interval		DIC
		Lower	Upper	𝚫
Mean temperature Q03 LAG1	0.455	0.327	0.584	56.775
Log (Population)	1.158	0.887	1.429	51.622
Habitat richness	1.122	0.838	1.408	41.155
Forest cover (%) Q03	-0.284	-0.382	-0.187	25.595
Precipitation (mm) Q03	0.082	0.039	0.126	4.982
Relative Humidity (%) Q02	-0.102	-0.157	-0.047	4.673
Roe deer density	0.173	0.095	0.252	-9.359

*Q* Quarter, *LAG 1:* Previous year, DIC Full model=10900.4

**Fig. 2:**
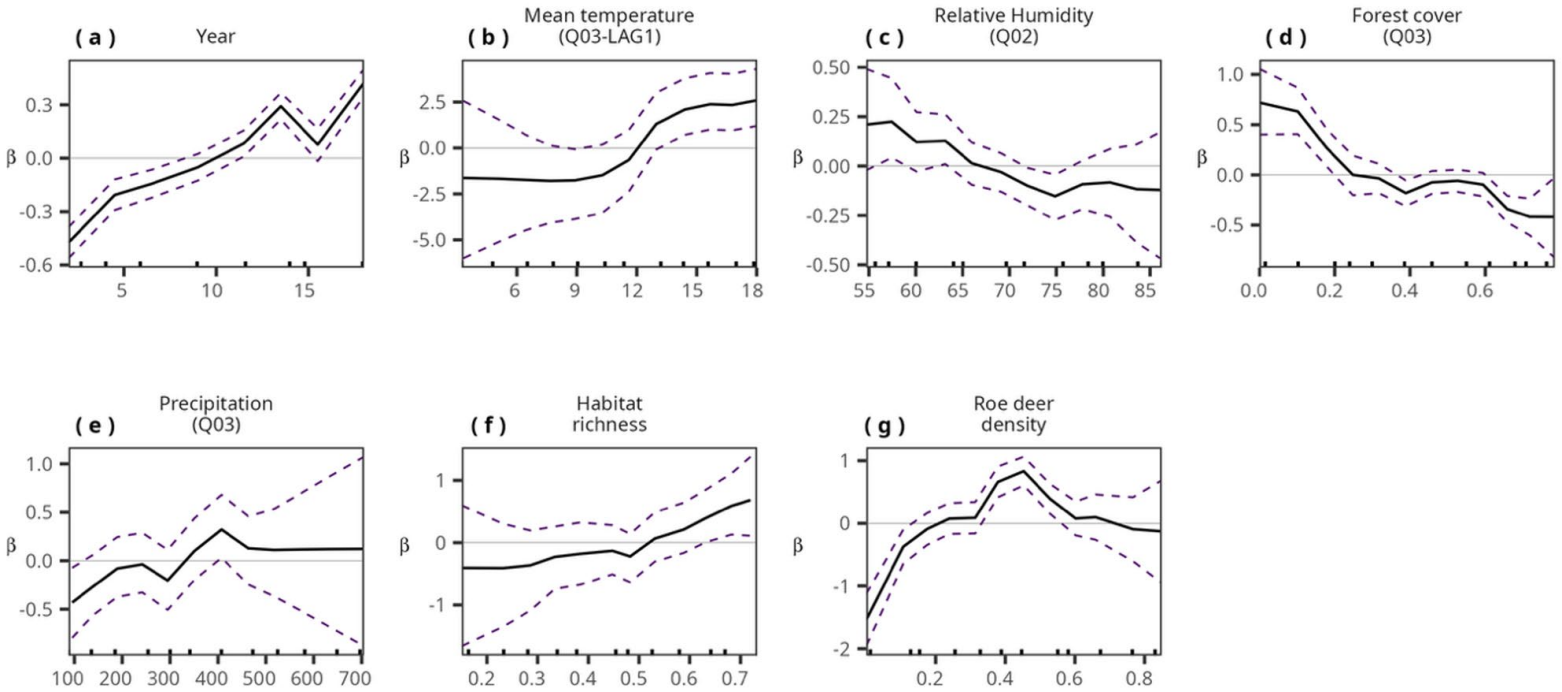
The nonlinear estimated effect of (**a**) year, (**b**) mean temperature in quarter three of the previous year, (**c**) Relative humidity in quarter two of the current year, (**d**) % forest cover in quarter three of the current year, (**e**) third quarter precipitation in the current year, (**f**) Habitat richness and (**g**) Roe deer density on TBE intensity in Sweden. The dotted lines represent the 95% credible interval

Including the identified covariates as non-linear terms in a final model resulted in better predictive capacity and we were able to identify temperature, precipitation, habitat richness and roe deer density threshold values associated with elevated TBE risk (Fig. 2 and Table S18). For example, mean temperatures above 12 °C were associated with increased TBE risk (Fig. 2(b)), higher habitat index above 0.6, (see Fig. 2(f)) was associated with higher TBE rates. Roe deer density between 0.35 and 0.5 per km^2^was associated with elevated TBE risk (Fig. 2(g)), while relative humidity below 65% resulted in higher TBE intensity (Fig. 2(c)).

**Fig. 3:**
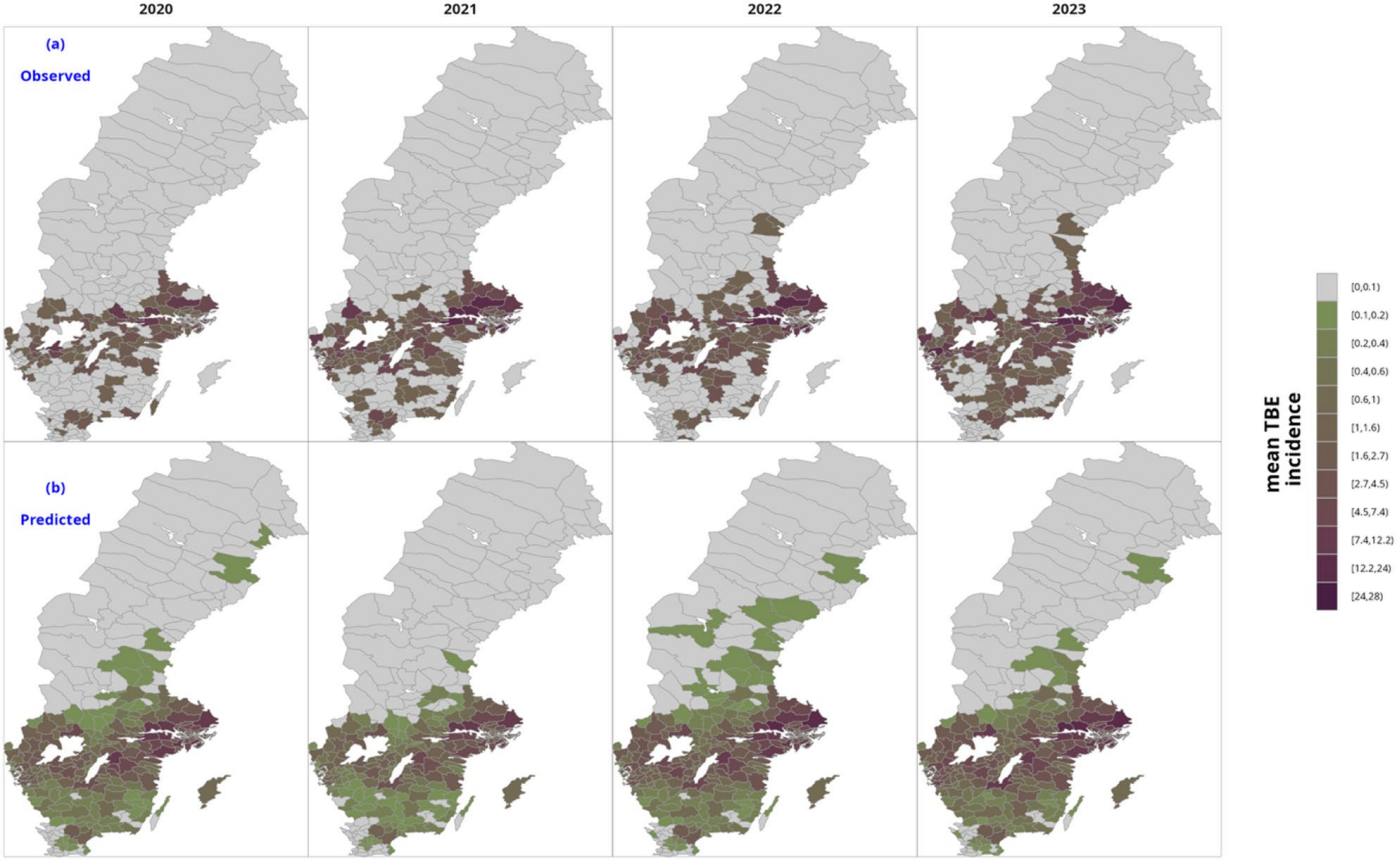
Observed (**a**) vs predicted (**b**) mean annual TBE incidence for model validation period, Sweden, 2020-2023

The final selected model with non-linear covariates performed better than the reference negative binomial regression model and the model with linear effects of covariates, showing model prediction accuracy based on the validation data (Table 3). Overall, for the validation period 2020–2023, the final model had the lowest mean absolute percentage error of 1.26%, compared to 21% for the reference model. The baseline model overpredicted the TBE cases and showed the lowest correlation between predicted and observed at 0.65 compared to 0.72 for the best model. Figure S21 shows the observed and model fitted values in the period 2005–2019 used in generating the model while Fig. 3 shows spatial distribution of observed and predicted TBE cases in the validation period, 2020–2023. The developed model closely matches the observed values both in magnitude and spatially.

**Table 3:** Final TBE model predictive accuracy of TBE incidence, based on the out-of-sample data in comparison to the Baseline model, Linear and Non-linear models, Sweden, 2020–2023

	Year				
	2020	2021	2022	2023	2020–2023
Baseline					
Observed	214	456	395	539	1604
Predicted	455	360	443	448	1706
Correlation	0.46	0.81	0.79	0.77	0.65
MAE					119
MAPE					-21.72%
Linear					
Observed	214	456	395	539	1604
Predicted	266	223	356	586	1431
Correlation	0.56	0.73	0.79	0.8	0.72
MAE					92.8
MAPE					7.01%
Non-Linear					
Observed	214	456	395	539	1604
Predicted	332	214	398	496	1440
Correlation	0.55	0.75	0.81	0.81	0.72
MAE					101.5
MAPE					1.26%

*MAE* Mean Absolute Error (mean difference between model predicted and observed values), *MAPE* Mean Absolute Percentage Error (mean percentage error difference between predicted and observed values)

**Fig. 4:**
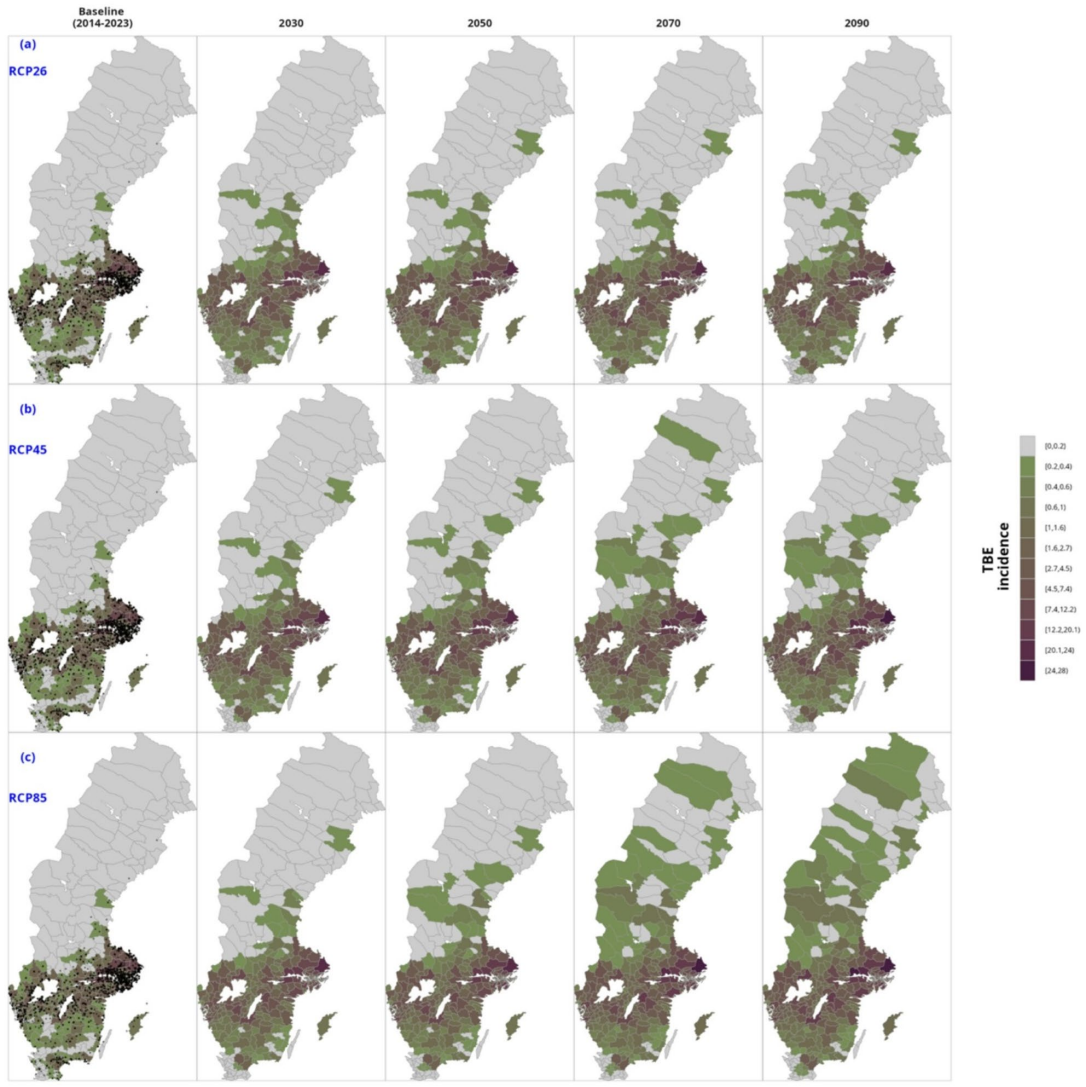
Trends in reported mean annual TBE incidence in Sweden by period and RCP. The points in the baseline maps are the actual geographical points of the cases. Figures (**a**), (**b**) and (**c**) are the decadal projected mean annual TBE cases for the period 2030–2090 for RCP26, RCP45 and RCP85 respectively based on ensemble mean of the contributing regional climate models

In Supplementary Table S10-16, we show the projected TBE cases as well as ensemble projection summaries of covariates in the final model by RCP and RCMs. Figure 4(a), 4(b) and 4(c) shows the spatial distribution of the projected TBE cases by emission scenarios. From the RCM ensemble means, the mean temperature in quarter one for the whole of Sweden (see Table S10) is projected to increase from − 2.16 (2.26) in the 2020s to ໿2.43 (1.72) in the 2090s in the most pessimistic emission scenario RCP85 and to 0.07(2.11) in the middle-of-the-road scenario, RCP 45.

Regionally, the Southern part of Sweden will see quarter one mean temperatures rising to 4.52 (1.64) according to RCP85, (Table S14). Maximum temperatures in the third quarter will rise from 20.29 (1.51) in 2020s to 24.25 (1.79), about 4 degrees rise in the Southern part of Sweden based on the high greenhouse gas emission scenario, (Table S14). The population in Sweden is projected to rise from over 10 million in the 2020s to over 13 million in the 2090s. Based on these projected increases in temperatures and population, we project higher TBE incidences in the future in all the emission scenarios. We show strong correlations between projected temperature increases and high projected TBE burden (Figure S10). In the 2090s, mean annual TBE cases of ໿552.05(73.38) cases have been projected compared to ໿480.56(94.41) in the 2020s based on RCP26, 15% increase while an almost 48% increase according to RCP85 (Table S10).

**Fig. 5:**
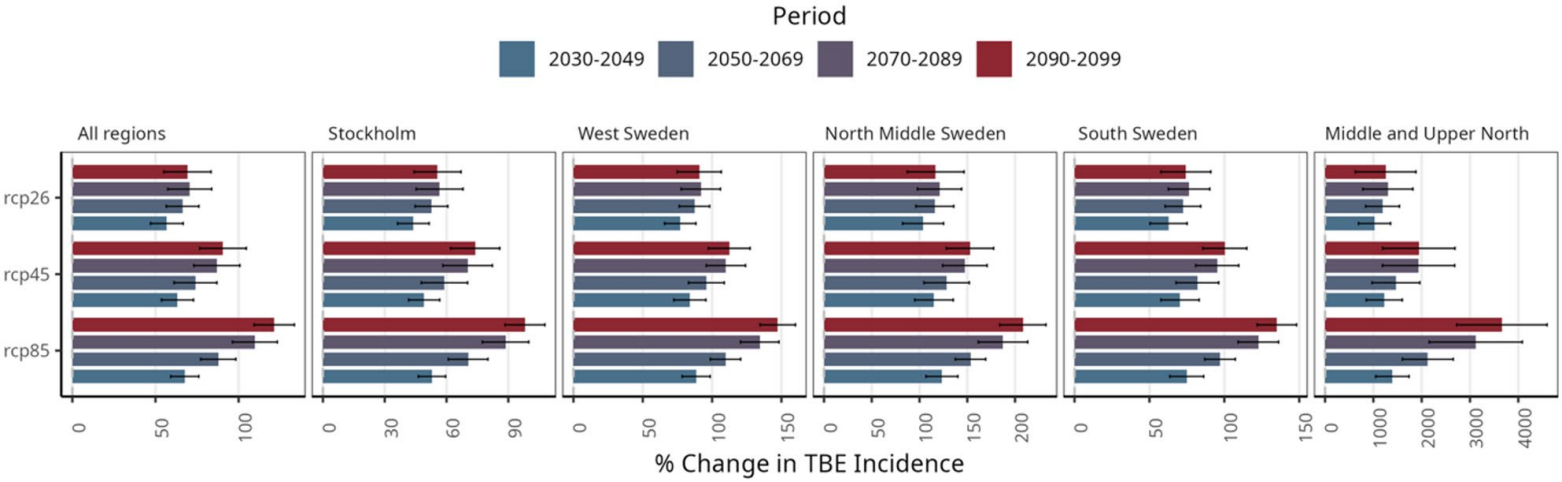
Percentage change in the projected TBE cases by regions in Sweden for all the RCP26, RCP45, and RCP85, in comparison to the baseline period 2014–2023. The error bars were computed based on projections from each contributing regional climate model. The bars represent the ensemble projected mean TBE cases from all the climate models

Figure 5 shows the percentage change in projected TBE, compared to the baseline period (2014–2023). The highest relative increase is observed in higher latitudes. We project over 3000% increase in TBE incidence in Middle and Upper North Sweden according to RCP85, while overall in Sweden, the incidence will increase by over 121% in the 2090s. In Fig. 5, a 4% increase in temperature is projected under emission scenario RCP45 and is associated with a 3.7% rise in TBE incidence rate per decade. Similarly, a 9% decadal increase in mean temperature is correlated with a 7% rise in TBE risk per decade in the highest emission scenario (RCP85). The regional decadal shifts in temperature and TBE risk by RCP and region are shown in Figure S20.

**Fig. 6:**
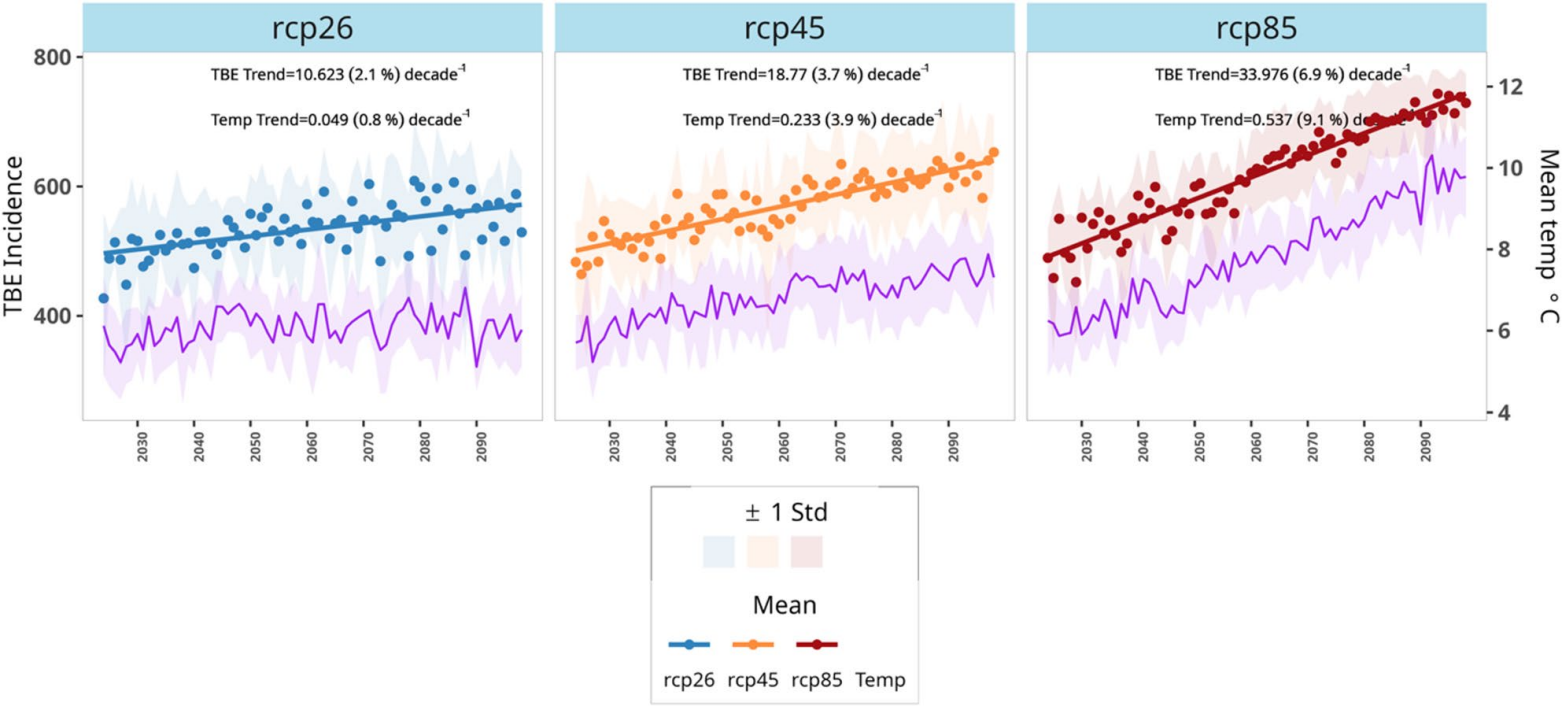
Estimated decadal linear trends in projected TBE incidence and mean temperature for RCP26, RCP45 and RCP85 for the period 2024–2090 for the whole of Sweden. The shared regions represent uncertainty (*±* 1) standard deviations computed from the contributing regional climate circulation models

The regional trends in TBE incidence by RCP are shown in Figure S19, where high incidence rates have been projected for regions in Southern Sweden, with the Stockholm region taking the lead, while decadal spatial projected map of mean annual TBE cases by RCP at the municipality level are shown in Fig. 5 contrasting with the baseline 2014–2023 period. We show increased TBE incidence by municipality as well as increased intensity in the South by decade and RCP. Figure 6 juxtaposes projected TBE incidence with mean temperature by RCP for the coming decades and documents a successive increase in TBE incidence as a function of climate change.

## Discussion

This study provides new and policy-relevant evidence that climate change is likely to substantially amplify the burden of TBE in Sweden over the coming decades, while also reshaping its spatial distribution. Leveraging geocoded, high-resolution Swedish TBE data we employed a Bayesian INLA framework with latent Gaussian processes to quantify the contribution of eco-climatic predictors and capture fine-scale spatiotemporal dynamics. Our analysis revealed that higher third quarter mean temperature, population density, rich habitat diversity, precipitation, and roe deer density—ranked by predictive strength—were strongly associated with elevated TBE transmission risk in Sweden. In the absence of high-resolution data on vertebrate host species, we employed alternative data sources, drawing on citizen science records of roe deer occurrences and measures of habitat richness as proxies. Within our modeling framework, roe deer were utilized as an indicator of wildlife–human interactions, which has been implicated in TBE risk [38–41]. Although roe deer are incidental, dead-end hosts and incompetent reservoirs, citizen science–derived roe density data reflect regions where both roe deer and humans overlap. Habitat richness was incorporated as a proxy for the diversity and suitability of host and vector communities [9]; both proxies were positively associated with TBE risk in Sweden. In contrast, greater forest cover and relative humidity were linked to reduced risk in the Swedish setting.

Most notably, our study offers quantitative, spatially explicit projections of future TBE incidence in Sweden under multiple greenhouse gas emission scenarios, while explicitly accounting for nonlinear environmental thresholds. Our results indicate a consistent rise in TBE incidence across all scenarios, with model outputs predicting by the 2090s a 62% increase in cases under low-CO₂ pathways and up to a 118% increase under high-CO₂ conditions, relative to the 2014–2023 baseline. These findings underscore the strong correlation between future warming and TBE expansion, in line with prior evidence linking climate change to tick range expansion [17, 22, 42]. One of the most striking findings of this study is the projected redistribution of TBE risk toward higher latitudes. While southern Sweden—and particularly the Stockholm region—is expected to remain the epicenter of TBE burden, our projections indicate that the largest relative increases in incidence will occur in Middle and Upper North Sweden. Under RCP85, these regions are projected to experience increases exceeding 3,000% relative to the baseline period, albeit from a low starting incidence. This northward amplification reflects the strong sensitivity of tick survival and activity to winter temperatures [43]. Warmer winters reduce tick mortality, prolong the questing season, and facilitate the establishment of stable tick populations in areas that were previously climatically marginal [43]. As a result, regions that historically reported few or no TBE cases may transition into newly endemic areas over the coming decades.

Our study demonstrates that projected climate change alone—particularly rising temperatures—can drive substantial increases in TBE incidence even in the absence of major changes in wildlife density. Although roe deer density remained relatively stable during the historical study period, temperature-dependent processes emerged as dominant predictors of future risk. Mean temperature, especially in the third quarter of the previous year, ranked as the strongest predictor of TBE incidence, underscoring the importance of lagged climatic effects on tick development, survival, and virus transmission dynamics. Temperature regulates tick phenology, molting rates, questing activity, and viral replication within ticks [5]. Since tick activity, development, and abundance are tightly regulated by temperature [23, 37], humidity, and precipitation, climatic shifts likely influence both TBEV infection rates in ticks and subsequent human exposure. However, our study did not corroborate laboratory and field studies suggesting that *I. ricinus* nymphs are most active when relative humidity exceeds ~ 45% most likely because conditions that favor tick biology do not always translate into higher human disease incidence. In Sweden, higher relative humidity often corresponds to cooler, wetter, densely forested environments where human population density is lower, reducing opportunities for tick–human contact, and by extension TBE risk.

Sweden has already witnessed a marked rise in TBE cases over recent decades [13], a trend mirrored in other endemic European regions. Prior studies reinforce our findings: for instance, research in southern Sweden identified high TBE likelihood in forested wetland, aligning with our observed association with vegetation [44].

By incorporating nonlinear effects, our model identified clear climatic and ecological thresholds associated with elevated TBE risk (Fig. 2). Mean temperatures (Q03 LAG1) above approximately 12 °C, habitat richness above 0.5, and precipitation (Q03) above 350 mm were all associated with marked increases in TBE incidence. These threshold effects are particularly important because they imply that relatively modest eco-climatic shifts can push local environments past critical tipping points, resulting in disproportionately large increases in human disease risk. Such nonlinearities are rarely captured in traditional regression-based surveillance analyses and represent a methodological advance with direct implications for risk forecasting.

Our findings have direct applications in public health practice. Areas identified as high risk can be prioritized for intensified surveillance, vaccination, and citizen engagement. Sweden’s “Rapportera Fästing” platform, which allows the public to submit ticks for identification and TBEV testing, exemplifies the potential of citizen science [45, 46]. Risk maps generated here can further guide such initiatives, directing both tick collection and milk testing from farms in high-risk zones. Detection of positive milk samples would highlight active TBEV transmission and signal a need for strengthened livestock prophylaxis and human vaccination campaigns. Similarly, syndromic surveillance can be refined to focus on projected high-risk regions, providing earlier warning and more targeted alerts.

Despite these advances, underreporting remains a challenge. Many TBEV infections are mild or subclinical and never formally diagnosed, leading to underestimation of disease burden [47]. Our projections therefore help delineate regions likely to become highly endemic, even if notification data understate true incidence [14, 48–50]. Targeted vaccination—including subsidized programs already adopted in several Swedish regions since 2018—should be expanded in such areas. In fact, starting in 2018, the regions of Sörmland, Västmanland, Uppsala, Jönköping, Östergötland, and more recently Stockholm, introduced subsidized TBE vaccination for children. While vaccination campaigns are politically and socially contingent, our projections highlight where such interventions would be most impactful. In Sweden, general TBE vaccine compliance is relatively high, in contrast to other parts of Europe, where it is uneven [48]. Limitations of this analysis include the exclusion of socioeconomic factors, vaccination coverage, reservoir host dynamics, and human behavioral patterns. In particular, increased vaccination uptake could partially offset the projected rise in incidence, while changes in outdoor recreational patterns could either amplify or dampen exposure. Nonetheless, these projections offer valuable foresight, enabling prioritization of prevention efforts under climate change.

Ultimately, effective TBE control in Europe requires an integrated approach, bringing together the public, healthcare providers, and veterinary services. Citizen science platforms, syndromic surveillance, and milk monitoring can now be directed more strategically using our risk maps. Public outreach and education remain vital to increasing vaccination uptake, improving risk perception, and promoting protective practices against tick bites.

As TBE incidence is projected to rise in the coming decades, our study provides critical insights to inform targeted surveillance, vaccination strategies, and preventive measures. Given its capacity to cause severe central nervous system disease with long-term sequelae, TBE poses a growing public health challenge—one that can be mitigated through proactive, data-driven interventions guided by the evidence presented here.

## Supplementary Information

Supplementary Material 1.

## Data Availability

No datasets were generated or analysed during the current study.
