# Supplementary material for: Projecting tick-borne encephalitis risk in Sweden under climate change scenarios: a high-resolution spatio-temporal modeling approach
Source: Environ Health. 2026 Mar 23;25:21. doi: 10.1186/s12940-026-01278-8 (PMC13020375; doi:10.1186/s12940-026-01278-8)
Supplement: Supplementary file 1 — Supplementary Material 1. [file 12940_2026_1278_MOESM1_ESM.pdf]

Projecting Tick-Borne Encephalitis Risk in Sweden under Climate Change Scenarios :
A High-Resolution Spatio-temporal Modeling Approach

Maquins Odhiambo Sewe, Jonas Wallin, Joacim Rocklöv,Shiyu Wang , Torben Koenigk and Jan Semenza

1 Methods

1.1 Data

We used tick-borne encephalitis (TBE) data reported by the public health agency of Sweden for the years 2005 to 2023. In Sweden, TBE is a reportable disease, and the place of likely exposure/infection is recorded (address, if known). Of these, 96% TBE infections were acquired in Sweden, 2.1% abroad and 1.6% had an unknown site of infection. The Swedish TBE cases for the period 2005-2017 comprised individual cases with geographic coordinates and date of reporting while the data for the period 2018-2023 was reported at municipality level. We used municipality centroid points as the spatial coordinates for the latest data covering the period 2018-2023. The TBE incidence data was aggregated by year and only cases reported in Sweden were included. To model the risk of TBE, we included satellite derived climatic factors, land cover, elevation, roe deer density and habitat richness as shown in Table S1. In Table S2 we briefly give justification for the inclusion of each of the covariates used in building the TBE incidence model. The climatic factors used to model TBE incidence included gridded daily temperature (minimum, maximum and mean), relative humidity and precipitation on a spatial resolution of 0.1° by 0.1° which were downloaded from E-OBS [1] gridded datasets. Land use factors derived from the European Union's Copernicus Land Monitoring Service information datasets included indices of vegetation; normalised difference vegetation index (NDVI) [2, 3], leaf area index (LAI) [4, 5] and forest cover (%) [6, 7] at both 300m and 1km spatial resolutions . The high resolution (50m) elevation data was provided by Lantmäteriet and downloaded through dike.slu.se/get based on the Swedish national elevation model. In addition, population data at Municipality for the period 2005-2023 was downloaded from https://www.scb.se. The population data was spatially smoothed. The ticks that spread TBE often feed on blood from roe deer, so we also included annual roe deer density as a covariate. Since roe deer are not capable of effectively transmitting and spreading the virus they are considered indicator hosts in our analysis [8]. High-resolution (100m) maps of roe deer density were obtained from the web-based citizen science platform (Rapportera vilda djur) by the Swedish National Veterinary In-

stitute [9]. Because these roe deer data were derived from citizen scientists, the data represent human-roe deer interactions rather than roe deer density alone. The habitat types data at 10km by 10km grid was downloaded from EEA website [10]. To compute habitat richness index ,the total number of unique habitats per grid cell was divided by the total grid area following methodology used by Cervellini et al in [11].

To project the impact of climate change on TBE incidence in Sweden, we included climate data from future projections with Regional Climate Models (RCMs) considering three greenhouse gas emission scenarios, the optimistic, low emission scenario RCP26, the intermediary RCP45 and the extreme, high-emission scenario RCP85 for the period 2024-2098 provided by Swedish Meteorological and Hydrological Institute (SMHI) `https://www.smhi.se/` (see Table S3). The RCMs were forced by lateral and lower boundary conditions from the following Global Circulation Models (GCMs): CNRM-CERFACS-CNRM-CM5, ICHEC-EC-EARTH, MOHC-HadGEM2-ES, MPI-M-MPI-ESM-LR and NCC-NorESM1-M. The RCMS included were: CNRM-ALADIN63, KNMI-RACMO22E, CLMcom-CCLM4-8-17, GERICS-REMO2015, SMHI-RCA4, DMI-HIRHAM5 and MPI-CSC-REMO2009 with variants r1i1p1,r12i1p1,r3i1p1 and r2i1p1 with versions v2,v1 and v1a, resulting in 51 combinations considering the three emission scenarios. We only included an RCM if they had the three RCPs combinations. The RCMS were biased corrected using EOBS observational data. The population projection data for the period 2024-2070 was downloaded from https://www.scb.se/. The population data by municipality was extrapolated using spline functions to the year 2098 to cover the climate data period.

1.2 Quarterly indicators

For each of the environmental covariates; min temperature, mean temperature, max temperature, precipitation, relative humidity, NDVI, LAI, and forest cover we computed the annual quarterly indicators, see Table S4. The first quarter included the months (January-March), second quarter (April-June) ,third quarter (July-September) and fourth quarter (October -December). For precipitation we computed the totals while for other factors we computed the averages. In addition to the annual estimates, we computed lags of the quarterly variables for the previous year. For the other indicators (habitat richness and roe deer density), we did not compute quarterly indicators.

1.3 Statistical modelling

The annual TBE point data were modeled as a log-Gaussian Cox process, a hierarchical Poisson process with random intensity assuming a latent Gaussian random field. The covariance structure of the Gaussian latent random field can be estimated using the computationally efficient integrated nested Laplace approximation (INLA) approach developed by Rue et al [12] and Lindgren et al [13] within a Bayesian framework in R environment [14]. Specifically we

used INLA_25.10.19 and R 4.5.2[15] versions for this analysis. Traditionally the log-Gaussian Cox process has been modeled assuming that the cases occur on a regular grid within a defined spatial space, a computationally inefficient and inflexible approach. We instead followed the approach suggested by Simpson et al [16] that utilizes a finite dimensional continuously indexed random field (mesh) proposed by Lindren et al [13] to estimate the Gaussian random field. In this case, the data is modeled taking into account the exact location instead of binning as in the case with regular grid. In this framework if we assume a bounded region $\phi \subset R^2$, then an inhomogeneous Poisson process occurs when the number of points in the sub region $A \subset \phi$ is Poisson distributed with mean $\Lambda(A) = \int \lambda(s)ds$, $\lambda(s)$ is the intensity surface of the Poisson point process. Taking the intensity surface as a realization of a random field $\lambda(s)$ results in a class of point process referred to as the Cox Poisson process. These models are useful when you have both observed and unobserved environmental variation [16] . For the TBE analysis we apply the log Gaussian Cox process, where the intensity is modeled considering the Gaussian random field, i.e $\log(\lambda(s)) = Z(s), Z(s)$ is the Gaussian random field. The latent Gaussian field is then approximated using stochastic partial differential equations [13]. We describe the modelling steps below.

1.4 Constructing of the mesh

To define the Gaussian random field, we constructed a triangulated mesh covering the whole of Sweden as shown in Figure S3. using mesh creation functions within R-INLA [14] . From the mesh vertices we generated dual polygons and calculated the volumes of each polygon within the spatial domain. The polygon volumes were included in the Poisson model as the expected number of cases. To create the mesh, we consider three important parameters. The max edge which controls the maximum length of the mesh vertices (this controls the resolution of the mesh), the offset value which determines how much the domain can be extended and the cutoff value used to avoid having several vertices within clustered data points. For the TBE model, we chose a maximum edge of 42km for the inner domain, 210km for the outer domain and a cutoff value of 9km. This resulted in 1379 mesh locations. Sensitivity analysis following suggestions by [17] was used in the selection of the best mesh configuration. We further conducted spatial aggregation of the TBE points to the dual polygons. To include covariates in the model, we extracted covariate values at the triangulated mesh nodes as well as at the observed TBE points. We assume a Matérn covariance structure for the random field which is defined by the spatial range s and the marginal variance (σ^2). The quarterly indicators which were derived from same input datasets meant some of the variables would be highly collinear. To deal with this, we selected the best indicator with the best predictive potential for each variable included based on DIC, see Table S16 showing how we selected the variables. For each covariate category for example temperature we selected the top ranked indicator based on DIC.

1.5 Model selection

The first level of the hierarchical structure is the intensity of TBE cases in each polygon captured by Poisson likelihood. The second level involves selection of the linear predictor part of the model as detailed below. First, we included each covariate (the environmental indicators) one by one in the base model which includes the intercept, spatial random effect, elevation, the year random effect and log population density. Note that we include population as a covaraite in the model and not as an offset to distinguish between its role as a denominator and its ecological impact on tick populations. For each class of covariate, we selected best one based on DIC, see Table S16. From the selected varaibles , variance inflation factor (VIF) was computed to test for multicollinearlity and remove highly collinear variables. This was done using functions in R package usdm [18]. Using a threshold of 2.5, elevation and NDVI were dropped due to high collinearlity, See Table S17. The selected indicators were then included in a joint model that made up the final model. The equations 1-6 below, complete the Bayesian hierarchical structure of the model.

$$\pi\left(\mathbf{Y}|\lambda\right) = exp\left(T\left|\mathcal{D}\right| - \sum_{i=1}^{T}\int\lambda\left(\mathbf{s},t\right)d\mathbf{s}\right)\prod_{i=1}^{N}\lambda\left(s_i,t_i\right) \tag{1}$$

$$\log\left(\lambda\left(\mathbf{s},t\right)\right) = \beta_0 + \mathbf{X_{s,t}}\beta + f(t) + w\left(\mathbf{s},t\right) \tag{2}$$

$$f(t) \sim \mathcal{N}\left(\mathbf{0}, r_{time}\right) \tag{3}$$

$$w\left(\mathbf{s},t\right) \sim \mathcal{N}\left(\mathbf{0}, r_{space}\right) \tag{4}$$

$$\pi\left(\beta_0\right) \propto 1 \tag{5}$$

$$\beta \sim \mathcal{N}\left(\mathbf{0},\mathbf{I}\right) \tag{6}$$

The first equation 1 in the specification denotes the likelihood of a Poisson process with intensity λ, and Y are a vector of N observations locations in space and time, denoted by $\{s_i, t_i\}_{i=1}^{N}$. The log intensity is modeled by a random effect of year $f(t)$, modeled by a Gaussian random walk of order 2 to capture annual trends in TBE cases and for each year a spatial random effect, $w(s,t)$.The spatial effect is a Gaussian Matérn random field with an independent Gaussian for each normalsize. X_s are the covariates included in the model. A flat prior was specified for the intercept β_0, fixed effects β_j are assumed independent with gamma prior distributions. We specify penalized complexity (PC) priors for the spatial random effect $w(s,t)$ following Fuglstad et al [19] . We used the mesh configuration to specify the PC priors for the hyperpriors for the spatial effect, namely the spatial range and standard deviation.

The final model chosen is described in the equation 7.

$$\log(\lambda_s) = \beta_0 + \beta_1(LogPopDensity) + \beta_i \sum_{i=1} X_i + f(t) + w(s,t) \quad (7)$$

Where β_0 is the intercept, β_1 is the coefficient for the log smoothed population density, $\beta_i$is the coefficient for the covariates chosen through variable selection. The final model is shown in equation 8 below.

$$\begin{aligned} \log(\lambda_s) = \beta_0 + \beta_1(LogPopDensity) + \\ \beta_2(tmean\_Q03\_LAG1) + \beta_3(Habitat\_richness) + \\ \beta_4(fcover\_Q03) + \beta_5(prec\_Q03) + \\ \beta_6(RH\_Q02) + \beta_7(roe\_deer\_density) + f(t) + w(s,t) \end{aligned} \quad (8)$$

After variable selection, we further developed another model including the selected covariates as nonlinear terms, which were modelled using random walk of order 1. We tested the influence of each selected predictor on the models predictive capacity by iteratively removing each one and computing the effect on the overall model Deviance Information Criterion (DIC) value. We then ranked them based on contribution to the overall predictive capacity.

1.6 Prediction on a regular grid

To perform predictions for a particular year accross the whole of Sweden, we first extracted the covariates in the final model at the mesh vertices for that year, set the outcome which in this case is the TBE intensity as NA. Since In INLA, the prediction is done simultaneously with model estimaton, we created as stack of the model and predicton data using the stack function in INLA. This was then fed into the INLA model to predict the TBE intensity at the mesh vertices. Once we had the predictions at the mesh vertices, we projected predictions on a regular grid, 5km by 5km using the INLA projection functions.

1.7 Model validation

Based on the selected model above, we evaluated the models' predictive accuracy by doing out-of-sample predictions for the years 2020 to 2023. Iteratively, we made predictions on a regular grid (5km X 5km) for each year in the validation set based on data for the preceding year's excluding the prediction year. The predictions were made simultaneously with the estimation process. This was achieved by setting the response of prediction points to missing. The posterior mean intensity was then projected on the finer 5km by 5km grid. We then aggregated the posterior predicted cases (the product of the intensity and the area of each grid) to municipality level and computed prediction metrics such as correlations, mean absolute error between the observed and the predicted cases. As comparative reference model, we built a negative binomial model of the annual TBE cases aggregated at municipality level. The reference model

included previous TBE cases and smoothed population covariate, following the approach used in building TBE predictive models in Poland [20].

1.8 Climate impact projections

The final selected model was used to project potential impact of climate change on TBE incidence. We follow similar steps in the in the out of sample validation process to project TBE intensity for each RCP scenario. We set values to the 2023 values for covariates without future scenarios datasets such as roe deer density, the land use (forest cover and habitat richness) variables and Relative Humidity. We perform future projection for each of the combination of RCMs and emission scenarios (RCP) from 2020 to 2090. We present percentage change in mean TBE incidence relative to the baseline years (2014-2023) as a measure of impact. We aggregate and compare the results at generated regional grouping, see Figure S4.

2

Table S1: Data sources

	Resolution			
	Spatial	Temporal	Units	Source
**Outcome**				
TBE cases	point	daily	Count	Public health agency Sweden(folkhalsomyndigheten)
**Population**				
**Population** density	point	yearly	per km^2	SCB sweden
**Climate**				
Min temperature	0.1	daily	$^\circ$C	E-OBS gridded dataset
Max temperature	0.1	daily	$^\circ$C	E-OBS gridded dataset
Mean temperature	0.1	daily	$^\circ$C	E-OBS gridded dataset
Precipitation	0.1	daily	mm	E-OBS gridded dataset
Relative Humidity	0.1	daily	(%)	E-OBS gridded dataset
**Land use**				
NDVI	1km	daily		Copernicus land Service -NDVI 1km v.2.2 product
Forest cover	1km	daily	(%)	Copernicus land Service FCOVER product
Leaf Area Index	1km	daily		Copernicus land Service LAI product
Elevation	1km		Metres (m)	Lantmäteriet
Habitat Richness index	10km			European Environment Agency (EEA)
**Roe deer density**				
Roe	1km	yealy	per km^2	SLU

NDVI:Normalized difference vegetation index
SLU:Sveriges lantbruksuniversitet
Forest cover:Fraction of green vegetation cover

Table S2: Variable inclusion reason

Predictor	Type	Reason for inclusion	References
Population	Socio-Demographic	Population is used as denominator to compute incidence rates. Higher population density could also increase risk to TBE infection in endemic areas.	[21, 22]
Temperature ($^{\circ}$C)	Climate	Temperature highly influences the development and abundance of ticks that transmit TBE in humans. Higher temperatures speed TBEV virus in ticks and the rate of transmission to susceptible host. A threshold of 5 $^{\circ}$C is needed to sustain tick activity and start the vegetation season. Temperature between 8-10 $^{\circ}$C is required for egg hatching and tick metamorphosis. Different indicators of temperature have been used to model TBE incidence including bioclimatic indicators such mean of temperature of the driest month, autumn cooling etc.	[23–26]
Precipitation (mm)	Climate	Precipitation just like temperature modulates the development and survival of ticks. Rainfall is associated with nymph oviposition rates with higher precipitation reducing tick abundance due to silting of egg masses. Precipitation levels also impact changes in forest and wildlife density.	[23, 25, 27]
Relative humidity (RH) (%)	Climate	Ticks require higher RH to survive with a threshold of 90%. High risk of desiccation is observed in ticks when RH goes below 80%.	[21, 26, 28, 29]
Normalized difference vegetation index (NDVI)	Environmental	NDVI indirectly influences TBE risk as it's a proxy for vegetation types and density. Higher vegetation may provide suitable habitat for ticks to proliferate as it sustains tick host population.	[21, 22, 30]
Leaf area Index (LAI)	Environmental	LAI a proxy for habitat structure influence TBE risk by influencing tick abundance. Higher LAI , for example dense canopy provide suitable environment for ticks to survive. Areas with moderate to high LAI provide ideal habitat complexity for both ticks and their hosts.	
Forest cover (%)	Environmental	Forest type and density affect tick abundance. They are a source of moisture and sustenance for competent TBE mammalian host. Forests also increase human contact rates with ticks. A higher proportion of mixed forests and coniferous forests have been shown to strongly correlate with TBE cases.	[25, 31, 32]
Elevation (m)	Environmental	Elevation is an important factor for the presence of tick vectors that transmit TBE. Higher altitudes which are associated with lower temperatures limit the range of tick vectors.	[21, 22, 29]
Habitat index	Environmental	Habitat richness, a proxy for biodiversity, is significantly linked to TBE incidence in Europe. Intermediate values of habitat richness index (HRI) elevate TBE risk while low and higher values have protective effect. In Europe high HRI reduces risk of TBE given its high biodiversity with a consequence that most habitats are unsuitable for ticks and host.	[33]
Roe deer density	Vertebrate Host	Roe deer density has been shown to highly correlation with TBE incidence as they are a major source of blood meal for adult female ticks. Roe deer infestation with co-feeding ticks is significantly associated with TBE risk.	[31, 34–37]

Table S3: Projections data sources

GCM	RCM	variant	version	RCP	Spatial resolution	Time
CNRM-CERFACS-CNRM-CM5	CNRM-ALADIN63	r1i1p1	v2	rcp26,rcp45,rcp85	0.11	1970-2098
CNRM-CERFACS-CNRM-CM5	KNMI-RACMO22E	r1i1p1	v2	rcp26,rcp45,rcp85	0.11	1970-2098
ICHEC-EC-EARTH	CLMcom-CCLM4-8-17	r12i1p1	v1	rcp26,rcp45,rcp85	0.11	1970-2098
ICHEC-EC-EARTH	GERICS-REMO2015	r12i1p1	v1	rcp26,rcp45,rcp85	0.11	1970-2098
ICHEC-EC-EARTH	KNMI-RACMO22E	r12i1p1	v1	rcp26,rcp45,rcp85	0.11	1970-2098
ICHEC-EC-EARTH	SMHI-RCA4	r12i1p1	v1	rcp26,rcp45,rcp85	0.11	1970-2098
ICHEC-EC-EARTH	DMI-HIRHAM5	r3i1p1	v2	rcp26,rcp45,rcp85	0.11	1970-2098
MOHC-HadGEM2-ES	DMI-HIRHAM5	r1i1p1	v2	rcp26,rcp45,rcp85	0.11	1970-2098
MOHC-HadGEM2-ES	GERICS-REMO2015	r1i1p1	v1	rcp26,rcp45,rcp85	0.11	1970-2098
MOHC-HadGEM2-ES	KNMI-RACMO22E	r1i1p1	v2	rcp26,rcp45,rcp85	0.11	1970-2098
MOHC-HadGEM2-ES	SMHI-RCA4	r1i1p1	v1	rcp26,rcp45,rcp85	0.11	1970-2098
MPI-M-MPI-ESM-LR	MPI-CSC-REMO2009	r1i1p1	v1	rcp26,rcp45,rcp85	0.11	1970-2098
MPI-M-MPI-ESM-LR	SMHI-RCA4	r1i1p1	v1a	rcp26,rcp45,rcp85	0.11	1970-2098
MPI-M-MPI-ESM-LR	MPI-CSC-REMO2009	r2i1p1	v1	rcp26,rcp45,rcp85	0.11	1970-2098
MPI-M-MPI-ESM-LR	CLMcom-CCLM4-8-17	r1i1p1	v1	rcp26,rcp45,rcp85	0.11	1970-2098
NCC-NorESM1-M	GERICS-REMO2015	r1i1p1	v1	rcp26,rcp45,rcp85	0.11	1970-2098
NCC-NorESM1-M	SMHI-RCA4	r1i1p1	v1	rcp26,rcp45,rcp85	0.11	1970-2098

GCM:Global circulation model
RCM:Regional circulation model

Table S4: Derived quarterly indicators

	Description	Quarter	LAG
Tmin	Minimum temperature	01,02,03,04	01
Tmax	Mean temperature	01,02,03,04	01
Tmean	Maximun temperature	01,02,03,04	01
Prec	Cummulative Precipitation	01,02,03,04	01
RH	Relative Humidity	01,02,03,04	01
Fcover	Forest cover (%)	01,02,03,04	01
NDVI	Normalized difference vegetation index	01,02,03,04	01
LAI	Leaf Area Index	01,02,03,04	01

Table S5: Descriptive summary of TBE cases, Habitat richness,Elevation,Polulation,Roe deer density, climatic and land cover indicators by quarter and period in Stockholm in Sweden 2005-2023

		Period					
	Quarter	2005-2007	2008-2011	2012-2014	2015-2017	2018-2020	2021-2023
**TBE incidence rate**		3.31	4.5	4.31	5.19	4.57	6.46
**Population**		3.44	3.6	3.77	3.93	4.1	4.2
**Elevation**		69.76	69.76	69.76	69.76	69.76	69.76
**Roe deer density**		0.19	0.19	0.2	0.19	0.19	0.19
**Habitat richness**		0.58	0.58	0.58	0.58	0.58	0.58
**Mean temperature**	Q01	-1.94	-2.22	-1.13	-0.03	0.03	-0.08
	Q02	10.43	10.82	10.11	10.17	11.59	10.86
	Q03	15.67	15.17	15.12	15.09	15.83	15.51
	Q04	3.94	2.02	3.34	3.67	4.28	2.5
**Minimum temperature**	Q01	-5.42	-5.34	-4.33	-3.09	-3.05	-3.43
	Q02	5.12	5.2	5.02	5.04	5.53	5.01
	Q03	10.9	10.92	10.19	10.45	10.93	11.04
	Q04	1.28	-0.74	0.63	0.9	1.84	-0.2
**Maximun temperature**	Q01	1.5	0.81	2.11	3.15	3.07	3.4
	Q02	15.79	16.35	15.28	15.35	17.37	16.43
	Q03	20.75	19.88	20.3	20.03	21.03	20.31
	Q04	6.69	4.72	5.94	6.4	6.7	5.15
**Precipitation**	Q01	108.62	108.97	102.05	109.61	116.29	124.4
	Q02	144.59	121.41	173.28	133.51	102.73	121.99
	Q03	187.04	244.38	182.8	185.21	172.44	231.86
	Q04	170.43	168.54	177.25	149.78	176.12	153.63
**Relative Humidity**	Q01	83.18	83.95	82.44	84.78	81.02	79.7
	Q02	71.41	70.88	73.43	71.27	64.46	65.18
	Q03	79.06	81.22	80.07	78.89	74.32	77.07
	Q04	88.25	88.24	88.94	86.87	87.72	86.48
**NDVI**	Q01	0.08	0.09	0.09	0.17	0.2	0.17
	Q02	0.51	0.51	0.52	0.53	0.54	0.57
	Q03	0.61	0.58	0.62	0.61	0.63	0.69
	Q04	0.34	0.32	0.21	0.37	0.27	0.3
**Forest cover**	Q01	0.21	0.21	0.22	0.21	0.2	0.12
	Q02	0.51	0.51	0.53	0.52	0.5	0.47
	Q03	0.57	0.58	0.58	0.59	0.54	0.48
	Q04	0.21	0.22	0.21	0.21	0.16	0.09
**Leaf area index**	Q01	0.74	0.73	0.77	0.78	0.76	0.43
	Q02	2.22	2.22	2.35	2.34	2.08	2.06
	Q03	2.56	2.49	2.62	2.75	2.39	2.31
	Q04	0.78	0.8	0.78	0.82	0.63	0.31

Table S6: Descriptive summary of TBE cases, Habitat richness,Elevation,Polulation,Roe deer density, climatic and land cover indicators by quarter and period in West Sweden in Sweden 2005-2023

		Period					
	Quarter	2005-2007	2008-2011	2012-2014	2015-2017	2018-2020	2021-2023
**TBE incidence rate**		0.88	1.12	0.94	2.02	2.92	5
**Population**		1.83	1.87	1.92	1.99	2.06	2.1
**Elevation**		110.67	110.67	110.67	110.67	110.67	110.67
**Roe deer density**		0.16	0.16	0.17	0.21	0.22	0.22
**Habitat richness**		0.58	0.58	0.58	0.58	0.58	0.58
**Mean temperature**	Q01	-0.83	-1.13	-0.16	0.79	0.99	0.82
	Q02	10.53	10.95	10.4	10.35	11.85	10.93
	Q03	15.52	15.09	15	14.9	15.61	15.46
	Q04	4.9	2.82	4.3	4.68	5.05	3.78
**Minimum temperature**	Q01	-3.99	-3.94	-3.01	-1.78	-1.57	-2.12
	Q02	5.63	5.63	5.6	5.93	6.46	5.75
	Q03	11.17	11.23	10.6	10.99	11.48	11.57
	Q04	2.15	0.24	1.85	2.19	2.86	1.48
**Maximun temperature**	Q01	2.4	1.71	2.85	3.61	3.68	4
	Q02	15.56	16.19	15.34	15.11	17.29	16.17
	Q03	20.24	19.41	19.69	19.32	20.23	19.91
	Q04	7.8	5.43	6.68	7.2	7.39	6.14
**Precipitation**	Q01	180.72	168.14	134.9	173.02	226.76	198.24
	Q02	198.85	147.81	219.43	175.09	134.93	137.9
	Q03	261.92	344.15	249.17	241.77	233.75	303.84
	Q04	273.48	252.58	300.03	242.54	257.9	233.05
**Relative Humidity**	Q01	84.13	85.43	83.59	86.37	85.04	83.26
	Q02	75.81	72.85	75.34	74.54	69.03	69.65
	Q03	81.88	82.62	81.44	81.62	79.02	80.71
	Q04	88.66	88.26	89.57	87.61	89.14	88.34
**NDVI**	Q01	0.14	0.16	0.16	0.24	0.26	0.19
	Q02	0.48	0.5	0.5	0.52	0.54	0.56
	Q03	0.57	0.56	0.58	0.58	0.61	0.66
	Q04	0.35	0.34	0.25	0.35	0.33	0.3
**Forest cover**	Q01	0.21	0.21	0.21	0.22	0.22	0.14
	Q02	0.51	0.51	0.51	0.52	0.52	0.46
	Q03	0.57	0.58	0.58	0.59	0.52	0.45
	Q04	0.22	0.23	0.22	0.22	0.19	0.1
**Leaf area index**	Q01	0.7	0.71	0.74	0.78	0.78	0.5
	Q02	2.23	2.26	2.28	2.34	2.21	2.09
	Q03	2.59	2.59	2.63	2.82	2.38	2.26
	Q04	0.78	0.8	0.78	0.83	0.7	0.39

Table S7: Descriptive summary of TBE cases, Habitat richness,Elevation,Polulation,Roe deer density, climatic and land cover indicators by quarter and period in North Middle Sweden in Sweden 2005-2023

		Period					
	Quarter	2005-2007	2008-2011	2012-2014	2015-2017	2018-2020	2021-2023
**TBE incidence rate**		0.08	0.27	0.44	1.18	2.26	4.81
**Population**		0.82	0.83	0.83	0.85	0.86	0.86
**Elevation**		280.89	280.89	280.89	280.89	280.89	280.89
**Roe deer density**		0.07	0.07	0.07	0.07	0.06	0.06
**Habitat richness**		0.51	0.51	0.51	0.51	0.51	0.51
**Mean temperature**	Q01	-4	-4.99	-3.43	-2.67	-2.91	-3.08
	Q02	8.76	9.23	8.48	8.3	9.77	9.19
	Q03	13.91	13.23	13.33	13.04	13.63	13.55
	Q04	1.38	-0.85	0.62	0.84	1.32	-0.67
**Minimum temperature**	Q01	-8.1	-8.86	-7.14	-6.32	-6.68	-7.21
	Q02	3.05	3.34	3.21	2.99	3.34	2.88
	Q03	8.81	8.7	8.13	8.31	8.46	8.72
	Q04	-1.73	-4.1	-2.4	-2.4	-1.44	-3.8
**Maximun temperature**	Q01	0.13	-1.16	0.31	1	0.76	1.12
	Q02	14.3	14.89	13.73	13.58	15.87	15.08
	Q03	19.2	18.19	18.77	18.09	19.03	18.65
	Q04	4.6	2.36	3.59	4.1	4.11	2.43
**Precipitation**	Q01	113.32	126.58	103.03	122.82	138.12	116.45
	Q02	152.51	160.41	195.18	160.17	131.57	134.44
	Q03	206.23	287.98	214.92	236.72	201.25	291.93
	Q04	197.49	165.85	212.91	146.51	222.88	166.57
**Relative Humidity**	Q01	82.23	83.02	81.97	84.08	80.77	78.69
	Q02	71.12	71.53	72.5	71.53	65.15	65.06
	Q03	79.19	82.11	80.24	80.61	75.99	78.18
	Q04	87.95	87.49	88.51	87.12	87.74	85.29
**NDVI**	Q01	0.04	0.01	0.04	0.06	0.08	0.03
	Q02	0.45	0.45	0.46	0.44	0.46	0.51
	Q03	0.62	0.59	0.62	0.59	0.63	0.71
	Q04	0.28	0.28	0.21	0.35	0.29	0.3
**Forest cover**	Q01	0.25	0.25	0.26	0.25	0.24	0.06
	Q02	0.47	0.49	0.5	0.49	0.49	0.39
	Q03	0.58	0.58	0.59	0.6	0.54	0.47
	Q04	0.25	0.25	0.25	0.26	0.19	0.08
**Leaf area index**	Q01	0.92	0.92	0.97	1.05	1.04	0.21
	Q02	1.87	1.99	2.02	2.04	1.96	1.61
	Q03	2.55	2.53	2.6	2.8	2.44	2.3
	Q04	0.92	0.94	0.98	1.09	0.78	0.3

Table S8: Descriptive summary of TBE cases, Habitat richness,Elevation,Polulation,Roe deer density, climatic and land cover indicators by quarter and period in South Sweden in Sweden 2005-2023

		Period					
	Quarter	2005-2007	2008-2011	2012-2014	2015-2017	2018-2020	2021-2023
**TBE incidence rate**		0.34	0.48	0.53	0.91	1.05	1.81
**Population**		2.14	2.2	2.25	2.33	2.41	2.45
**Elevation**		121.28	121.28	121.28	121.28	121.28	121.28
**Roe deer density**		0.21	0.21	0.16	0.2	0.2	0.2
**Habitat richness**		0.61	0.61	0.61	0.61	0.61	0.61
**Mean temperature**	Q01	-0.61	-0.66	-0.07	0.97	1.26	1.11
	Q02	10.75	10.76	10.43	10.51	11.91	10.91
	Q03	15.58	15.26	15.16	15.06	15.98	15.72
	Q04	4.86	3.12	4.57	4.88	5.39	4.2
**Minimum temperature**	Q01	-3.84	-3.44	-3	-1.82	-1.55	-2.05
	Q02	5.42	5.04	5.23	5.4	6	5.17
	Q03	10.88	11.06	10.36	10.52	11.14	11.23
	Q04	2.1	0.54	2.07	2.36	2.97	1.63
**Maximun temperature**	Q01	2.6	2.13	3.02	3.98	4.08	4.53
	Q02	16.17	16.35	15.69	15.73	17.67	16.47
	Q03	20.63	19.93	20.25	19.96	21.19	20.63
	Q04	7.71	5.76	7.02	7.43	7.87	6.76
**Precipitation**	Q01	145.73	125.18	117.28	126.63	176.69	160.66
	Q02	153.23	130.34	169.78	147.73	99.79	101.51
	Q03	241.53	252.09	194.58	190.93	175.47	230.22
	Q04	182.8	207.45	204.28	205.03	181.29	200.48
**Relative Humidity**	Q01	84.97	85.73	84.09	86.41	84.92	82.52
	Q02	75.99	74.96	75.71	74.56	68.84	69.48
	Q03	82.56	82.98	81.79	81.6	77.62	79.76
	Q04	89.26	88.63	89.45	88.23	88.89	88.56
**NDVI**	Q01	0.18	0.19	0.2	0.29	0.29	0.26
	Q02	0.54	0.56	0.57	0.59	0.61	0.62
	Q03	0.63	0.61	0.63	0.63	0.66	0.72
	Q04	0.44	0.37	0.33	0.37	0.35	0.38
**Forest cover**	Q01	0.23	0.23	0.24	0.24	0.24	0.17
	Q02	0.55	0.57	0.58	0.58	0.56	0.52
	Q03	0.61	0.63	0.63	0.64	0.58	0.51
	Q04	0.24	0.25	0.24	0.25	0.21	0.13
**Leaf area index**	Q01	0.78	0.79	0.84	0.85	0.86	0.6
	Q02	2.39	2.5	2.64	2.63	2.38	2.33
	Q03	2.74	2.84	2.96	3.09	2.63	2.56
	Q04	0.86	0.89	0.88	0.94	0.79	0.47

Table S9: Descriptive summary of TBE cases, Habitat richness,Elevation,Polulation,Roe deer density, climatic and land cover indicators by quarter and period in Middle and Upper North in Sweden 2005-2023

		Period					
	Quarter	2005-2007	2008-2011	2012-2014	2015-2017	2018-2020	2021-2023
**TBE incidence rate**		0	0.06	0	0	0	0.11
**Population**		0.88	0.88	0.88	0.89	0.9	0.9
**Elevation**		440.65	440.65	440.65	440.65	440.65	440.65
**Roe deer density**		0.01	0.01	0.01	0.01	0.01	0.01
**Habitat richness**		0.49	0.49	0.49	0.49	0.49	0.49
**Mean temperature**	Q01	-8.57	-9.25	-7.86	-6.98	-8.49	-7.73
	Q02	5.6	5.85	5.25	4.82	6.02	5.92
	Q03	11.35	10.9	11.24	10.74	11.13	11.21
	Q04	-2.26	-4.23	-3.83	-3.15	-2.74	-5.25
**Minimum temperature**	Q01	-13.26	-13.65	-12	-11.21	-13.03	-12.37
	Q02	0.83	0.98	0.52	0.21	0.61	0.66
	Q03	6.81	6.71	6.73	6.48	6.55	6.99
	Q04	-5.49	-7.59	-7.16	-6.54	-5.75	-8.67
**Maximun temperature**	Q01	-4.08	-5.13	-4.06	-3.05	-4.22	-3.49
	Q02	10.24	10.6	9.91	9.37	11.23	10.94
	Q03	16.15	15.45	15.99	15.28	15.98	15.74
	Q04	0.83	-1.02	-0.7	0.13	0.16	-2.02
**Precipitation**	Q01	104.43	117.08	110.24	123.29	129.82	123.2
	Q02	141.68	150.03	142.01	149.03	118.96	125.6
	Q03	228.8	239.69	212.19	240.11	205.55	255.82
	Q04	178.2	149.93	167.02	154.75	169.17	151.44
**Relative Humidity**	Q01	81.63	83.55	83.87	83.92	81.53	80.18
	Q02	70.82	72.31	72.52	72.13	67.95	67.45
	Q03	78.44	80.91	79.9	81.13	77.37	78.17
	Q04	87.19	87.54	88.18	88.37	87.24	85.83
**NDVI**	Q01	-0.06	-0.06	-0.06	0	0	-0.07
	Q02	0.22	0.23	0.23	0.21	0.25	0.28
	Q03	0.58	0.58	0.58	0.57	0.5	0.72
	Q04	0.14	0.13	0.11	0.11	0.08	0.2
**Forest cover**	Q01	0.2	0.19	0.2	0.21	0.21	0
	Q02	0.35	0.36	0.37	0.35	0.36	0.24
	Q03	0.5	0.5	0.51	0.52	0.47	0.43
	Q04	0.2	0.2	0.21	0.23	0.16	0.04
**Leaf area index**	Q01	0.68	0.68	0.73	0.82	0.82	0.01
	Q02	1.23	1.28	1.36	1.27	1.3	0.96
	Q03	1.97	1.99	2.02	2.14	1.88	1.94
	Q04	0.65	0.67	0.73	0.83	0.56	0.14

Table S10: Ensemble Mean of TBE cases,Tbe incidence rates, Population and climatic indicators by quarter, RCP and period in All regions in Sweden 2000-2090

				Decade									
	rcp	Quarter	1970-1999	2000	2010	2020	2030	2040	2050	2060	2070	2080	2090
**TBE incidence rate**	rcp26					4.47(0.87)	4.55(0.69)	4.46(0.58)	4.47(0.62)	4.4(0.56)	4.27(0.66)	4.22(0.76)	4.02(0.53)
	rcp45					4.62(0.83)	4.61(0.55)	4.77(0.56)	4.6(0.59)	4.67(0.54)	4.69(0.54)	4.62(0.52)	4.53(0.51)
	rcp85					4.56(0.77)	4.79(0.56)	4.85(0.56)	4.86(0.5)	5.14(0.51)	5.16(0.49)	5.28(0.42)	5.26(0.38)
**Predicted tbe cases**	rcp26					480.56(94.41)	506.43(76.88)	516.75(67.56)	537.28(74.07)	547.88(69.68)	550.37(85.47)	562.54(100.82)	552.05(73.38)
	rcp45					496.24(90.45)	512.91(60.63)	551.89(65.75)	553.64(70.38)	581.79(68.65)	603.83(68.59)	615.79(69.35)	621.72(70.12)
	rcp85					489.6(82.08)	532.03(61.98)	561.61(64.02)	584.39(60.29)	640.64(64.29)	664.89(63.54)	703.82(56.92)	722.3(52.23)
**Population**	rcp26					10.75	11.12	11.58	12.03	12.46	12.89	13.32	13.73
**Mean temperature**	rcp26	Q01	-3.9(2.36)	-3.21(2.79)	-2.63(2.19)	-2.63(2.38)	-2.15(2.16)	-1.11(1.9)	-2.06(1.88)	-1.92(2.39)	-1.65(2.29)	-1.54(2.14)	-1.92(2.02)
	rcp26	Q02	8.04(1.03)	8.88(1.05)	9.02(0.89)	9.25(1.19)	9.31(1.15)	9.53(0.98)	9.69(1.15)	9.53(1.19)	9.46(1.07)	9.73(1.01)	9.39(1.06)
	rcp26	Q03	12.89(0.98)	13.62(1.06)	13.91(0.92)	14.11(0.94)	14.3(1.04)	14.46(0.93)	14.63(1.03)	14.65(1.1)	14.75(1.28)	14.72(1.26)	14.47(1.04)
	rcp26	Q04	0.42(1.32)	1.24(1.26)	1.72(1.15)	1.78(1.31)	2.04(1.21)	2.33(1.1)	2.27(1.15)	2.15(1.34)	2.36(1.27)	2.28(1.14)	1.85(1.34)
	rcp45	Q01	-3.9(2.36)	-3.05(2.71)	-2.62(2.16)	-2.24(2.51)	-1.49(1.87)	-1.33(2.38)	-0.85(1.83)	-0.35(2.1)	-0.58(1.91)	-0.43(2.1)	0.07(2.11)
	rcp45	Q02	8.04(1.03)	8.73(0.99)	9.04(1.08)	9.3(1.1)	9.66(1.21)	9.96(1.11)	10.08(1.14)	10.47(1.37)	10.41(1.12)	10.73(1.22)	10.67(1.09)
	rcp45	Q03	12.89(0.98)	13.65(1.01)	13.98(0.8)	14.32(1.15)	14.45(0.87)	15.03(1.32)	14.79(0.98)	15.38(1.17)	15.59(1.21)	15.59(1.15)	15.71(1.24)
	rcp45	Q04	0.42(1.32)	1.34(1.21)	1(1.49)	1.86(1.03)	2.33(1.19)	2.36(1.29)	2.69(1.27)	3.03(1.16)	3.17(1.53)	3.14(1.34)	3.49(1.19)
	rcp85	Q01	-3.9(2.36)	-2.85(2.75)	-2.39(2.63)	-2.16(2.26)	-1.5(2.19)	-0.93(2.11)	-0.03(1.9)	0.25(1.74)	1.14(1.74)	1.85(1.71)	2.43(1.72)
	rcp85	Q02	8.04(1.03)	8.81(1.02)	9.07(0.96)	9.43(1.03)	9.86(0.97)	10.15(1.17)	10.65(1.26)	11.13(1.2)	11.41(1.17)	12.05(1.39)	12.71(1.4)
	rcp85	Q03	12.89(0.98)	13.61(1.1)	13.96(0.83)	14.27(1.08)	14.82(1.09)	15.15(1.07)	15.53(1.1)	16.51(1.31)	16.95(1.44)	17.86(1.54)	18.09(1.45)
	rcp85	Q04	0.42(1.32)	1.26(1.28)	1.98(1.31)	2.1(1.14)	2.39(1.15)	2.63(1.05)	3.75(1.26)	3.97(1.45)	4.44(1.39)	5.31(1.26)	5.56(1.42)
**Minimum temperature**	rcp26	Q01	-7.3(2.68)	-6.47(3.11)	-5.77(2.45)	-5.78(2.65)	-5.25(2.39)	-4.09(2.11)	-5.11(2.08)	-4.93(2.64)	-4.68(2.54)	-4.53(2.37)	-4.93(2.27)
	rcp26	Q02	3.29(0.89)	4.09(0.84)	4.28(0.76)	4.45(1.04)	4.6(1.02)	4.8(0.84)	4.91(0.97)	4.79(1.01)	4.72(0.94)	4.95(0.88)	4.67(0.9)
	rcp26	Q03	8.61(0.77)	9.35(0.83)	9.55(0.79)	9.75(0.77)	9.96(0.83)	10.09(0.75)	10.23(0.82)	10.24(0.88)	10.24(0.97)	10.29(1.03)	10.06(0.94)
	rcp26	Q04	-2.45(1.46)	-1.58(1.39)	-1.03(1.29)	-0.95(1.45)	-0.64(1.33)	-0.4(1.2)	-0.4(1.24)	-0.54(1.43)	-0.34(1.35)	-0.39(1.24)	-0.89(1.48)
	rcp45	Q01	-7.3(2.68)	-6.23(3.01)	-5.77(2.44)	-5.32(2.84)	-4.49(2.07)	-4.29(2.61)	-3.74(1.99)	-3.23(2.26)	-3.41(2.1)	-3.28(2.29)	-2.68(2.33)
	rcp45	Q02	3.29(0.89)	3.97(0.81)	4.29(0.93)	4.56(0.93)	4.96(1.03)	5.22(0.97)	5.3(0.93)	5.68(1.11)	5.7(1)	5.98(1.04)	5.92(0.92)
	rcp45	Q03	8.61(0.77)	9.38(0.79)	9.61(0.62)	9.93(0.86)	10.12(0.71)	10.61(1.01)	10.5(0.82)	11.01(0.99)	11.21(1.04)	11.15(0.99)	11.32(1.01)
	rcp45	Q04	-2.45(1.46)	-1.48(1.36)	-1.79(1.62)	-0.86(1.1)	-0.3(1.29)	-0.33(1.38)	0.07(1.34)	0.41(1.27)	0.55(1.62)	0.52(1.42)	0.92(1.27)
	rcp85	Q01	-7.3(2.68)	-6.04(3.06)	-5.51(2.96)	-5.21(2.49)	-4.49(2.44)	-3.83(2.3)	-2.87(2.05)	-2.47(1.91)	-1.52(1.88)	-0.76(1.8)	-0.15(1.79)
	rcp85	Q02	3.29(0.89)	4.05(0.83)	4.31(0.81)	4.69(0.86)	5.07(0.82)	5.41(1.02)	5.89(1.04)	6.41(1.03)	6.74(1.02)	7.37(1.18)	7.96(1.16)
	rcp85	Q03	8.61(0.77)	9.34(0.87)	9.59(0.68)	9.98(0.83)	10.42(0.93)	10.81(0.84)	11.23(0.86)	12.09(1.06)	12.56(1.16)	13.38(1.25)	13.69(1.25)
	rcp85	Q04	-2.45(1.46)	-1.55(1.44)	-0.75(1.42)	-0.6(1.25)	-0.27(1.25)	0.01(1.09)	1.17(1.33)	1.41(1.51)	1.95(1.42)	2.82(1.28)	3.1(1.45)
**Maximun temperature**	rcp26	Q01	-0.62(2.09)	-0.03(2.56)	0.44(1.96)	0.48(2.16)	0.91(2.02)	1.85(1.74)	0.97(1.72)	1.06(2.17)	1.38(2.1)	1.43(1.96)	1.07(1.87)
	rcp26	Q02	12.96(1.23)	13.83(1.32)	13.92(1.07)	14.19(1.42)	14.19(1.35)	14.43(1.18)	14.64(1.4)	14.41(1.42)	14.35(1.26)	14.67(1.2)	14.27(1.29)
	rcp26	Q03	17.42(1.22)	18.09(1.31)	18.42(1.1)	18.62(1.17)	18.8(1.3)	18.97(1.18)	19.18(1.29)	19.19(1.33)	19.38(1.62)	19.28(1.52)	19.01(1.2)
	rcp26	Q04	3.15(1.24)	3.96(1.19)	4.39(1.06)	4.45(1.24)	4.68(1.16)	5(1.06)	4.89(1.12)	4.79(1.31)	5.03(1.23)	4.93(1.1)	4.53(1.27)
	rcp45	Q01	-0.62(2.09)	0.09(2.49)	0.44(1.97)	0.79(2.24)	1.46(1.73)	1.59(2.19)	2.02(1.74)	2.57(2)	2.26(1.79)	2.44(1.96)	2.85(1.97)
	rcp45	Q02	12.96(1.23)	13.65(1.25)	13.94(1.3)	14.21(1.32)	14.55(1.43)	14.88(1.31)	15.03(1.41)	15.44(1.67)	15.31(1.31)	15.66(1.47)	15.61(1.3)
	rcp45	Q03	17.42(1.22)	18.12(1.28)	18.55(1.07)	18.86(1.44)	18.91(1.06)	19.55(1.61)	19.22(1.2)	19.87(1.4)	20.08(1.4)	20.1(1.35)	20.2(1.46)
	rcp45	Q04	3.15(1.24)	4.07(1.12)	3.71(1.41)	4.53(1.01)	4.91(1.16)	5.01(1.26)	5.28(1.25)	5.64(1.12)	5.79(1.51)	5.74(1.3)	6.05(1.15)
	rcp85	Q01	-0.62(2.09)	0.27(2.53)	0.65(2.37)	0.84(2.11)	1.47(2.01)	1.95(1.97)	2.83(1.82)	3.05(1.66)	3.88(1.67)	4.56(1.71)	5.11(1.72)
	rcp85	Q02	12.96(1.23)	13.73(1.26)	13.99(1.18)	14.34(1.25)	14.8(1.19)	15.07(1.39)	15.58(1.53)	16.05(1.44)	16.27(1.37)	16.92(1.64)	17.62(1.66)
	rcp85	Q03	17.42(1.22)	18.1(1.36)	18.48(1.07)	18.73(1.33)	19.34(1.29)	19.61(1.32)	19.95(1.37)	21.02(1.56)	21.4(1.7)	22.33(1.78)	22.48(1.67)
	rcp85	Q04	3.15(1.24)	3.99(1.18)	4.65(1.27)	4.75(1.09)	5.02(1.1)	5.23(1.06)	6.33(1.24)	6.54(1.44)	6.97(1.4)	7.83(1.27)	8.06(1.4)

Table S11: Ensemble Mean of TBE cases,Tbe incidence rates, Population and climatic indicators by quarter, RCP and period in Stockholm in Sweden 2000-2090

				Decade									
	rcp	Quarter	1970-1999	2000	2010	2020	2030	2040	2050	2060	2070	2080	2090
**TBE incidence rate**	rcp26					6.61(1.19)	6.62(0.9)	6.42(0.76)	6.35(0.83)	6.22(0.71)	6.03(0.86)	5.95(1.01)	5.67(0.68)
	rcp45					6.8(1.11)	6.69(0.7)	6.82(0.74)	6.5(0.81)	6.59(0.74)	6.56(0.74)	6.46(0.7)	6.33(0.68)
	rcp85					6.73(1.02)	6.93(0.75)	6.93(0.74)	6.86(0.7)	7.17(0.69)	7.15(0.66)	7.27(0.54)	7.21(0.48)
**Predicted tbe cases**	rcp26					287.31(52.07)	302.21(41.21)	309.67(36.59)	321.47(41.31)	327.71(36.7)	329.34(47.24)	336.64(56.92)	330.9(39.76)
	rcp45					295.22(48.64)	305.12(31.26)	329.1(35.95)	329.12(40.68)	346.95(39.73)	358.2(39.79)	365.65(40.16)	369.71(40)
	rcp85					292.02(43.82)	315.86(33.61)	334.36(34.81)	347.42(35.33)	377.48(36.75)	390.7(36.21)	411.47(31.16)	421.19(28.54)
**Population**	rcp26					4.34	4.56	4.82	5.06	5.27	5.46	5.66	5.84
**Mean temperature**	rcp26	Q01	-2.6(2.7)	-1.89(3.01)	-1.32(2.4)	-1.31(2.63)	-0.78(2.39)	0.35(2.2)	-0.68(2.07)	-0.55(2.68)	-0.2(2.51)	-0.11(2.36)	-0.51(2.16)
	rcp26	Q02	9.25(1.13)	10.06(1.17)	10.26(0.97)	10.45(1.26)	10.52(1.22)	10.81(1.06)	10.95(1.2)	10.77(1.25)	10.71(1.12)	11.02(1.07)	10.62(1.09)
	rcp26	Q03	14.2(1.07)	14.91(1.18)	15.21(0.98)	15.45(1.05)	15.67(1.13)	15.82(1.03)	15.98(1.14)	15.99(1.18)	16.12(1.39)	16.12(1.39)	15.78(1.11)
	rcp26	Q04	1.9(1.35)	2.63(1.29)	3.08(1.17)	3.16(1.33)	3.45(1.17)	3.67(1.12)	3.66(1.17)	3.5(1.4)	3.71(1.3)	3.64(1.13)	3.19(1.38)
	rcp45	Q01	-2.6(2.7)	-1.69(2.96)	-1.3(2.39)	-0.87(2.81)	0.01(2.06)	0.12(2.59)	0.65(1.96)	1.13(2.23)	0.8(2.06)	1(2.29)	1.58(2.28)
	rcp45	Q02	9.25(1.13)	9.93(1.11)	10.25(1.11)	10.53(1.15)	10.94(1.22)	11.24(1.22)	11.32(1.19)	11.75(1.43)	11.63(1.16)	11.99(1.27)	11.97(1.13)
	rcp45	Q03	14.2(1.07)	14.98(1.13)	15.3(0.93)	15.65(1.27)	15.76(0.93)	16.43(1.42)	16.11(1.07)	16.79(1.31)	16.91(1.28)	16.94(1.24)	17.08(1.33)
	rcp45	Q04	1.9(1.35)	2.73(1.28)	2.4(1.56)	3.3(1.01)	3.77(1.19)	3.72(1.34)	4.06(1.29)	4.41(1.17)	4.48(1.54)	4.45(1.37)	4.83(1.2)
	rcp85	Q01	-2.6(2.7)	-1.47(2.96)	-0.98(2.79)	-0.75(2.44)	-0.07(2.36)	0.47(2.29)	1.48(1.99)	1.73(1.88)	2.63(1.85)	3.33(1.78)	3.88(1.85)
	rcp85	Q02	9.25(1.13)	10.03(1.12)	10.29(1.02)	10.67(1.08)	11.1(1.03)	11.4(1.21)	11.87(1.31)	12.38(1.23)	12.6(1.21)	13.28(1.4)	13.91(1.44)
	rcp85	Q03	14.2(1.07)	14.93(1.21)	15.27(0.93)	15.61(1.19)	16.2(1.19)	16.51(1.2)	16.93(1.23)	17.88(1.46)	18.32(1.52)	19.29(1.65)	19.54(1.57)
	rcp85	Q04	1.9(1.35)	2.68(1.33)	3.36(1.35)	3.46(1.17)	3.75(1.19)	4(1.04)	5.07(1.23)	5.27(1.45)	5.72(1.42)	6.53(1.24)	6.78(1.42)
**Minimum temperature**	rcp26	Q01	-5.77(3.12)	-4.94(3.38)	-4.22(2.71)	-4.24(2.94)	-3.65(2.63)	-2.4(2.45)	-3.5(2.3)	-3.36(2.98)	-2.99(2.79)	-2.86(2.61)	-3.27(2.43)
	rcp26	Q02	4.38(0.96)	5.14(0.93)	5.39(0.82)	5.54(1.07)	5.7(1.03)	5.95(0.89)	6.04(0.99)	5.9(1.05)	5.84(0.97)	6.1(0.93)	5.77(0.9)
	rcp26	Q03	9.76(0.84)	10.51(0.92)	10.71(0.83)	10.96(0.84)	11.19(0.87)	11.31(0.81)	11.45(0.89)	11.46(0.94)	11.46(1.03)	11.54(1.09)	11.24(0.98)
	rcp26	Q04	-0.85(1.5)	-0.08(1.42)	0.41(1.31)	0.51(1.46)	0.85(1.28)	1.02(1.21)	1.07(1.25)	0.88(1.48)	1.06(1.37)	1.03(1.23)	0.51(1.51)
	rcp45	Q01	-5.77(3.12)	-4.64(3.29)	-4.2(2.71)	-3.71(3.17)	-2.75(2.27)	-2.61(2.83)	-2(2.11)	-1.55(2.4)	-1.84(2.24)	-1.66(2.5)	-0.99(2.51)
	rcp45	Q02	4.38(0.96)	5.05(0.9)	5.39(0.95)	5.66(0.94)	6.09(1.03)	6.37(1.06)	6.39(0.92)	6.82(1.15)	6.78(1)	7.1(1.07)	7.08(0.97)
	rcp45	Q03	9.76(0.84)	10.59(0.87)	10.78(0.69)	11.12(0.94)	11.31(0.75)	11.88(1.07)	11.7(0.88)	12.3(1.09)	12.43(1.09)	12.39(1.05)	12.56(1.06)
	rcp45	Q04	-0.85(1.5)	0(1.45)	-0.31(1.71)	0.66(1.08)	1.2(1.27)	1.1(1.42)	1.5(1.34)	1.82(1.27)	1.9(1.61)	1.86(1.42)	2.29(1.26)
	rcp85	Q01	-5.77(3.12)	-4.42(3.32)	-3.88(3.15)	-3.58(2.68)	-2.84(2.61)	-2.23(2.48)	-1.15(2.15)	-0.82(2.04)	0.1(1.98)	0.81(1.82)	1.34(1.91)
	rcp85	Q02	4.38(0.96)	5.15(0.91)	5.4(0.85)	5.8(0.89)	6.18(0.87)	6.51(1.01)	6.98(1.06)	7.53(1.04)	7.82(1.02)	8.47(1.15)	9.03(1.16)
	rcp85	Q03	9.76(0.84)	10.53(0.95)	10.78(0.74)	11.17(0.88)	11.66(0.98)	12.05(0.93)	12.51(0.94)	13.34(1.15)	13.84(1.18)	14.69(1.3)	15.04(1.32)
	rcp85	Q04	-0.85(1.5)	-0.04(1.49)	0.7(1.45)	0.84(1.25)	1.14(1.27)	1.44(1.08)	2.53(1.28)	2.72(1.47)	3.22(1.38)	4(1.2)	4.26(1.4)
**Maximun temperature**	rcp26	Q01	0.49(2.39)	1.12(2.77)	1.58(2.17)	1.64(2.42)	2.13(2.28)	3.15(2.06)	2.18(1.94)	2.28(2.46)	2.67(2.34)	2.7(2.23)	2.29(2.04)
	rcp26	Q02	14.39(1.36)	15.24(1.44)	15.35(1.18)	15.59(1.52)	15.58(1.46)	15.9(1.29)	16.09(1.49)	15.85(1.5)	15.79(1.33)	16.16(1.29)	15.69(1.36)
	rcp26	Q03	18.87(1.35)	19.5(1.45)	19.86(1.23)	20.08(1.35)	20.31(1.46)	20.46(1.35)	20.65(1.44)	20.65(1.45)	20.9(1.8)	20.8(1.71)	20.43(1.31)
	rcp26	Q04	4.51(1.28)	5.25(1.23)	5.66(1.09)	5.74(1.28)	5.99(1.15)	6.27(1.09)	6.2(1.15)	6.07(1.39)	6.31(1.28)	6.2(1.09)	5.79(1.31)
	rcp45	Q01	0.49(2.39)	1.27(2.73)	1.6(2.2)	2(2.55)	2.81(1.95)	2.89(2.45)	3.38(1.92)	3.94(2.19)	3.52(1.99)	3.76(2.2)	4.25(2.16)
	rcp45	Q02	14.39(1.36)	15.07(1.38)	15.35(1.34)	15.64(1.39)	16.03(1.47)	16.36(1.44)	16.46(1.5)	16.91(1.75)	16.71(1.37)	17.13(1.53)	17.09(1.35)
	rcp45	Q03	18.87(1.35)	19.56(1.43)	19.98(1.27)	20.31(1.6)	20.34(1.15)	21.09(1.75)	20.66(1.33)	21.43(1.6)	21.51(1.53)	21.57(1.52)	21.71(1.6)
	rcp45	Q04	4.51(1.28)	5.37(1.19)	5.02(1.47)	5.87(1)	6.27(1.17)	6.29(1.31)	6.58(1.28)	6.97(1.16)	7.04(1.55)	7(1.35)	7.34(1.19)
	rcp85	Q01	0.49(2.39)	1.48(2.74)	1.89(2.57)	2.1(2.33)	2.75(2.22)	3.23(2.22)	4.23(1.97)	4.43(1.83)	5.31(1.84)	6.01(1.85)	6.57(1.87)
	rcp85	Q02	14.39(1.36)	15.16(1.38)	15.41(1.22)	15.79(1.31)	16.23(1.27)	16.52(1.45)	16.99(1.59)	17.48(1.49)	17.61(1.44)	18.31(1.68)	18.99(1.73)
	rcp85	Q03	18.87(1.35)	19.53(1.49)	19.92(1.22)	20.2(1.48)	20.85(1.46)	21.1(1.48)	21.47(1.55)	22.49(1.77)	22.88(1.86)	23.86(1.95)	24.01(1.86)
	rcp85	Q04	4.51(1.28)	5.31(1.24)	5.95(1.32)	6.04(1.14)	6.3(1.16)	6.53(1.06)	7.61(1.23)	7.82(1.47)	8.24(1.47)	9.06(1.3)	9.3(1.43)

Table S12: Ensemble Mean of TBE cases,Tbe incidence rates, Population and climatic indicators by quarter, RCP and period in West Sweden in Sweden 2000-2090

				Decade									
	rcp	Quarter	1970-1999	2000	2010	2020	2030	2040	2050	2060	2070	2080	2090
**TBE incidence rate**	rcp26					4.93(1.01)	4.98(0.79)	4.87(0.65)	4.86(0.67)	4.77(0.65)	4.64(0.72)	4.57(0.81)	4.36(0.6)
	rcp45					5.09(0.95)	5.08(0.65)	5.15(0.63)	5.03(0.62)	5.04(0.56)	5.07(0.56)	5(0.56)	4.86(0.54)
	rcp85					5.03(0.88)	5.23(0.62)	5.26(0.64)	5.23(0.54)	5.54(0.54)	5.56(0.52)	5.68(0.45)	5.66(0.4)
**Predicted tbe cases**	rcp26					106.01(21.81)	111.32(17.6)	113.5(15.22)	117.83(16.01)	119.96(16.43)	120.78(18.59)	122.87(21.66)	121.13(16.57)
	rcp45					109.51(20.61)	113.45(14.35)	120.18(14.69)	122.1(14.89)	126.65(14.23)	131.94(14.34)	134.68(15.06)	134.84(14.97)
	rcp85					108.14(18.8)	116.87(13.74)	122.51(14.59)	126.86(13.1)	139.39(13.72)	144.73(13.49)	152.89(12.32)	157.05(11.27)
**Population**	rcp26					2.15	2.23	2.33	2.43	2.51	2.6	2.69	2.78
**Mean temperature**	rcp26	Q01	-1.37(2.42)	-0.83(2.83)	-0.42(2.17)	-0.41(2.42)	0.07(2.25)	1.08(1.93)	0.09(1.9)	0.25(2.48)	0.59(2.28)	0.65(2.22)	0.25(2.06)
	rcp26	Q02	9.52(1.07)	10.26(1.1)	10.41(0.96)	10.59(1.25)	10.66(1.2)	10.86(1.02)	11.04(1.16)	10.9(1.19)	10.77(1.07)	11.09(1.02)	10.72(1.03)
	rcp26	Q03	14.11(1.04)	14.87(1.15)	15.12(0.93)	15.29(1.02)	15.49(1.08)	15.67(1)	15.82(1.08)	15.9(1.2)	16(1.34)	15.96(1.31)	15.67(1.07)
	rcp26	Q04	2.93(1.32)	3.69(1.28)	4.03(1.1)	4.07(1.3)	4.33(1.2)	4.61(1.13)	4.53(1.21)	4.41(1.38)	4.63(1.31)	4.49(1.15)	4.1(1.36)
	rcp45	Q01	-1.37(2.42)	-0.7(2.81)	-0.3(2.25)	-0.08(2.66)	0.8(2.02)	0.84(2.43)	1.36(1.93)	1.78(2.16)	1.46(1.98)	1.65(2.16)	2.06(2.09)
	rcp45	Q02	9.52(1.07)	10.16(1.02)	10.44(1.16)	10.68(1.14)	10.99(1.2)	11.23(1.16)	11.4(1.18)	11.79(1.45)	11.71(1.18)	11.99(1.24)	11.9(1.1)
	rcp45	Q03	14.11(1.04)	14.9(1.1)	15.24(0.88)	15.5(1.22)	15.64(0.97)	16.23(1.41)	16.01(1.08)	16.59(1.22)	16.79(1.25)	16.8(1.24)	16.9(1.26)
	rcp45	Q04	2.93(1.32)	3.77(1.23)	3.33(1.58)	4.21(1.04)	4.6(1.23)	4.61(1.35)	4.9(1.31)	5.25(1.17)	5.3(1.59)	5.36(1.37)	5.62(1.25)
	rcp85	Q01	-1.37(2.42)	-0.48(2.82)	-0.07(2.57)	0.02(2.45)	0.65(2.25)	1.17(2.17)	2.12(1.98)	2.22(1.81)	3.11(1.82)	3.87(1.74)	4.33(1.8)
	rcp85	Q02	9.52(1.07)	10.23(1.07)	10.47(1)	10.74(1.07)	11.21(0.99)	11.42(1.25)	11.97(1.31)	12.38(1.24)	12.57(1.18)	13.25(1.44)	13.82(1.43)
	rcp85	Q03	14.11(1.04)	14.86(1.19)	15.2(0.89)	15.45(1.16)	16.04(1.18)	16.31(1.16)	16.69(1.2)	17.73(1.36)	18.13(1.45)	19.02(1.56)	19.22(1.45)
	rcp85	Q04	2.93(1.32)	3.68(1.28)	4.29(1.34)	4.37(1.15)	4.58(1.16)	4.81(1.08)	5.89(1.31)	6.1(1.48)	6.49(1.43)	7.33(1.28)	7.52(1.42)
**Minimum temperature**	rcp26	Q01	-4.22(2.84)	-3.58(3.26)	-3.06(2.5)	-3.09(2.73)	-2.54(2.54)	-1.43(2.15)	-2.5(2.11)	-2.29(2.78)	-2(2.59)	-1.85(2.44)	-2.29(2.32)
	rcp26	Q02	5.05(0.9)	5.73(0.86)	5.91(0.79)	6.07(1.09)	6.2(1.03)	6.38(0.85)	6.5(0.94)	6.4(0.98)	6.29(0.9)	6.52(0.87)	6.24(0.83)
	rcp26	Q03	10(0.79)	10.72(0.89)	10.87(0.77)	11.03(0.81)	11.27(0.84)	11.38(0.76)	11.51(0.81)	11.58(0.91)	11.55(0.99)	11.6(1.04)	11.35(0.89)
	rcp26	Q04	0.35(1.47)	1.13(1.41)	1.53(1.23)	1.57(1.46)	1.89(1.3)	2.11(1.24)	2.09(1.31)	1.94(1.48)	2.17(1.42)	2.04(1.24)	1.58(1.52)
	rcp45	Q01	-4.22(2.84)	-3.38(3.22)	-2.94(2.58)	-2.71(3.07)	-1.69(2.25)	-1.67(2.69)	-1.08(2.07)	-0.66(2.33)	-0.96(2.18)	-0.77(2.35)	-0.25(2.3)
	rcp45	Q02	5.05(0.9)	5.66(0.82)	5.94(0.95)	6.18(0.94)	6.55(1.02)	6.73(0.97)	6.84(0.93)	7.19(1.13)	7.19(1.02)	7.45(1)	7.34(0.89)
	rcp45	Q03	10(0.79)	10.74(0.84)	10.95(0.65)	11.19(0.9)	11.37(0.74)	11.9(1.06)	11.77(0.85)	12.26(1)	12.42(1.05)	12.39(1.02)	12.56(1.01)
	rcp45	Q04	0.35(1.47)	1.22(1.4)	0.77(1.78)	1.74(1.11)	2.22(1.35)	2.15(1.45)	2.5(1.38)	2.86(1.28)	2.86(1.68)	2.94(1.48)	3.28(1.34)
	rcp85	Q01	-4.22(2.84)	-3.16(3.25)	-2.68(2.95)	-2.57(2.72)	-1.85(2.49)	-1.3(2.37)	-0.28(2.15)	-0.12(1.96)	0.83(1.94)	1.61(1.84)	2.04(1.91)
	rcp85	Q02	5.05(0.9)	5.73(0.88)	5.98(0.82)	6.26(0.86)	6.63(0.81)	6.9(1.06)	7.39(1.03)	7.85(1.03)	8.09(1)	8.72(1.18)	9.24(1.1)
	rcp85	Q03	10(0.79)	10.69(0.92)	10.93(0.7)	11.26(0.85)	11.72(0.97)	12.03(0.87)	12.43(0.9)	13.27(1.06)	13.71(1.13)	14.51(1.26)	14.75(1.23)
	rcp85	Q04	0.35(1.47)	1.12(1.46)	1.82(1.46)	1.91(1.25)	2.15(1.26)	2.41(1.11)	3.52(1.38)	3.75(1.55)	4.2(1.46)	5.03(1.31)	5.26(1.47)
**Maximun temperature**	rcp26	Q01	1.36(2.15)	1.85(2.59)	2.2(1.95)	2.25(2.23)	2.7(2.11)	3.6(1.8)	2.71(1.78)	2.8(2.28)	3.21(2.11)	3.21(2.08)	2.84(1.93)
	rcp26	Q02	14.2(1.29)	14.97(1.39)	15.09(1.18)	15.28(1.49)	15.31(1.43)	15.51(1.24)	15.77(1.43)	15.56(1.45)	15.42(1.29)	15.81(1.27)	15.36(1.31)
	rcp26	Q03	18.38(1.31)	19.11(1.45)	19.38(1.14)	19.58(1.29)	19.75(1.37)	19.95(1.3)	20.14(1.37)	20.22(1.52)	20.44(1.74)	20.33(1.58)	20(1.31)
	rcp26	Q04	5.38(1.25)	6.15(1.21)	6.45(1.01)	6.51(1.22)	6.72(1.18)	7.05(1.07)	6.92(1.16)	6.82(1.33)	7.05(1.25)	6.91(1.12)	6.56(1.27)
	rcp45	Q01	1.36(2.15)	1.95(2.55)	2.3(2.07)	2.52(2.37)	3.31(1.89)	3.36(2.25)	3.83(1.88)	4.34(2.11)	3.95(1.87)	4.16(2.04)	4.48(1.97)
	rcp45	Q02	14.2(1.29)	14.84(1.28)	15.12(1.43)	15.36(1.39)	15.62(1.44)	15.9(1.39)	16.12(1.49)	16.54(1.8)	16.39(1.4)	16.68(1.53)	16.6(1.35)
	rcp45	Q03	18.38(1.31)	19.15(1.42)	19.61(1.19)	19.83(1.56)	19.91(1.21)	20.53(1.75)	20.25(1.36)	20.89(1.5)	21.12(1.49)	21.14(1.53)	21.19(1.52)
	rcp45	Q04	5.38(1.25)	6.24(1.14)	5.81(1.47)	6.63(1.02)	6.93(1.18)	7.04(1.3)	7.28(1.28)	7.61(1.13)	7.71(1.55)	7.74(1.32)	7.95(1.19)
	rcp85	Q01	1.36(2.15)	2.16(2.58)	2.5(2.34)	2.61(2.29)	3.21(2.09)	3.67(2.05)	4.6(1.93)	4.71(1.76)	5.54(1.75)	6.28(1.72)	6.74(1.77)
	rcp85	Q02	14.2(1.29)	14.91(1.31)	15.16(1.25)	15.41(1.34)	15.94(1.23)	16.1(1.48)	16.68(1.63)	17.07(1.52)	17.2(1.41)	17.89(1.74)	18.5(1.74)
	rcp85	Q03	18.38(1.31)	19.13(1.5)	19.5(1.17)	19.68(1.46)	20.34(1.45)	20.57(1.45)	20.92(1.52)	22.08(1.68)	22.42(1.78)	23.35(1.84)	23.46(1.73)
	rcp85	Q04	5.38(1.25)	6.14(1.18)	6.69(1.29)	6.78(1.11)	6.99(1.12)	7.19(1.09)	8.24(1.27)	8.45(1.44)	8.79(1.4)	9.63(1.25)	9.78(1.37)

Table S13: Ensemble Mean of TBE cases,Tbe incidence rates, Population and climatic indicators by quarter, RCP and period in North Middle Sweden in Sweden 2000-2090

				Decade									
	rcp	Quarter	1970-1999	2000	2010	2020	2030	2040	2050	2060	2070	2080	2090
**TBE incidence rate**	rcp26					4.76(1.36)	5.11(1.21)	5.13(1.05)	5.3(1.09)	5.29(1.04)	5.14(1.2)	5.12(1.24)	4.83(1.03)
	rcp45					5.04(1.36)	5.19(1.03)	5.62(1)	5.52(1.01)	5.67(0.89)	5.79(0.88)	5.71(0.85)	5.63(0.86)
	rcp85					4.95(1.25)	5.5(0.95)	5.73(0.98)	5.93(0.78)	6.48(0.84)	6.54(0.82)	6.8(0.77)	6.86(0.69)
**Predicted tbe cases**	rcp26					40.9(11.71)	43.73(10.39)	43.88(8.96)	46(9.42)	46.88(9.24)	46.86(10.93)	48.15(11.6)	46.62(9.95)
	rcp45					43.22(11.63)	44.41(8.81)	48.1(8.6)	47.93(8.72)	50.28(7.95)	52.78(8.01)	53.63(8.02)	54.37(8.26)
	rcp85					42.47(10.7)	47(8.11)	49.08(8.33)	51.49(6.76)	57.46(7.46)	59.67(7.55)	63.91(7.34)	66.26(6.74)
**Population**	rcp26					0.86	0.86	0.86	0.87	0.89	0.91	0.94	0.97
**Mean temperature**	rcp26	Q01	-5.03(2.54)	-4.34(3.07)	-3.68(2.41)	-3.72(2.6)	-3.2(2.37)	-2.05(2.12)	-3.13(2.16)	-2.99(2.62)	-2.76(2.52)	-2.57(2.32)	-2.96(2.23)
	rcp26	Q02	7.62(1.1)	8.45(1.12)	8.57(0.92)	8.8(1.24)	8.87(1.2)	9.08(1.04)	9.25(1.21)	9.06(1.27)	9(1.09)	9.3(1.04)	8.88(1.14)
	rcp26	Q03	12.26(1.01)	12.95(1.09)	13.23(0.95)	13.39(0.95)	13.6(1.06)	13.75(0.96)	13.94(1.08)	13.96(1.12)	14.07(1.34)	14.04(1.29)	13.77(1.07)
	rcp26	Q04	-0.73(1.45)	0.07(1.41)	0.57(1.32)	0.6(1.44)	0.81(1.33)	1.16(1.19)	1.1(1.28)	0.98(1.42)	1.18(1.38)	1.11(1.23)	0.66(1.45)
	rcp45	Q01	-5.03(2.54)	-4.16(2.97)	-3.65(2.41)	-3.26(2.7)	-2.6(2.07)	-2.32(2.57)	-1.88(2.03)	-1.32(2.3)	-1.54(2.05)	-1.44(2.31)	-0.8(2.38)
	rcp45	Q02	7.62(1.1)	8.29(1.09)	8.62(1.13)	8.85(1.14)	9.18(1.28)	9.5(1.17)	9.64(1.2)	10.07(1.46)	9.94(1.16)	10.29(1.28)	10.27(1.19)
	rcp45	Q03	12.26(1.01)	12.98(1.05)	13.3(0.84)	13.63(1.15)	13.76(0.92)	14.33(1.37)	14.08(1.03)	14.63(1.18)	14.92(1.26)	14.86(1.18)	14.98(1.28)
	rcp45	Q04	-0.73(1.45)	0.17(1.36)	-0.19(1.67)	0.69(1.13)	1.18(1.28)	1.22(1.32)	1.55(1.35)	1.86(1.27)	1.99(1.62)	1.96(1.45)	2.36(1.24)
	rcp85	Q01	-5.03(2.54)	-3.99(3.01)	-3.47(2.99)	-3.21(2.41)	-2.57(2.5)	-1.89(2.36)	-0.97(2.05)	-0.69(1.92)	0.3(1.95)	0.91(1.94)	1.66(1.89)
	rcp85	Q02	7.62(1.1)	8.35(1.11)	8.63(1)	8.99(1.09)	9.38(1.05)	9.73(1.21)	10.21(1.35)	10.66(1.25)	10.98(1.28)	11.62(1.52)	12.31(1.54)
	rcp85	Q03	12.26(1.01)	12.95(1.14)	13.24(0.85)	13.61(1.11)	14.1(1.08)	14.45(1.13)	14.83(1.14)	15.83(1.4)	16.24(1.55)	17.12(1.64)	17.38(1.57)
	rcp85	Q04	-0.73(1.45)	0.09(1.44)	0.85(1.41)	0.98(1.25)	1.25(1.28)	1.47(1.15)	2.62(1.36)	2.77(1.55)	3.22(1.45)	4.1(1.31)	4.33(1.43)
**Minimum temperature**	rcp26	Q01	-8.95(2.92)	-8.06(3.46)	-7.25(2.75)	-7.27(2.95)	-6.7(2.75)	-5.37(2.45)	-6.57(2.51)	-6.4(2.98)	-6.15(2.87)	-5.92(2.74)	-6.36(2.6)
	rcp26	Q02	2.55(0.95)	3.33(0.9)	3.51(0.78)	3.67(1.07)	3.86(1.06)	4.01(0.9)	4.15(1.03)	3.99(1.09)	3.94(0.98)	4.2(0.91)	3.86(0.95)
	rcp26	Q03	7.7(0.81)	8.41(0.87)	8.61(0.85)	8.8(0.84)	9.02(0.93)	9.12(0.83)	9.29(0.9)	9.33(0.96)	9.31(1.07)	9.37(1.08)	9.11(1.02)
	rcp26	Q04	-3.86(1.64)	-2.98(1.58)	-2.45(1.51)	-2.37(1.6)	-2.14(1.49)	-1.8(1.31)	-1.81(1.4)	-1.95(1.53)	-1.78(1.49)	-1.78(1.35)	-2.33(1.62)
	rcp45	Q01	-8.95(2.92)	-7.8(3.3)	-7.23(2.82)	-6.72(3.12)	-6.02(2.38)	-5.61(2.91)	-5.1(2.33)	-4.51(2.61)	-4.67(2.34)	-4.61(2.62)	-3.85(2.71)
	rcp45	Q02	2.55(0.95)	3.2(0.89)	3.55(0.98)	3.79(0.96)	4.16(1.09)	4.46(1.02)	4.53(0.97)	4.95(1.19)	4.92(1.05)	5.22(1.11)	5.2(1.01)
	rcp45	Q03	7.7(0.81)	8.47(0.84)	8.68(0.7)	9.01(0.92)	9.21(0.79)	9.67(1.11)	9.57(0.89)	10.09(1.08)	10.34(1.16)	10.23(1.1)	10.39(1.1)
	rcp45	Q04	-3.86(1.64)	-2.89(1.55)	-3.2(1.81)	-2.27(1.28)	-1.7(1.39)	-1.73(1.44)	-1.3(1.47)	-1.02(1.41)	-0.87(1.76)	-0.9(1.57)	-0.44(1.35)
	rcp85	Q01	-8.95(2.92)	-7.62(3.39)	-7.01(3.47)	-6.66(2.74)	-5.96(2.91)	-5.09(2.67)	-4.12(2.31)	-3.66(2.17)	-2.58(2.19)	-1.89(2.13)	-1.05(2.01)
	rcp85	Q02	2.55(0.95)	3.27(0.9)	3.56(0.85)	3.92(0.9)	4.28(0.89)	4.65(1.05)	5.14(1.1)	5.6(1.08)	5.97(1.09)	6.61(1.28)	7.19(1.27)
	rcp85	Q03	7.7(0.81)	8.41(0.92)	8.63(0.75)	9.07(0.9)	9.48(0.99)	9.87(0.92)	10.32(0.96)	11.22(1.21)	11.7(1.36)	12.54(1.41)	12.87(1.42)
	rcp85	Q04	-3.86(1.64)	-2.97(1.63)	-2.14(1.55)	-1.96(1.39)	-1.67(1.41)	-1.41(1.23)	-0.18(1.46)	-0.02(1.65)	0.49(1.5)	1.38(1.35)	1.63(1.49)
**Maximun temperature**	rcp26	Q01	-1.3(2.19)	-0.77(2.73)	-0.24(2.07)	-0.25(2.28)	0.21(2.12)	1.19(1.83)	0.25(1.87)	0.33(2.29)	0.58(2.24)	0.7(2.01)	0.36(1.98)
	rcp26	Q02	12.82(1.33)	13.68(1.43)	13.76(1.15)	14.03(1.5)	14.01(1.41)	14.28(1.26)	14.48(1.49)	14.23(1.5)	14.18(1.31)	14.5(1.25)	14.03(1.42)
	rcp26	Q03	17.08(1.26)	17.73(1.36)	18.04(1.13)	18.16(1.17)	18.39(1.31)	18.57(1.19)	18.79(1.35)	18.78(1.35)	18.99(1.69)	18.87(1.55)	18.6(1.24)
	rcp26	Q04	2.19(1.36)	2.99(1.33)	3.45(1.22)	3.46(1.38)	3.67(1.26)	4.04(1.16)	3.93(1.24)	3.84(1.39)	4.07(1.34)	3.95(1.18)	3.56(1.38)
	rcp45	Q01	-1.3(2.19)	-0.64(2.68)	-0.24(2.08)	0.09(2.31)	0.69(1.81)	0.89(2.28)	1.27(1.85)	1.85(2.11)	1.55(1.86)	1.7(2.09)	2.21(2.14)
	rcp45	Q02	12.82(1.33)	13.49(1.4)	13.8(1.39)	14.04(1.39)	14.34(1.55)	14.66(1.4)	14.89(1.51)	15.33(1.78)	15.13(1.38)	15.52(1.55)	15.5(1.4)
	rcp45	Q03	17.08(1.26)	17.74(1.34)	18.16(1.14)	18.44(1.45)	18.49(1.11)	19.13(1.67)	18.8(1.25)	19.37(1.41)	19.69(1.43)	19.64(1.39)	19.74(1.49)
	rcp45	Q04	2.19(1.36)	3.1(1.25)	2.7(1.58)	3.54(1.09)	3.95(1.26)	4.07(1.29)	4.34(1.32)	4.7(1.25)	4.83(1.59)	4.76(1.41)	5.15(1.21)
	rcp85	Q01	-1.3(2.19)	-0.47(2.68)	-0.07(2.56)	0.11(2.17)	0.74(2.17)	1.26(2.11)	2.14(1.87)	2.33(1.77)	3.23(1.81)	3.84(1.92)	4.53(1.92)
	rcp85	Q02	12.82(1.33)	13.55(1.4)	13.82(1.25)	14.19(1.33)	14.59(1.29)	14.94(1.44)	15.43(1.66)	15.88(1.51)	16.12(1.49)	16.76(1.79)	17.52(1.79)
	rcp85	Q03	17.08(1.26)	17.73(1.41)	18.06(1.1)	18.36(1.37)	18.9(1.28)	19.21(1.38)	19.53(1.4)	20.61(1.62)	20.93(1.77)	21.81(1.8)	22(1.72)
	rcp85	Q04	2.19(1.36)	3.01(1.33)	3.72(1.36)	3.83(1.2)	4.09(1.24)	4.27(1.15)	5.42(1.35)	5.57(1.53)	6(1.47)	6.87(1.33)	7.08(1.43)

Table S14: Ensemble Mean of TBE cases,Tbe incidence rates, Population and climatic indicators by quarter, RCP and period in South Sweden in Sweden 2000-2090

				Decade									
	rcp	Quarter	1970-1999	2000	2010	2020	2030	2040	2050	2060	2070	2080	2090
**TBE incidence rate**	rcp26					1.74(0.39)	1.77(0.33)	1.73(0.28)	1.74(0.29)	1.72(0.27)	1.67(0.31)	1.66(0.35)	1.57(0.27)
	rcp45					1.79(0.39)	1.8(0.29)	1.87(0.26)	1.81(0.25)	1.85(0.25)	1.85(0.22)	1.82(0.22)	1.8(0.22)
	rcp85					1.75(0.37)	1.87(0.29)	1.9(0.26)	1.91(0.23)	2.04(0.21)	2.06(0.2)	2.13(0.17)	2.12(0.16)
**Predicted tbe cases**	rcp26					43.52(9.86)	45.79(8.5)	46.34(7.55)	48.19(8.01)	49.37(7.81)	49.32(9.19)	50.56(10.63)	49.33(8.36)
	rcp45					44.79(9.92)	46.36(7.46)	50.13(6.96)	50.11(6.94)	52.89(7.18)	54.8(6.5)	55.73(6.63)	56.69(7.05)
	rcp85					43.8(9.22)	48.25(7.56)	50.75(6.85)	53.06(6.44)	58.51(6.04)	61.01(5.94)	65.03(5.28)	66.52(4.92)
**Population**	rcp26					2.5	2.58	2.68	2.77	2.87	2.96	3.05	3.14
**Mean temperature**	rcp26	Q01	-1.17(2.3)	-0.56(2.56)	-0.19(2.04)	-0.18(2.29)	0.32(2.14)	1.31(1.85)	0.42(1.77)	0.56(2.32)	0.91(2.11)	0.92(2.1)	0.57(1.9)
	rcp26	Q02	9.53(1.04)	10.21(1.02)	10.41(0.94)	10.57(1.2)	10.65(1.17)	10.86(1.01)	11.03(1.12)	10.88(1.12)	10.75(1.05)	11.08(1.03)	10.75(0.99)
	rcp26	Q03	14.25(1.04)	15.01(1.11)	15.29(0.94)	15.54(1.08)	15.73(1.1)	15.88(1.02)	16.02(1.11)	16.07(1.19)	16.13(1.33)	16.13(1.33)	15.83(1.05)
	rcp26	Q04	3.03(1.25)	3.76(1.2)	4.1(1.01)	4.21(1.21)	4.51(1.11)	4.69(1.09)	4.66(1.12)	4.54(1.35)	4.73(1.25)	4.62(1.09)	4.23(1.27)
	rcp45	Q01	-1.17(2.3)	-0.43(2.53)	-0.07(2.07)	0.2(2.56)	1.12(1.87)	1.08(2.3)	1.64(1.75)	1.99(2.01)	1.71(1.93)	1.97(2.07)	2.29(1.99)
	rcp45	Q02	9.53(1.04)	10.13(0.95)	10.41(1.09)	10.66(1.09)	11.03(1.15)	11.26(1.14)	11.32(1.09)	11.72(1.31)	11.61(1.11)	11.96(1.17)	11.88(1.05)
	rcp45	Q03	14.25(1.04)	15.05(1.07)	15.38(0.94)	15.7(1.26)	15.83(0.99)	16.49(1.38)	16.17(1.05)	16.87(1.28)	16.92(1.19)	17.03(1.28)	17.16(1.26)
	rcp45	Q04	3.03(1.25)	3.87(1.17)	3.49(1.45)	4.32(0.99)	4.77(1.15)	4.74(1.31)	5.03(1.24)	5.4(1.11)	5.43(1.51)	5.49(1.29)	5.74(1.19)
	rcp85	Q01	-1.17(2.3)	-0.23(2.54)	0.19(2.34)	0.36(2.29)	0.93(2.04)	1.38(2)	2.38(1.83)	2.51(1.71)	3.31(1.68)	4.12(1.59)	4.52(1.64)
	rcp85	Q02	9.53(1.04)	10.2(0.97)	10.42(0.95)	10.74(1.01)	11.19(0.95)	11.41(1.17)	11.84(1.19)	12.35(1.17)	12.49(1.13)	13.14(1.32)	13.71(1.32)
	rcp85	Q03	14.25(1.04)	15.02(1.14)	15.38(0.94)	15.63(1.16)	16.27(1.24)	16.53(1.14)	16.92(1.2)	17.92(1.37)	18.36(1.4)	19.4(1.55)	19.58(1.44)
	rcp85	Q04	3.03(1.25)	3.8(1.21)	4.35(1.28)	4.43(1.13)	4.71(1.14)	4.96(0.98)	5.98(1.22)	6.29(1.4)	6.67(1.36)	7.49(1.24)	7.74(1.34)
**Minimum temperature**	rcp26	Q01	-4.01(2.69)	-3.3(2.92)	-2.83(2.31)	-2.89(2.58)	-2.31(2.36)	-1.23(2.03)	-2.19(1.94)	-1.99(2.53)	-1.7(2.36)	-1.63(2.25)	-1.97(2.12)
	rcp26	Q02	4.68(0.85)	5.34(0.82)	5.57(0.77)	5.7(1.03)	5.84(0.96)	6.03(0.84)	6.15(0.93)	6.04(0.91)	5.94(0.88)	6.18(0.88)	5.93(0.8)
	rcp26	Q03	9.81(0.76)	10.57(0.83)	10.73(0.75)	10.94(0.79)	11.17(0.8)	11.29(0.74)	11.39(0.79)	11.42(0.89)	11.39(0.96)	11.49(1.04)	11.2(0.85)
	rcp26	Q04	0.42(1.41)	1.18(1.36)	1.58(1.16)	1.7(1.36)	2.07(1.22)	2.18(1.21)	2.2(1.21)	2.06(1.45)	2.24(1.36)	2.14(1.19)	1.7(1.42)
	rcp45	Q01	-4.01(2.69)	-3.11(2.87)	-2.72(2.38)	-2.45(2.93)	-1.41(2.05)	-1.44(2.49)	-0.84(1.86)	-0.52(2.11)	-0.77(2.06)	-0.52(2.18)	-0.11(2.17)
	rcp45	Q02	4.68(0.85)	5.29(0.77)	5.58(0.9)	5.83(0.91)	6.21(0.99)	6.41(0.97)	6.43(0.86)	6.8(1.03)	6.8(0.97)	7.1(0.96)	6.99(0.87)
	rcp45	Q03	9.81(0.76)	10.58(0.78)	10.81(0.66)	11.08(0.88)	11.28(0.77)	11.82(1.05)	11.65(0.82)	12.2(1)	12.32(1.01)	12.32(0.99)	12.51(1.02)
	rcp45	Q04	0.42(1.41)	1.29(1.36)	0.91(1.65)	1.83(1.04)	2.37(1.27)	2.25(1.4)	2.59(1.29)	2.99(1.21)	2.98(1.58)	3.05(1.36)	3.36(1.27)
	rcp85	Q01	-4.01(2.69)	-2.91(2.89)	-2.45(2.63)	-2.26(2.5)	-1.59(2.22)	-1.15(2.13)	-0.1(1.93)	0.09(1.81)	0.91(1.77)	1.72(1.6)	2.08(1.69)
	rcp85	Q02	4.68(0.85)	5.37(0.81)	5.59(0.78)	5.91(0.81)	6.3(0.79)	6.56(0.99)	6.97(0.98)	7.5(0.99)	7.72(0.97)	8.33(1.08)	8.84(1.02)
	rcp85	Q03	9.81(0.76)	10.56(0.87)	10.8(0.7)	11.15(0.85)	11.61(0.98)	11.96(0.86)	12.35(0.87)	13.25(1.05)	13.73(1.08)	14.61(1.22)	14.87(1.2)
	rcp85	Q04	0.42(1.41)	1.22(1.38)	1.86(1.4)	1.94(1.24)	2.25(1.24)	2.55(1.02)	3.58(1.27)	3.9(1.45)	4.34(1.37)	5.11(1.22)	5.4(1.34)
**Maximun temperature**	rcp26	Q01	1.75(2.06)	2.31(2.4)	2.63(1.87)	2.7(2.13)	3.17(2.05)	4.07(1.76)	3.24(1.69)	3.31(2.17)	3.73(1.98)	3.7(2.04)	3.34(1.81)
	rcp26	Q02	14.68(1.29)	15.38(1.33)	15.54(1.19)	15.74(1.49)	15.77(1.46)	15.98(1.28)	16.23(1.43)	16.01(1.42)	15.86(1.3)	16.28(1.29)	15.84(1.26)
	rcp26	Q03	18.99(1.34)	19.69(1.43)	20.02(1.19)	20.31(1.45)	20.49(1.47)	20.62(1.42)	20.81(1.46)	20.86(1.55)	21.01(1.78)	20.91(1.68)	20.6(1.35)
	rcp26	Q04	5.63(1.18)	6.38(1.12)	6.67(0.92)	6.78(1.13)	7.03(1.09)	7.28(1.03)	7.2(1.07)	7.1(1.3)	7.31(1.2)	7.19(1.05)	6.82(1.19)
	rcp45	Q01	1.75(2.06)	2.41(2.35)	2.72(1.92)	3.01(2.32)	3.84(1.78)	3.8(2.17)	4.34(1.75)	4.79(2)	4.42(1.88)	4.7(2.01)	4.97(1.9)
	rcp45	Q02	14.68(1.29)	15.27(1.22)	15.55(1.38)	15.82(1.35)	16.15(1.39)	16.43(1.4)	16.52(1.41)	16.95(1.7)	16.73(1.35)	17.14(1.48)	17.08(1.35)
	rcp45	Q03	18.99(1.34)	19.76(1.39)	20.17(1.32)	20.49(1.65)	20.52(1.26)	21.27(1.73)	20.85(1.39)	21.65(1.63)	21.64(1.45)	21.82(1.67)	21.92(1.58)
	rcp45	Q04	5.63(1.18)	6.49(1.07)	6.12(1.34)	6.89(0.98)	7.25(1.09)	7.31(1.27)	7.56(1.22)	7.9(1.07)	7.98(1.49)	8.01(1.25)	8.21(1.15)
	rcp85	Q01	1.75(2.06)	2.6(2.38)	2.96(2.2)	3.16(2.21)	3.68(1.96)	4.1(1.96)	5.1(1.85)	5.23(1.71)	6(1.68)	6.82(1.68)	7.23(1.68)
	rcp85	Q02	14.68(1.29)	15.34(1.24)	15.58(1.21)	15.87(1.29)	16.37(1.22)	16.58(1.45)	17.03(1.52)	17.53(1.47)	17.61(1.4)	18.28(1.66)	18.92(1.7)
	rcp85	Q03	18.99(1.34)	19.72(1.45)	20.14(1.28)	20.29(1.51)	21.05(1.59)	21.23(1.45)	21.6(1.61)	22.68(1.76)	23.07(1.8)	24.19(1.92)	24.25(1.79)
	rcp85	Q04	5.63(1.18)	6.42(1.11)	6.92(1.23)	7(1.08)	7.26(1.09)	7.49(1.02)	8.49(1.19)	8.8(1.37)	9.13(1.35)	9.96(1.25)	10.17(1.34)

Table S15: Ensemble Mean of TBE cases,Tbe incidence rates, Population and climatic indicators by quarter, RCP and period in Middle and Upper North in Sweden 2000-2090

				Decade									
	rcp	Quarter	1970-1999	2000	2010	2020	2030	2040	2050	2060	2070	2080	2090
**TBE incidence rate**	rcp26					0.32(0.18)	0.38(0.22)	0.38(0.22)	0.42(0.23)	0.43(0.25)	0.43(0.26)	0.44(0.27)	0.4(0.25)
	rcp45					0.39(0.22)	0.4(0.22)	0.49(0.29)	0.49(0.23)	0.54(0.28)	0.64(0.32)	0.62(0.29)	0.61(0.3)
	rcp85					0.35(0.21)	0.46(0.26)	0.55(0.26)	0.62(0.25)	0.85(0.31)	0.92(0.36)	1.08(0.37)	1.12(0.33)
**Predicted tbe cases**	rcp26					2.83(1.6)	3.38(1.97)	3.36(1.96)	3.78(2.08)	3.95(2.28)	4.08(2.44)	4.32(2.6)	4.06(2.53)
	rcp45					3.51(1.96)	3.57(1.94)	4.38(2.53)	4.38(2.1)	5.02(2.55)	6.1(3.06)	6.11(2.89)	6.12(2.99)
	rcp85					3.17(1.88)	4.05(2.28)	4.91(2.32)	5.56(2.21)	7.8(2.89)	8.79(3.43)	10.53(3.65)	11.28(3.31)
**Population**	rcp26					0.89	0.89	0.89	0.9	0.92	0.95	0.98	1.01
**Mean temperature**	rcp26	Q01	-9.31(2.4)	-8.43(2.87)	-7.57(2.37)	-7.54(2.41)	-7.19(2.13)	-6.23(1.86)	-7.01(2.02)	-6.86(2.28)	-6.76(2.44)	-6.62(2.16)	-6.97(2.3)
	rcp26	Q02	4.29(1.08)	5.43(1.19)	5.45(0.95)	5.82(1.24)	5.85(1.24)	6.05(1.02)	6.18(1.28)	6.02(1.33)	6.05(1.19)	6.18(1.14)	5.98(1.34)
	rcp26	Q03	9.64(0.96)	10.36(0.99)	10.71(1.05)	10.89(0.87)	11(1.09)	11.19(1.02)	11.4(1.06)	11.34(1.09)	11.43(1.22)	11.37(1.29)	11.32(1.27)
	rcp26	Q04	-5.02(1.61)	-3.97(1.59)	-3.16(1.49)	-3.12(1.59)	-2.88(1.57)	-2.5(1.35)	-2.61(1.45)	-2.7(1.52)	-2.42(1.45)	-2.45(1.5)	-2.91(1.54)
	rcp45	Q01	-9.31(2.4)	-8.25(2.69)	-7.81(2.24)	-7.17(2.27)	-6.79(1.99)	-6.38(2.43)	-6.04(1.91)	-5.35(2.18)	-5.33(2.03)	-5.32(2.15)	-4.77(2.24)
	rcp45	Q02	4.29(1.08)	5.15(1.12)	5.47(1.15)	5.8(1.22)	6.19(1.42)	6.58(1.09)	6.72(1.26)	7.03(1.42)	7.15(1.27)	7.41(1.36)	7.35(1.27)
	rcp45	Q03	9.64(0.96)	10.34(0.98)	10.7(0.73)	11.12(1.05)	11.27(0.91)	11.67(1.28)	11.56(1.01)	12(1.19)	12.39(1.37)	12.31(1.3)	12.42(1.28)
	rcp45	Q04	-5.02(1.61)	-3.82(1.51)	-4.01(1.54)	-3.19(1.4)	-2.65(1.54)	-2.48(1.55)	-2.09(1.51)	-1.75(1.43)	-1.34(1.67)	-1.54(1.58)	-1.12(1.37)
	rcp85	Q01	-9.31(2.4)	-8.1(2.79)	-7.64(2.86)	-7.22(2.14)	-6.46(2.31)	-5.8(2.07)	-5.14(2.03)	-4.52(1.97)	-3.63(1.89)	-2.98(1.82)	-2.25(1.83)
	rcp85	Q02	4.29(1.08)	5.24(1.1)	5.54(1.1)	6(1.19)	6.41(1.12)	6.81(1.29)	7.34(1.41)	7.85(1.36)	8.42(1.39)	8.97(1.56)	9.78(1.58)
	rcp85	Q03	9.64(0.96)	10.31(1.03)	10.7(0.87)	11.07(1.03)	11.49(1.13)	11.92(1.02)	12.28(1.06)	13.21(1.3)	13.69(1.56)	14.45(1.6)	14.72(1.61)
	rcp85	Q04	-5.02(1.61)	-3.92(1.61)	-2.93(1.55)	-2.73(1.4)	-2.33(1.34)	-2.07(1.31)	-0.81(1.49)	-0.59(1.75)	0.1(1.57)	1.09(1.56)	1.41(1.7)
**Minimum temperature**	rcp26	Q01	-13.55(2.57)	-12.48(3.09)	-11.5(2.57)	-11.44(2.67)	-11.05(2.34)	-10.02(2.12)	-10.78(2.23)	-10.61(2.51)	-10.53(2.62)	-10.36(2.42)	-10.74(2.54)
	rcp26	Q02	-0.19(1.04)	0.91(1.04)	1.01(0.91)	1.29(1.16)	1.41(1.24)	1.64(0.99)	1.74(1.2)	1.62(1.26)	1.6(1.12)	1.75(1.08)	1.55(1.3)
	rcp26	Q03	5.75(0.85)	6.52(0.88)	6.84(0.95)	7.03(0.81)	7.16(0.97)	7.33(0.94)	7.5(0.95)	7.41(0.95)	7.49(1.01)	7.46(1.11)	7.38(1.21)
	rcp26	Q04	-8.29(1.79)	-7.15(1.75)	-6.23(1.66)	-6.15(1.78)	-5.84(1.76)	-5.49(1.51)	-5.56(1.61)	-5.64(1.67)	-5.39(1.58)	-5.37(1.67)	-5.9(1.67)
	rcp45	Q01	-13.55(2.57)	-12.22(2.89)	-11.76(2.45)	-10.99(2.54)	-10.58(2.21)	-10.13(2.71)	-9.69(2.16)	-8.92(2.39)	-8.83(2.29)	-8.86(2.39)	-8.21(2.51)
	rcp45	Q02	-0.19(1.04)	0.64(0.99)	1(1.1)	1.36(1.14)	1.79(1.28)	2.15(1.06)	2.31(1.2)	2.62(1.29)	2.8(1.22)	3.04(1.3)	3.01(1.17)
	rcp45	Q03	5.75(0.85)	6.51(0.87)	6.83(0.73)	7.23(0.92)	7.43(0.82)	7.79(1.01)	7.79(0.94)	8.2(1.06)	8.55(1.16)	8.44(1.14)	8.58(1.11)
	rcp45	Q04	-8.29(1.79)	-7.02(1.64)	-7.1(1.64)	-6.25(1.56)	-5.57(1.68)	-5.42(1.71)	-4.94(1.67)	-4.61(1.63)	-4.12(1.8)	-4.35(1.74)	-3.89(1.5)
	rcp85	Q01	-13.55(2.57)	-12.06(2.97)	-11.56(3.1)	-11.01(2.35)	-10.19(2.6)	-9.38(2.28)	-8.7(2.21)	-7.86(2.22)	-6.85(2.15)	-6.03(2.06)	-5.16(2.03)
	rcp85	Q02	-0.19(1.04)	0.75(0.95)	1.04(0.97)	1.56(1.12)	1.95(1.01)	2.44(1.23)	2.98(1.27)	3.54(1.29)	4.11(1.28)	4.73(1.46)	5.48(1.49)
	rcp85	Q03	5.75(0.85)	6.5(0.91)	6.82(0.75)	7.25(0.93)	7.64(1.02)	8.12(0.88)	8.53(0.94)	9.36(1.09)	9.83(1.24)	10.55(1.29)	10.89(1.31)
	rcp85	Q04	-8.29(1.79)	-7.1(1.79)	-5.97(1.71)	-5.71(1.55)	-5.24(1.52)	-4.94(1.39)	-3.57(1.64)	-3.29(1.9)	-2.5(1.71)	-1.42(1.7)	-1.07(1.83)
**Maximun temperature**	rcp26	Q01	-5.39(2.2)	-4.68(2.64)	-3.95(2.14)	-3.97(2.13)	-3.63(1.93)	-2.77(1.61)	-3.54(1.78)	-3.41(2.03)	-3.28(2.21)	-3.18(1.89)	-3.5(2.06)
	rcp26	Q02	8.72(1.2)	9.89(1.41)	9.85(1.06)	10.31(1.41)	10.27(1.35)	10.47(1.16)	10.61(1.46)	10.42(1.5)	10.48(1.35)	10.61(1.29)	10.41(1.49)
	rcp26	Q03	13.76(1.15)	14.43(1.17)	14.82(1.21)	14.96(0.99)	15.06(1.24)	15.25(1.15)	15.49(1.19)	15.45(1.22)	15.57(1.45)	15.5(1.49)	15.44(1.35)
	rcp26	Q04	-1.98(1.49)	-1(1.49)	-0.27(1.37)	-0.24(1.47)	-0.03(1.45)	0.35(1.27)	0.21(1.37)	0.12(1.44)	0.43(1.38)	0.37(1.4)	-0.06(1.47)
	rcp45	Q01	-5.39(2.2)	-4.55(2.47)	-4.18(2.01)	-3.68(2)	-3.33(1.77)	-2.97(2.13)	-2.72(1.68)	-2.09(1.93)	-2.14(1.78)	-2.11(1.89)	-1.65(2.01)
	rcp45	Q02	8.72(1.2)	9.6(1.33)	9.89(1.29)	10.21(1.39)	10.59(1.62)	11.04(1.24)	11.17(1.44)	11.47(1.63)	11.59(1.42)	11.85(1.54)	11.76(1.43)
	rcp45	Q03	13.76(1.15)	14.41(1.14)	14.81(0.84)	15.23(1.22)	15.3(1.04)	15.74(1.52)	15.57(1.12)	16(1.31)	16.42(1.54)	16.35(1.38)	16.45(1.42)
	rcp45	Q04	-1.98(1.49)	-0.83(1.42)	-1.08(1.47)	-0.3(1.33)	0.14(1.47)	0.34(1.47)	0.64(1.43)	1(1.3)	1.38(1.63)	1.18(1.5)	1.58(1.3)
	rcp85	Q01	-5.39(2.2)	-4.42(2.56)	-4.04(2.54)	-3.76(1.93)	-3.01(2.02)	-2.52(1.84)	-1.9(1.81)	-1.43(1.76)	-0.68(1.69)	-0.14(1.67)	0.47(1.72)
	rcp85	Q02	8.72(1.2)	9.69(1.32)	9.99(1.32)	10.43(1.35)	10.88(1.31)	11.23(1.44)	11.78(1.61)	12.27(1.51)	12.83(1.53)	13.34(1.72)	14.18(1.68)
	rcp85	Q03	13.76(1.15)	14.37(1.2)	14.8(1.04)	15.13(1.16)	15.54(1.24)	15.94(1.15)	16.23(1.19)	17.23(1.43)	17.69(1.74)	18.43(1.77)	18.66(1.78)
	rcp85	Q04	-1.98(1.49)	-0.94(1.5)	-0.05(1.45)	0.09(1.3)	0.44(1.22)	0.69(1.27)	1.88(1.43)	2.08(1.7)	2.68(1.53)	3.63(1.51)	3.95(1.64)

Table S16: Variable selection

Covariate	Beta (CI:low to High)	DIC	logcpo	Rank
**Temperature**				
tmean_Q03_LAG1	0.17566(0.12977 to 0.22189)	10968.78	0.211	1
tmin_Q03_LAG1	0.18437(0.13555 to 0.2334)	10974.95	0.211	2
tmax_Q03_LAG1	0.11748(0.08136 to 0.15388)	10985.09	0.211	3
tmax_Q02_LAG1	0.10973(0.07219 to 0.14774)	10995.80	0.211	4
tmean_Q02_LAG1	0.12433(0.07745 to 0.1717)	11007.05	0.212	5
tmin_Q03	0.14617(0.10202 to 0.1902)	11016.68	0.212	6
tmax_Q02	0.09074(0.06116 to 0.12027)	11019.15	0.212	7
tmin_Q02_LAG1	0.10581(0.05272 to 0.15908)	11023.69	0.212	8
tmean_Q02	0.09133(0.05243 to 0.1302)	11031.65	0.212	9
tmin_Q02	0.0538(0.00699 to 0.10059)	11038.29	0.212	10
tmean_Q03	0.05051(0.00972 to 0.09125)	11039.43	0.212	11
**Precipitation**				
prec_Q03	0.00205(0.00152 to 0.00258)	10980.76	0.211	1
prec_Q02_LAG1	-0.0021(-0.00296 to -0.00124)	11012.25	0.212	2
prec_Q02	-0.00167(-0.00242 to -0.00091)	11024.80	0.212	3
prec_Q03_LAG1	-0.00117(-0.00179 to -0.00055)	11028.36	0.212	4
prec_Q01	0.00084(0.00014 to 0.00153)	11028.63	0.212	5
**Relative humidity**				
RH_Q02	-0.03278(-0.04312 to -0.02243)	11009.48	0.212	1
RH_Q03_LAG1	-0.02981(-0.04303 to -0.01664)	11017.00	0.212	2
RH_Q02_LAG1	-0.02282(-0.03523 to -0.01045)	11026.31	0.212	3
RH_Q03	0.01072(0.00048 to 0.02097)	11031.77	0.212	4
RH_Q04	-0.02878(-0.04779 to -0.00975)	11031.99	0.212	5
**Vegetation**				
fcover_Q03	-1.64996(-2.07806 to -1.2226)	10977.28	0.212	1
fcover_Q01	-4.01774(-4.95802 to -3.07239)	10983.39	0.213	2
lai_Q01	-1.04862(-1.30099 to -0.79518)	10987.51	0.213	3
fcover_Q04_LAG1	-3.74177(-4.60532 to -2.87084)	10987.64	0.212	4
fcover_Q04	-2.84667(-3.63136 to -2.06223)	10991.20	0.212	5
lai_Q04_LAG1	-0.89276(-1.1212 to -0.66272)	10998.84	0.212	6
fcover_Q03_LAG1	-1.30633(-1.78747 to -0.8264)	11000.74	0.212	7
lai_Q04	-0.67838(-0.88421 to -0.47247)	11002.51	0.212	8
lai_Q03	-0.25953(-0.34492 to -0.17422)	11003.80	0.212	9
lai_Q03_LAG1	-0.17466(-0.27083 to -0.07875)	11017.77	0.212	10
fcover_Q02	-0.91723(-1.48197 to -0.3543)	11021.79	0.212	11
fcover_Q02_LAG1	-0.88617(-1.47682 to -0.29764)	11022.49	0.212	12
fcover_Q01_LAG1	-2.08909(-3.04801 to -1.13057)	11024.38	0.213	13
lai_Q01_LAG1	-0.56255(-0.81923 to -0.30591)	11026.17	0.213	14
lai_Q02	-0.10804(-0.21113 to -0.0051)	11031.06	0.212	15
**Normalized Difference Vegetation Index**				
ndvi_Q03	-0.9009(-1.42993 to -0.37452)	11015.39	0.212	1
ndvi_Q01_LAG1	-0.49368(-0.83675 to -0.15224)	11019.45	0.212	2
ndvi_Q04_LAG1	0.54132(0.1947 to 0.88746)	11034.34	0.212	3
ndvi_Q04	0.39317(0.0274 to 0.75879)	11035.26	0.212	4
**Elevation**				
elevation	-0.00874(-0.01105 to -0.00648)	10976.38	0.211	1
**Roe deer density**				
roe	1.2168(0.4591 to 1.97357)	11044.55	0.212	1
roe_LAG1	0.99549(0.26443 to 1.72568)	11044.95	0.212	2

Table S17: Multi collinearity test

Variable	Variance Inflation factor (VIF)
Habitat_richness	1.484422
tmean_Q03_LAG1	1.556405
fcover_Q03	1.447295
prec_Q03	1.059023
roe	1.830069
RH_Q02	1.185780

Dropped variables : ndvi_Q03,elevation

Table S18: Non linear effects final Non-linear model

	β	Credible Interval Lower	Upper
Mean temperature (Q03-LAG1)			
3.33	-1.62992	-5.995	2.5621
5.12	-1.673	-5.0986	1.53137
6.46	-1.7363	-4.47075	0.71142
7.67	-1.79004	-4.06803	0.1357
8.91	-1.76232	-3.86337	-0.0678
10.29	-1.47877	-3.52257	0.18366
11.6	-0.6482	-2.37615	0.97676
12.97	1.30488	-0.08161	3.00521
14.41	2.09446	0.71853	3.78418
15.67	2.38308	1.00563	4.07422
16.77	2.34048	0.95917	4.03433
18.03	2.59693	1.20403	4.30007
Relative Humidity (Q02)			
54.72	0.2098	-0.02149	0.49146
57.44	0.22455	0.04252	0.44403
60.08	0.12167	-0.02786	0.27287
63.15	0.12763	0.01016	0.2614
65.99	0.01417	-0.09529	0.11937
69.1	-0.03058	-0.13019	0.06505
71.92	-0.09999	-0.19954	-0.00925
74.87	-0.153	-0.2728	-0.0441
77.79	-0.09227	-0.21825	0.02744
80.72	-0.08288	-0.2563	0.08659
83.57	-0.11721	-0.38711	0.10955
86.06	-0.12102	-0.46645	0.17166
Habitat richness			
0.15	-0.40557	-1.65788	0.59833
0.23	-0.40907	-1.34822	0.30414
0.29	-0.36419	-1.08906	0.19597
0.33	-0.22853	-0.74192	0.25943
0.38	-0.17896	-0.67294	0.32493
0.45	-0.1305	-0.5093	0.28191
0.48	-0.22103	-0.63537	0.13994
0.53	0.0662	-0.30331	0.48883
0.59	0.21171	-0.16416	0.64136
0.63	0.3919	0.00507	0.84479
0.68	0.58877	0.1297	1.10961
0.72	0.68379	0.11149	1.37912
Forest cover (Q03)			
0	0.72041	0.40228	1.05514
0.1	0.63181	0.40625	0.86768
0.18	0.27107	0.08507	0.45832
0.25	0.00019	-0.20228	0.19154
0.32	-0.03502	-0.18192	0.11029
0.39	-0.18216	-0.31185	-0.05578
0.46	-0.07457	-0.18852	0.03808
0.53	-0.05894	-0.17006	0.05125
0.59	-0.09723	-0.21494	0.02008
0.66	-0.34043	-0.47338	-0.2091
0.72	-0.41689	-0.60122	-0.23601
0.78	-0.41825	-0.82952	-0.02265
Precipitation (Q03)			
95.33	-0.43154	-0.79579	-0.07298
139.22	-0.26624	-0.56259	0.06046
189.74	-0.08115	-0.37268	0.24444
241.86	-0.03744	-0.3269	0.28809
295.09	-0.20393	-0.50658	0.11294
350.77	0.10005	-0.19785	0.438
407.32	0.32243	0.03013	0.68085
463.41	0.1283	-0.24607	0.45902
516.52	0.11145	-0.36494	0.53258
570.69	0.11639	-0.51007	0.68834
631.74	0.12001	-0.67878	0.86652
704.77	0.12215	-0.87972	1.07332
Roe deer density			
0	-1.53982	-1.95819	-1.1255
0.11	-0.37228	-0.64476	-0.09904
0.18	-0.09049	-0.34502	0.16495
0.24	0.07544	-0.16983	0.32179
0.31	0.08871	-0.16204	0.33973
0.38	0.66006	0.41618	0.90623
0.45	0.83247	0.60483	1.0627
0.53	0.39044	0.15373	0.62827
0.6	0.07822	-0.19187	0.34775
0.66	0.09854	-0.26202	0.46025

0.77	-0.09315	-0.60526	0.41622
0.85	-0.12817	-0.95287	0.68681

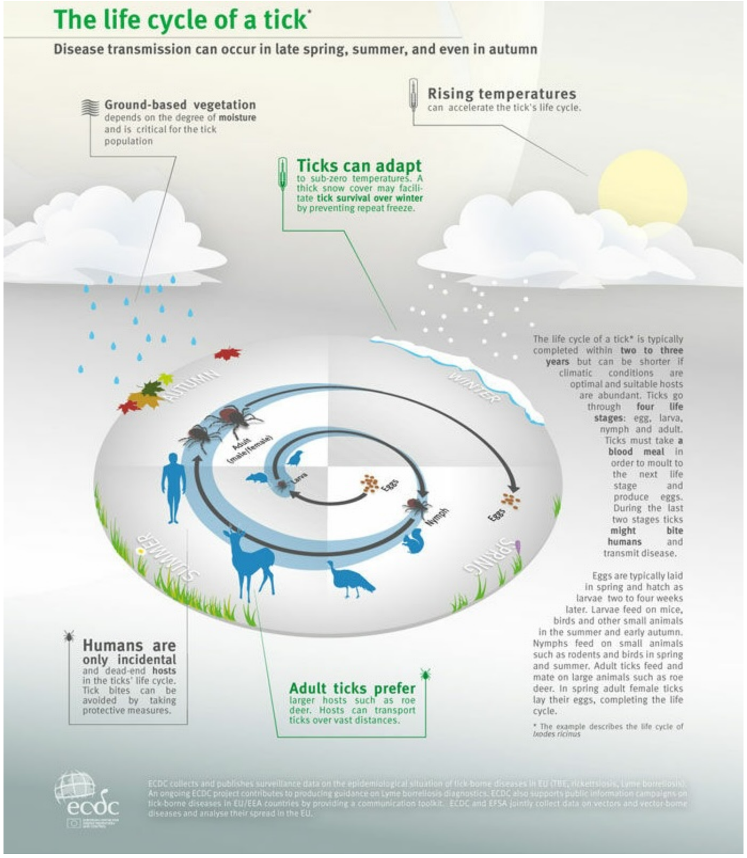

Figure S1: Transmission of tick-borne diseases Infographic. source:https://www.ecdc.europa.eu/en/publications-data/small-bites-big-problems-tick-borne-diseases-europe

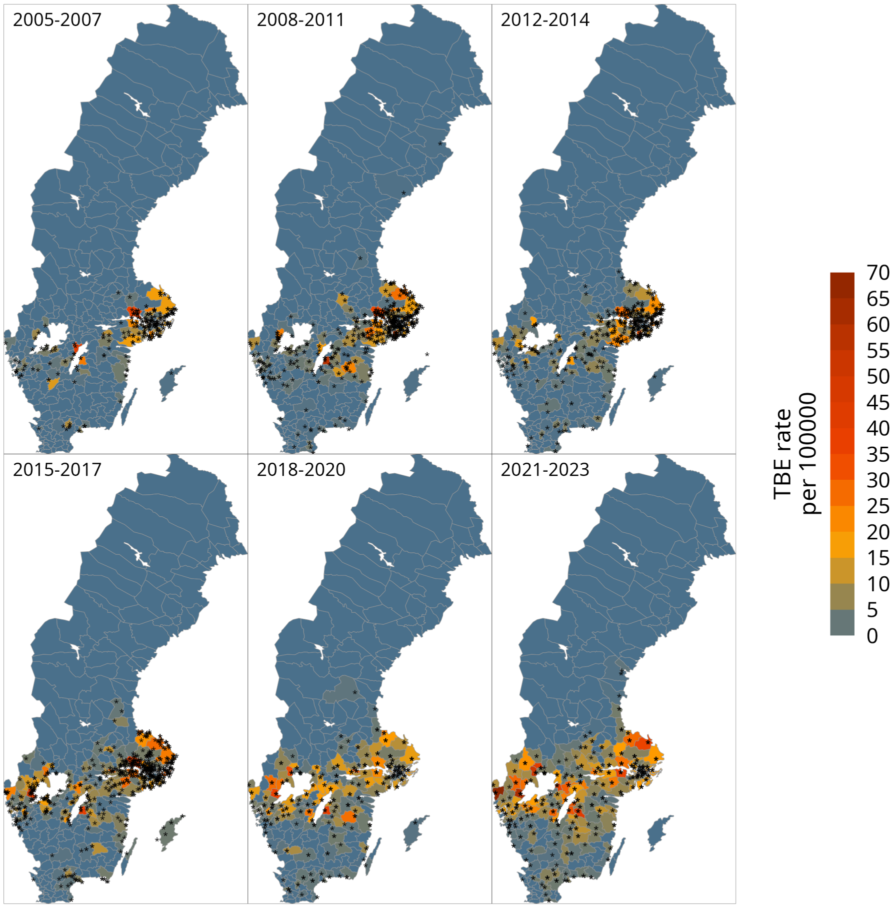

Figure S2: Spatial distribution of Reported TBE cases in Sweden at Kommun (Municipality) by period 2005-2023. The points are the actual geographical points of the reported cases.

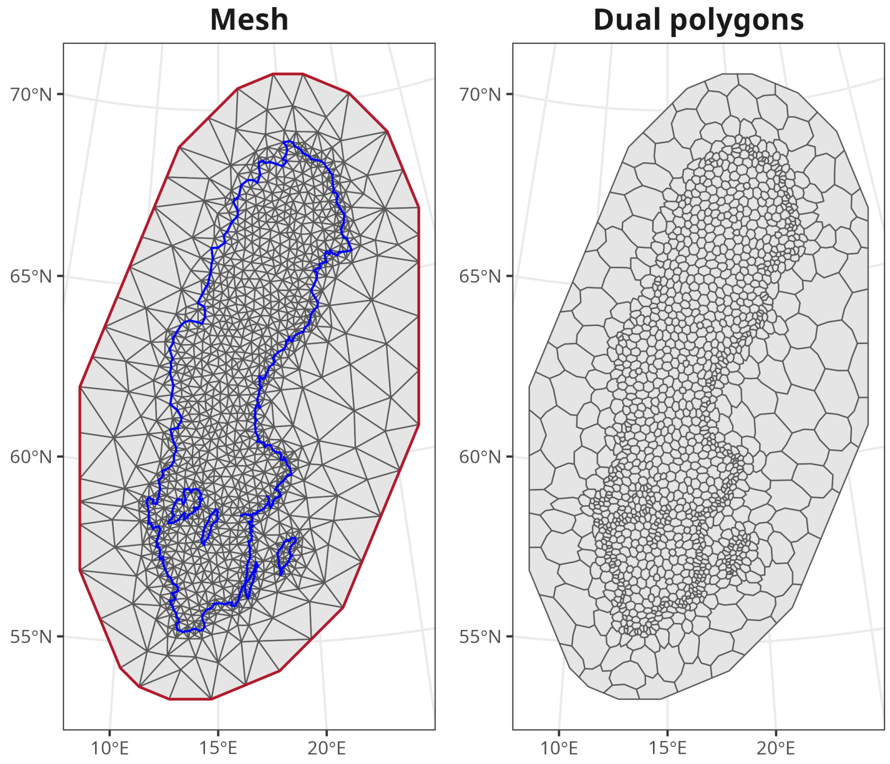

Figure S3: The mesh generated for the whole of Sweden with the corresponding dual polygons used in the log cox modelling of TBE cases in Sweden

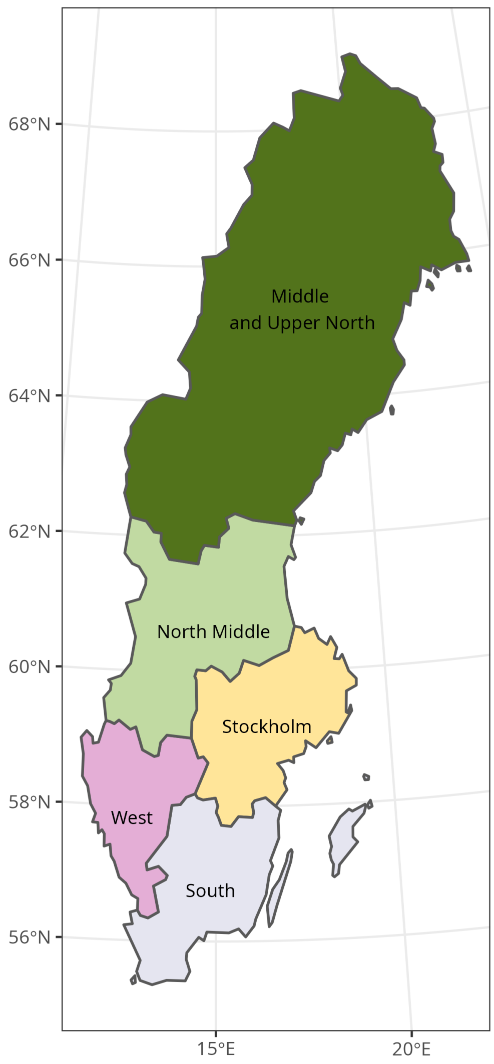

Figure S4: Regions in Sweden

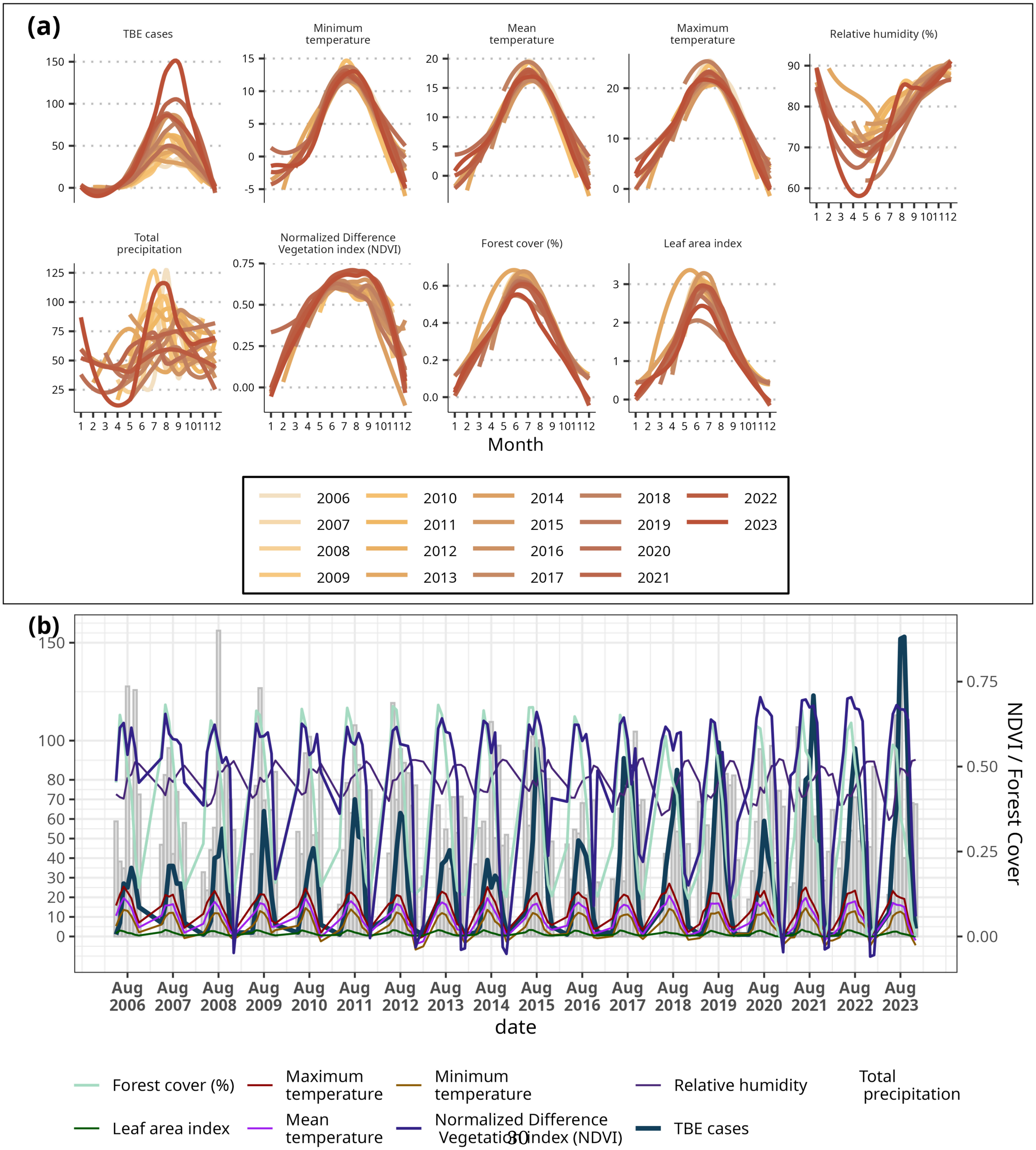

Figure S5: Annual Seasonal pattern of reported TBE cases, minimum temperature, mean temperature, maximum temperature, total precipitation, relative Humidity,normalized difference vegetation index (NDVI), leaf area index and forest cover, in Sweden 2006-2023. (a) is the smooth plot of the seasonal pattern while (b) shows the observed annual seasonal trend 2006-2023.

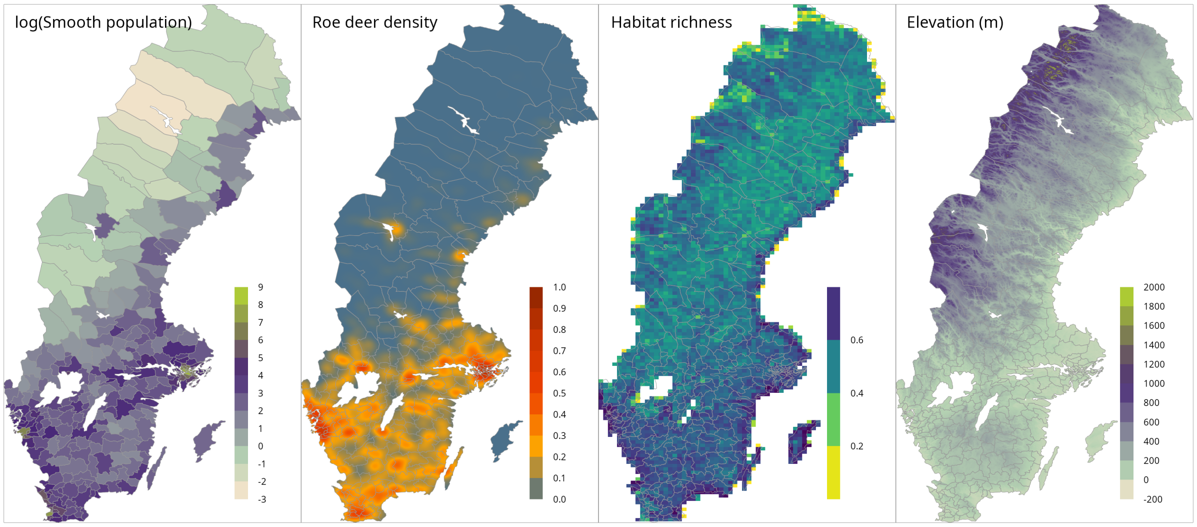

Figure S6: Spatial distribution log population estimates ,roe deer density,Habitat richness and Elevation in Sweden 2023

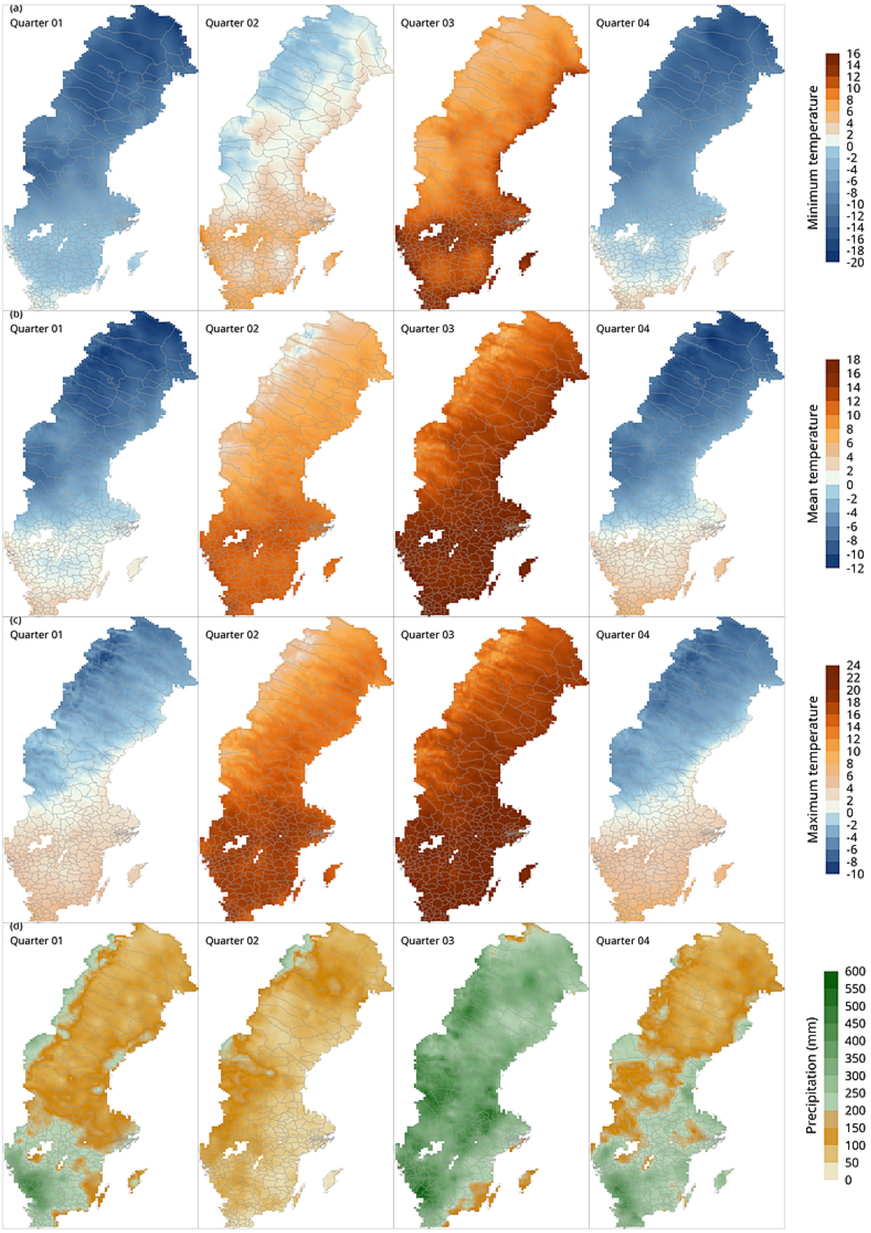

Figure S7: Spatial distribution in 2023 by quarter of (a) minimum temperature, (b) mean temperature, (c) maximum temperature and (d) total precipitation in Sweden

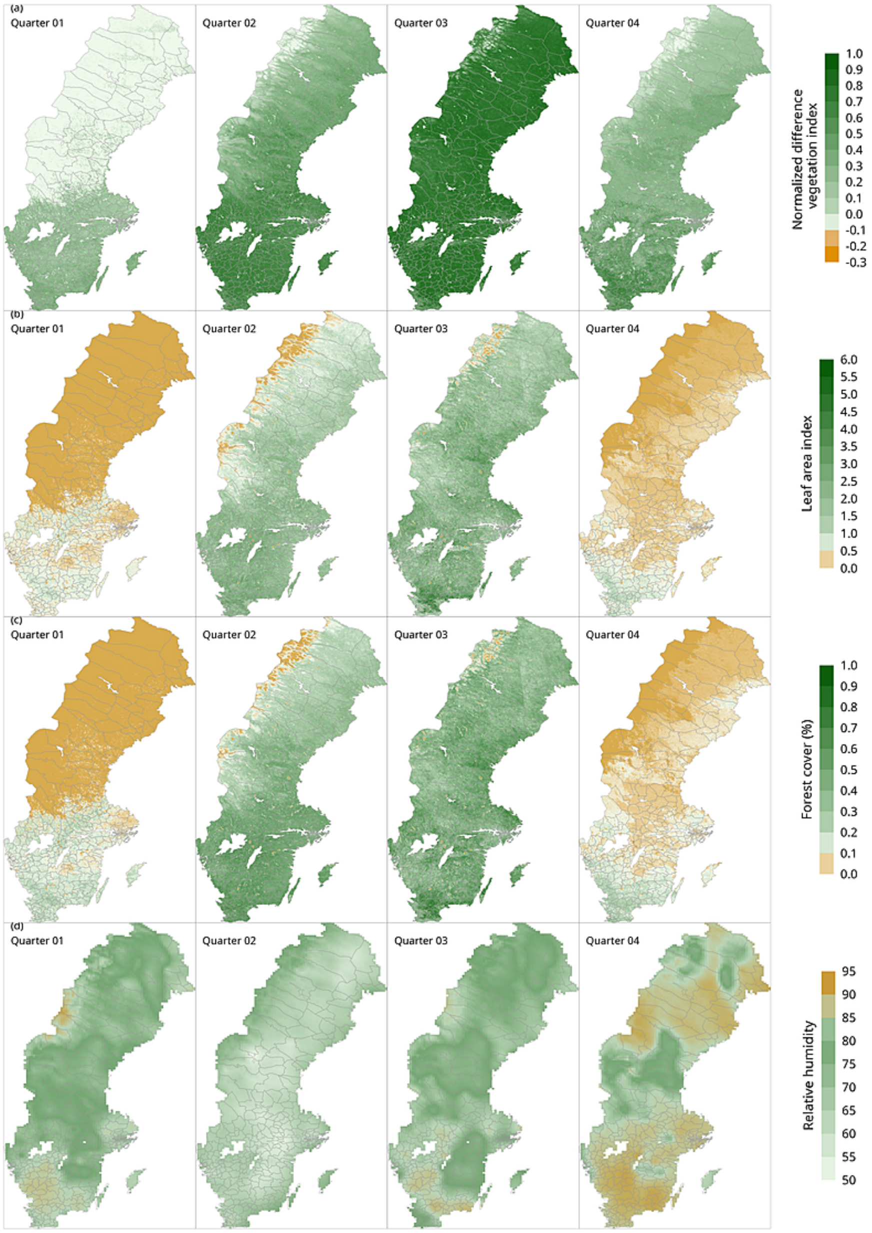

Figure S8: Spatial distribution in 2023 by quarter of (a) Normalized difference vegetation Index (NDVI), (b) Leaf area Index (LAI) , (c) Forest cover and (d) Relative humidity in Sweden

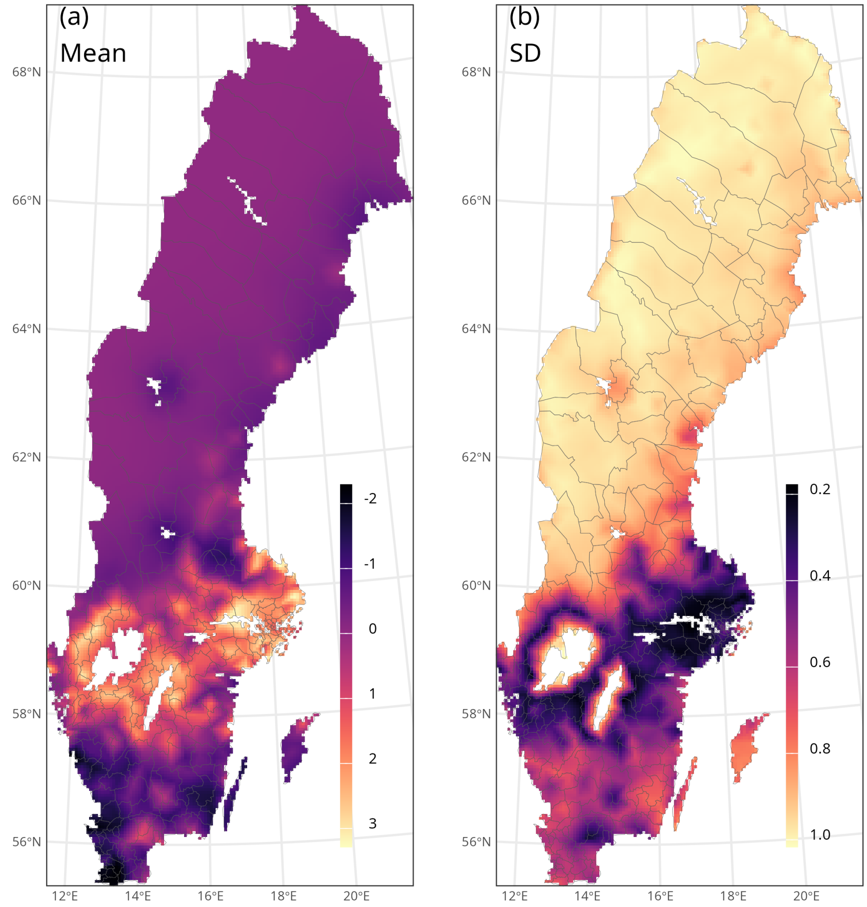

Figure S9: The model estimated mean (a) and standard deviation (b) of the spatial field of the final selected TBE model.

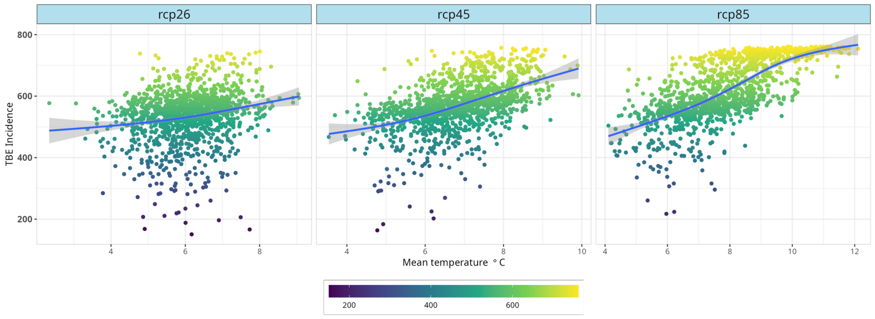

Figure S10: Scatter plot of Projected TBE incidence and temperature by RCP26, RCP45 and RCP85

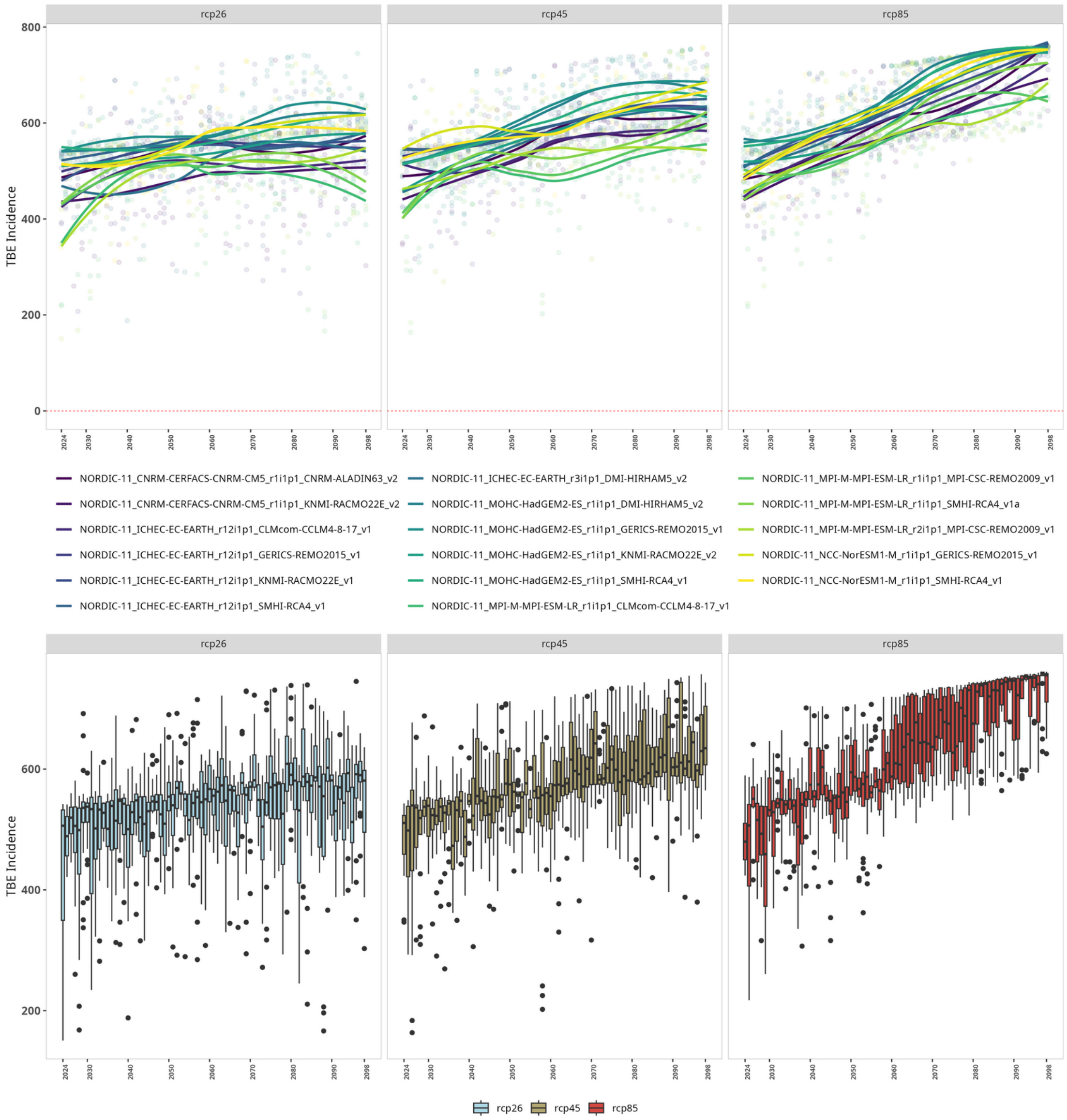

Figure S11: Projected TBE Incidence by RCP26, RCP45 and RCP85. The first panel shows smoothed estimate from each regional climate model. The second panel is the box plot of the projected TBE incidence , showing distribution based on regional climate models while the third panel shows the smoothed ensemble mean projected TBE incidence, the shaded region represents the minimum and maximum projected incidence from the regional climate models

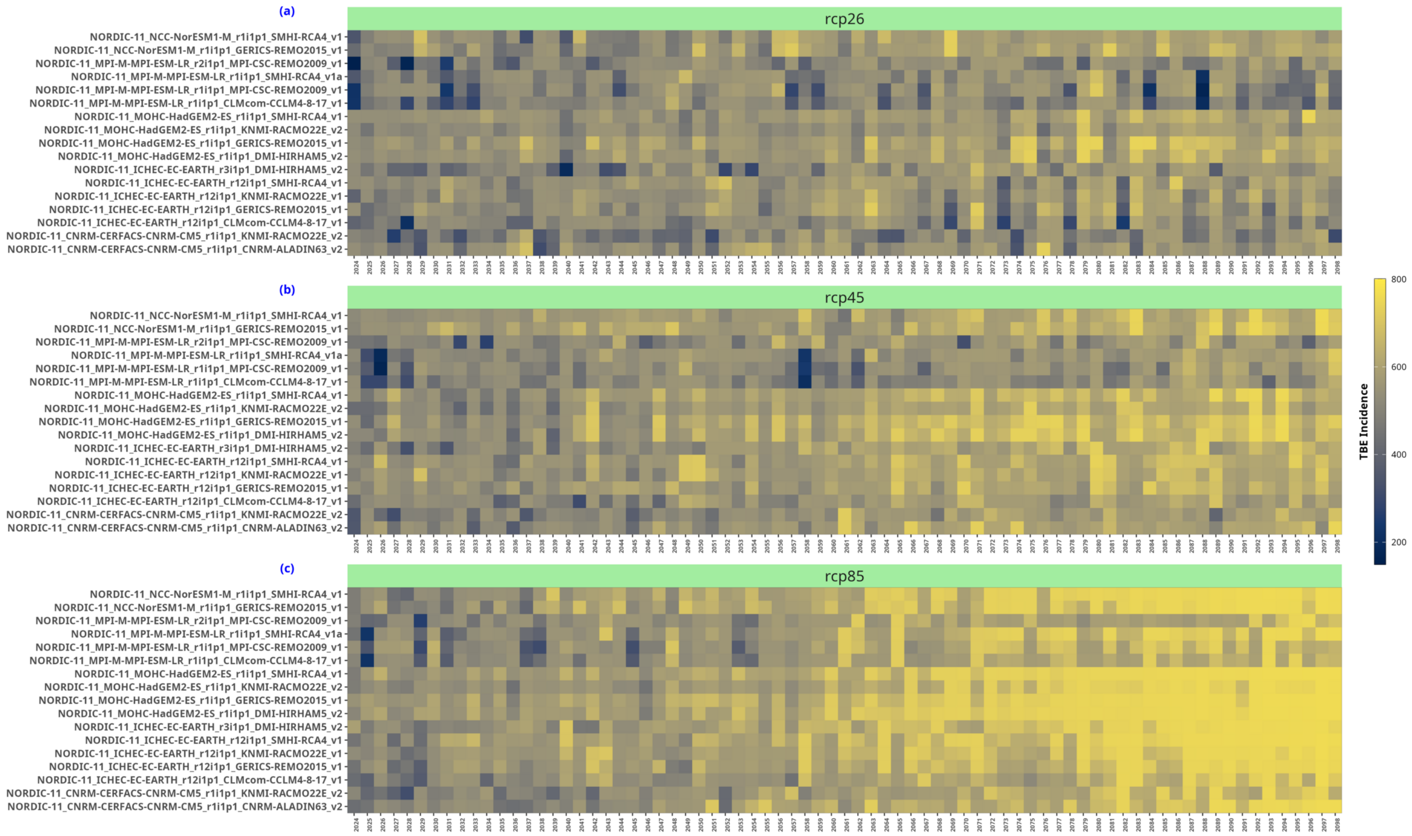

Figure S12: Raster plot of Projected TBE Incidence in Sweden by (a) RCP26, (b) RCP45 and (c) RCP85 for each regional climate model for the years 2024-2098

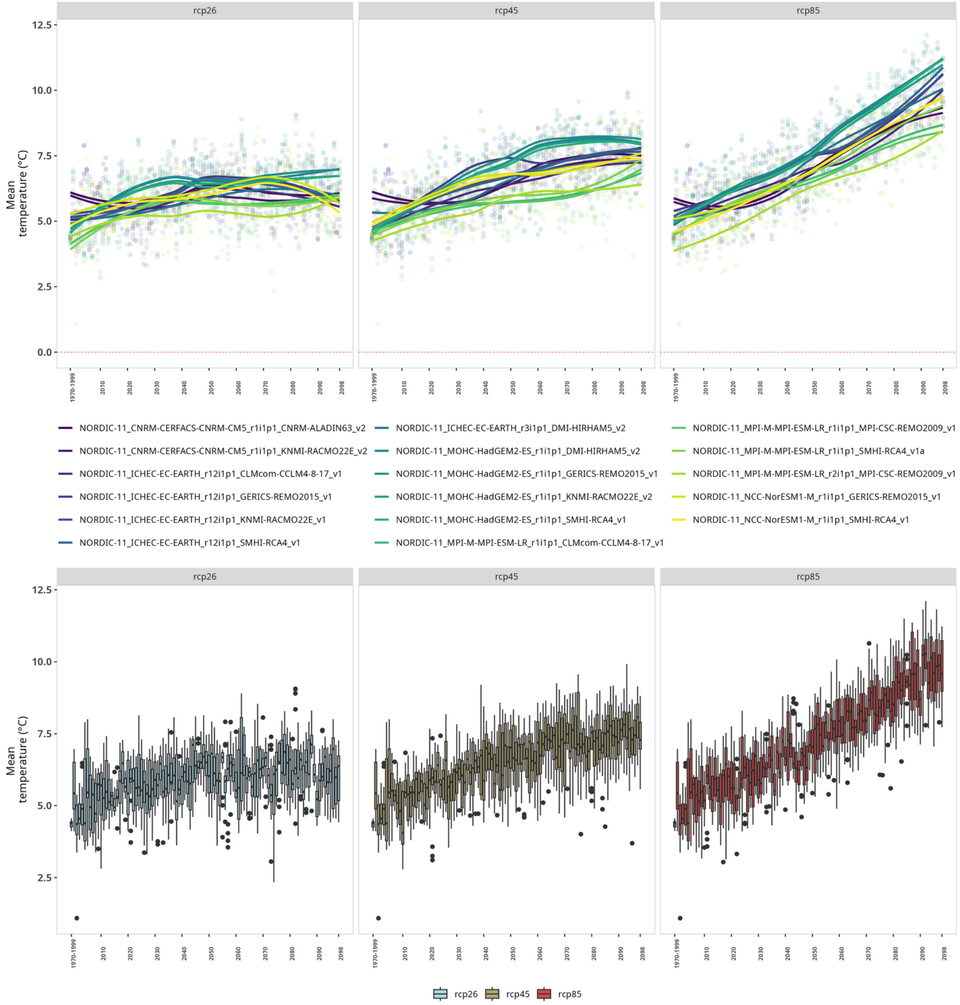

Figure S13: Mean temperature projections by RCP26, RCP45 and RCP85 for each regional climate model for the period 2000-2098 in comparison to the baseline period 1970-1990. The first panel shows smoothed estimate from each regional climate model. The second panel is the box plot of the projected mean temperature, showing distribution based on regional climate models.

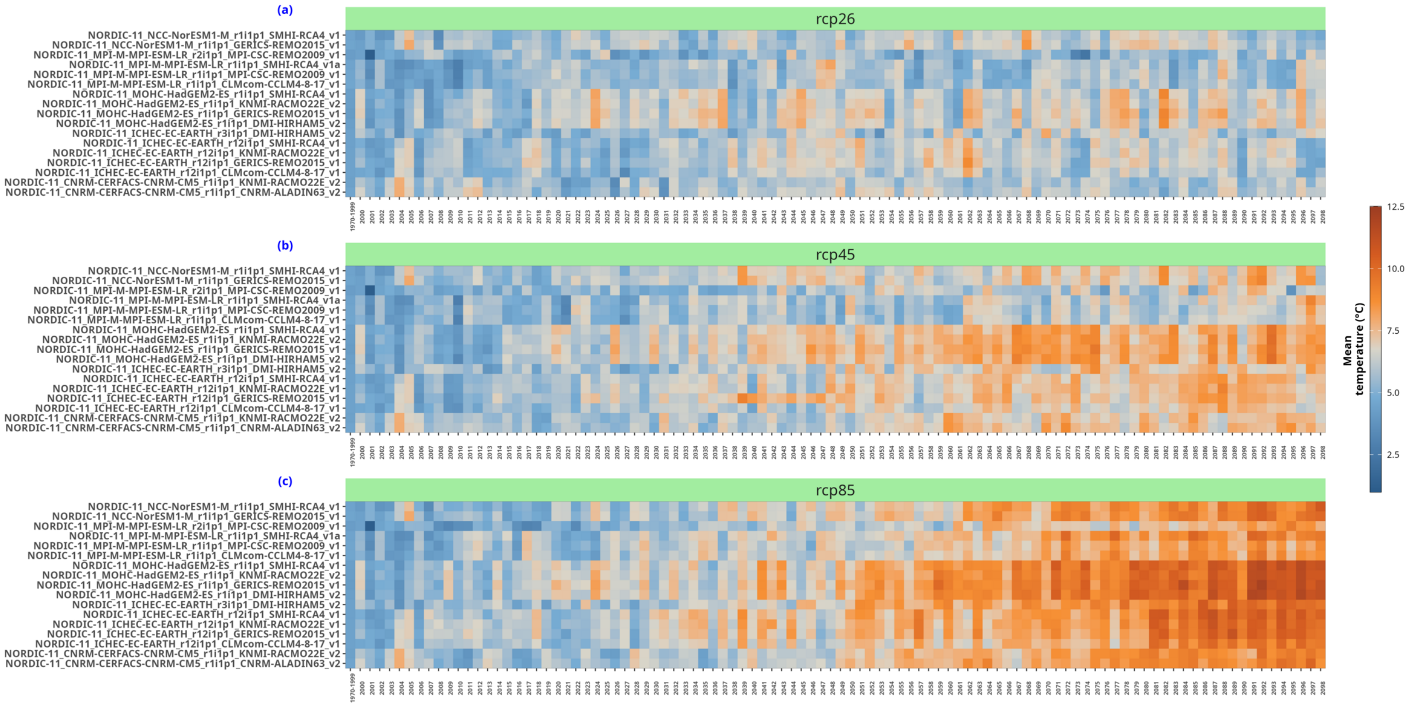

Figure S14: Raster plot of mean temperature projections in Sweden by (a) RCP26, (b) RCP45 and (c) RCP85 for each regional climate model for the years 2000-2098. Also included in the plot is the baseline period 1970-1990

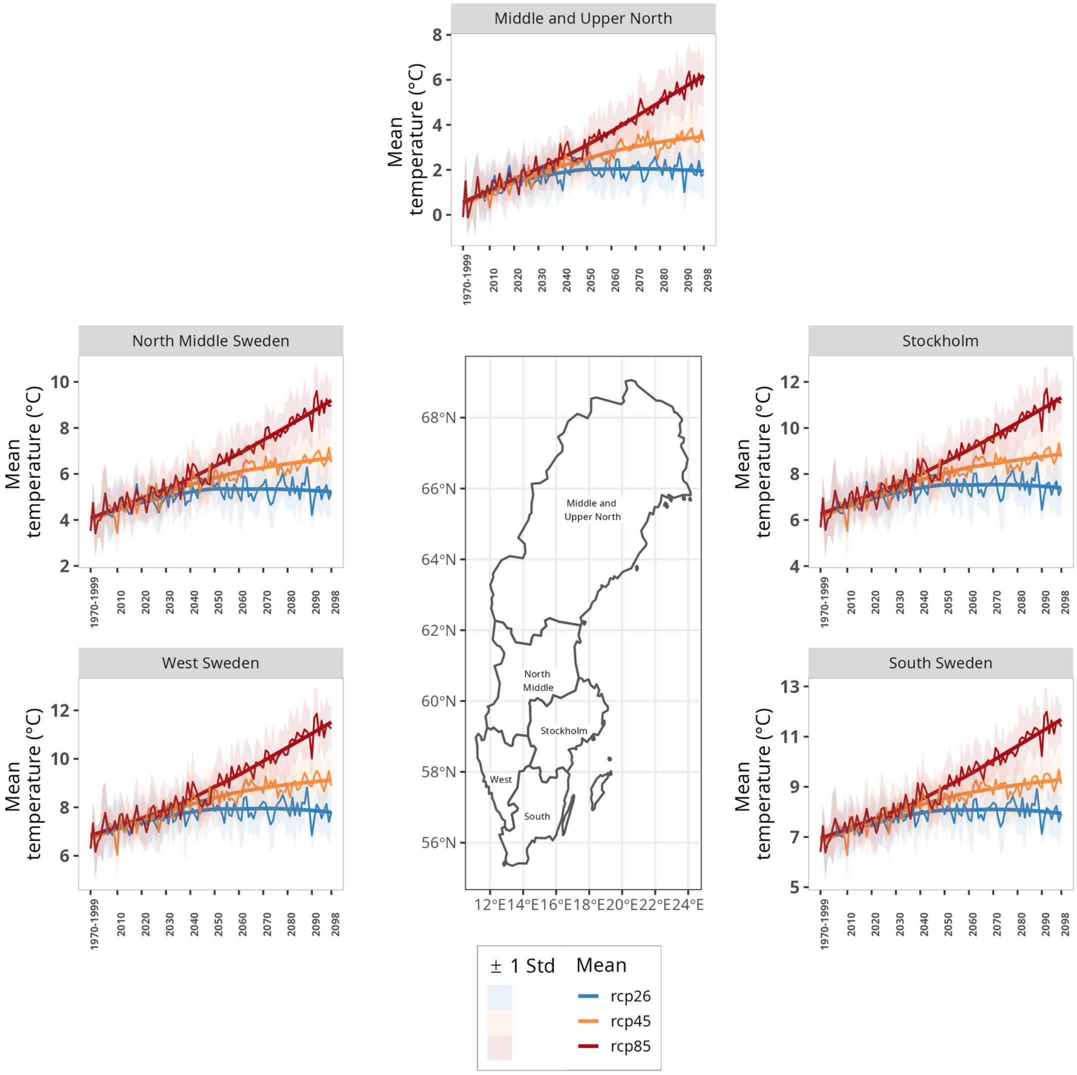

Figure S15: Mean temperature projection trends by RCP and regions in Sweden for the period 2000-2098. The shaded regions represent uncertainty (± 1) standard deviations computed from the contributing regional climate circulation models. The baseline period 1970-1999 is included for comparison.

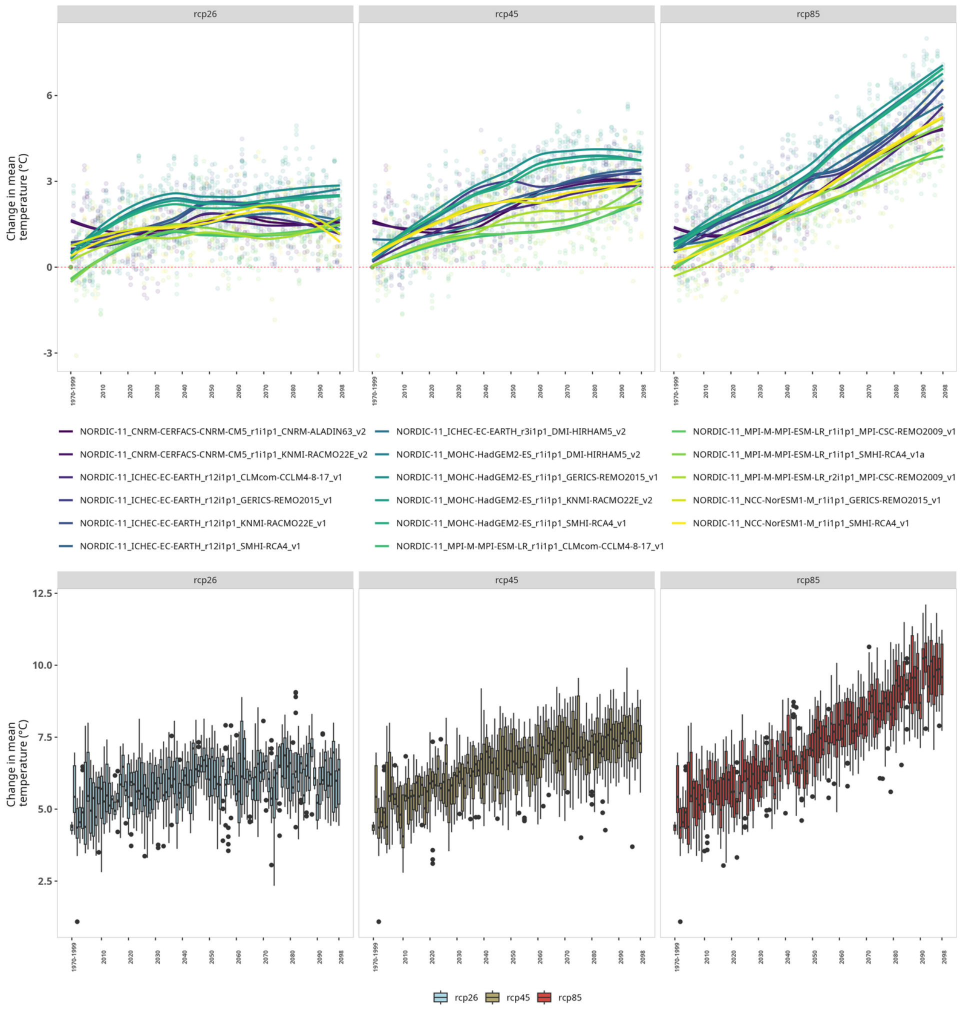

Figure S16: Projections in mean temperature change by RCP26, RCP45 and RCP85 for each regional climate model for the period 2000-2098 in comparison to the baseline period 1970-1990. The first panel shows smoothed estimate from each regional climate model. The second panel is the box plot showing the distribution of the projected change in mean temperature based on the regional climate models.

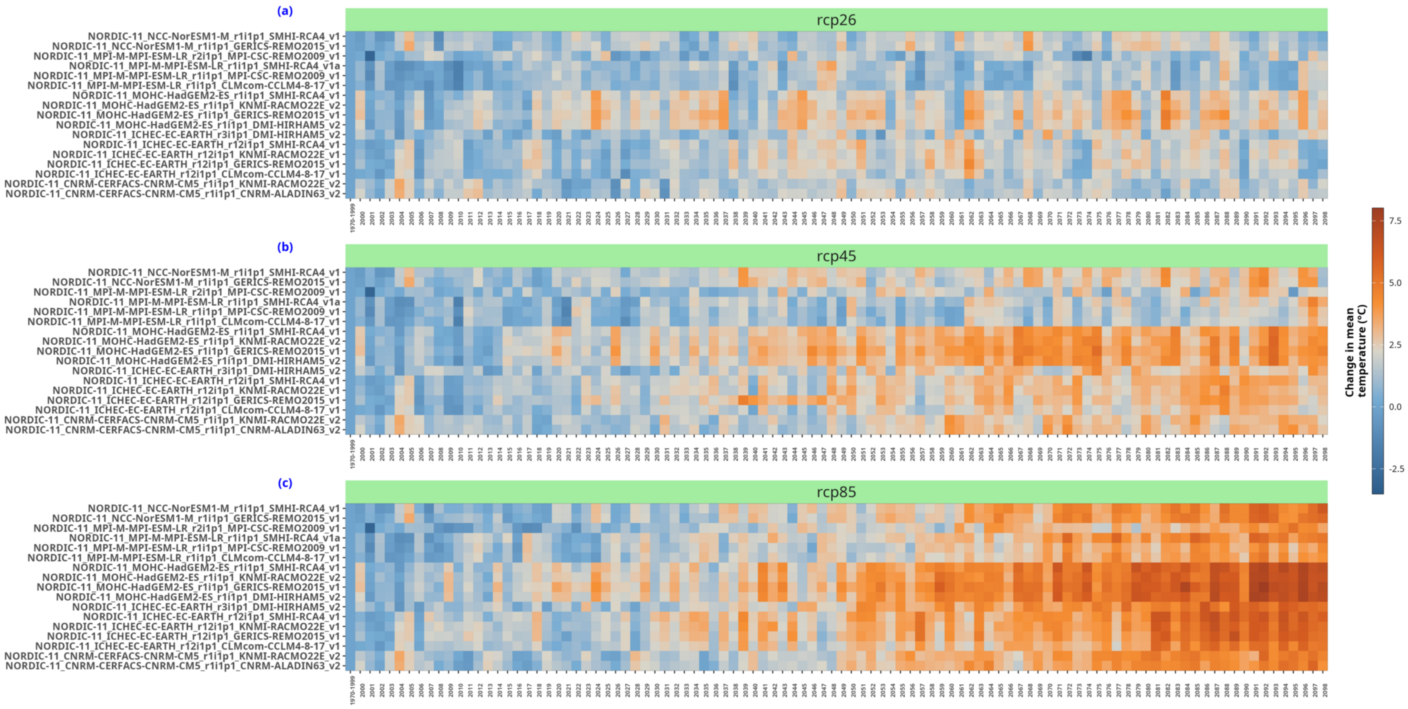

Figure S17: Raster plot of change mean temperature projections in Sweden by (a) RCP26, (b) RCP45 and (c) RCP85 for each regional climate model for the years 2000-2098 in reference to the baseline period 1970-1999.

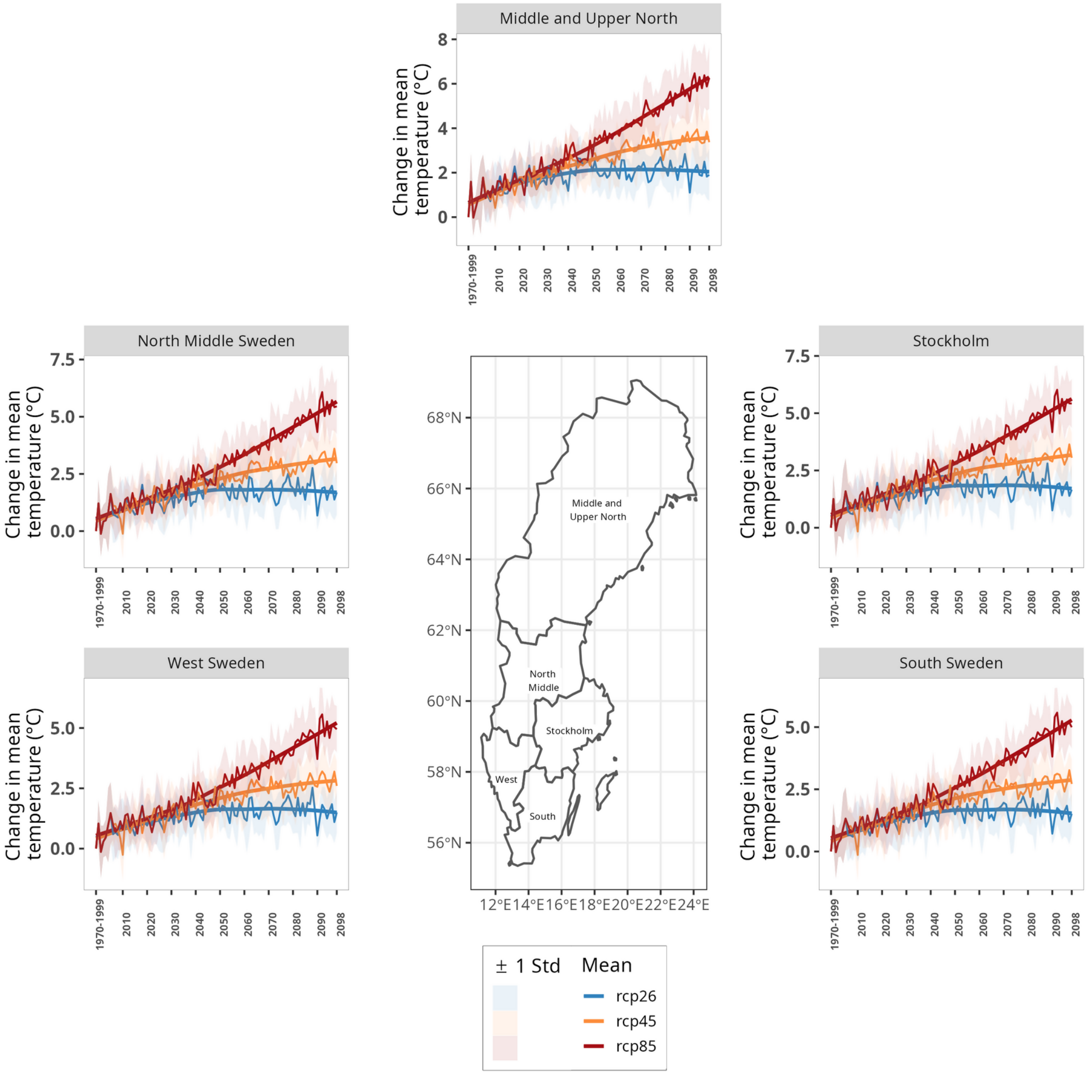

Figure S18: Change in mean temperature projection trends by RCP and regions in Sweden for the period 2000-2098 in reference to the baseline period 1970-1999. The shaded regions represent uncertainty (± 1) standard deviations computed from the contributing regional climate circulation models.

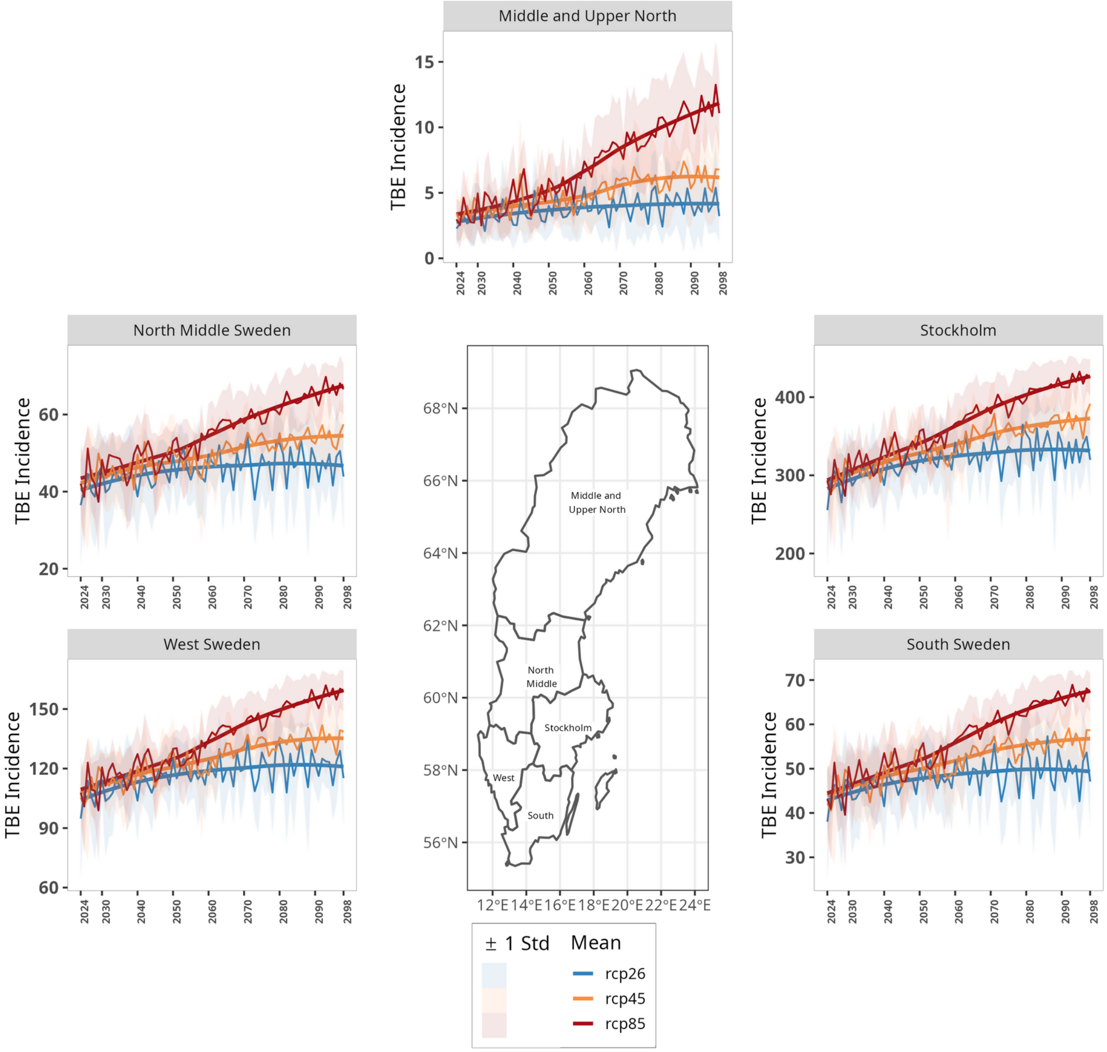

Figure S19: Projected trends in TBE incidence by RCP and regions in Sweden for the period 2024-2098.The shared regions represent uncertainty (± 1) standard deviations computed from the contributing regional climate circulation models.

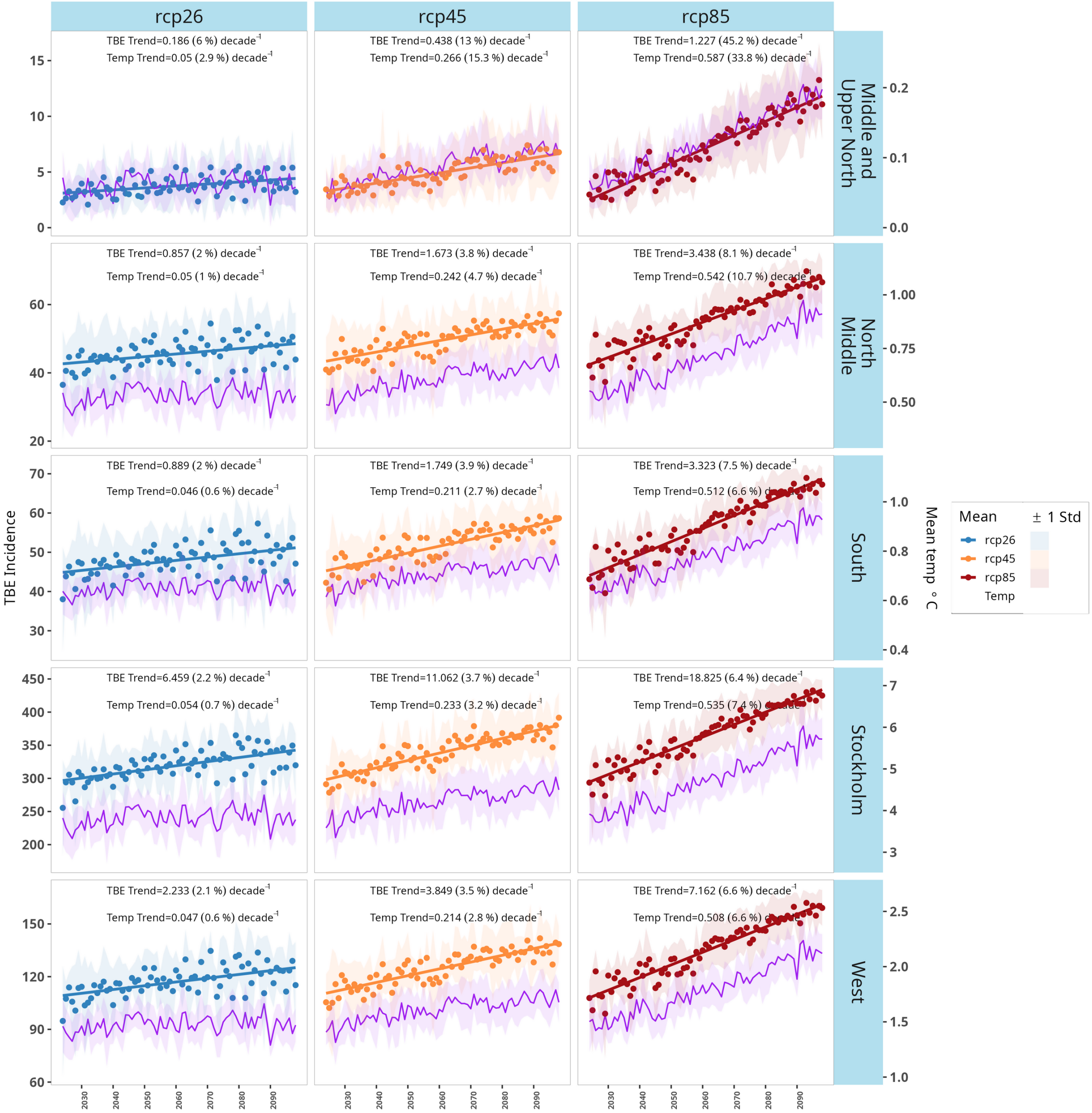

Figure S20: Estimated decadal linear trends in projected TBE incidence rate and mean temperature for RCP26, RCP45 and RCP85 for the period 2024-2098 by regions in Sweden. The shaded regions represent uncertainty (± 1) standard deviations computed from the contributing regional climate circulation models.

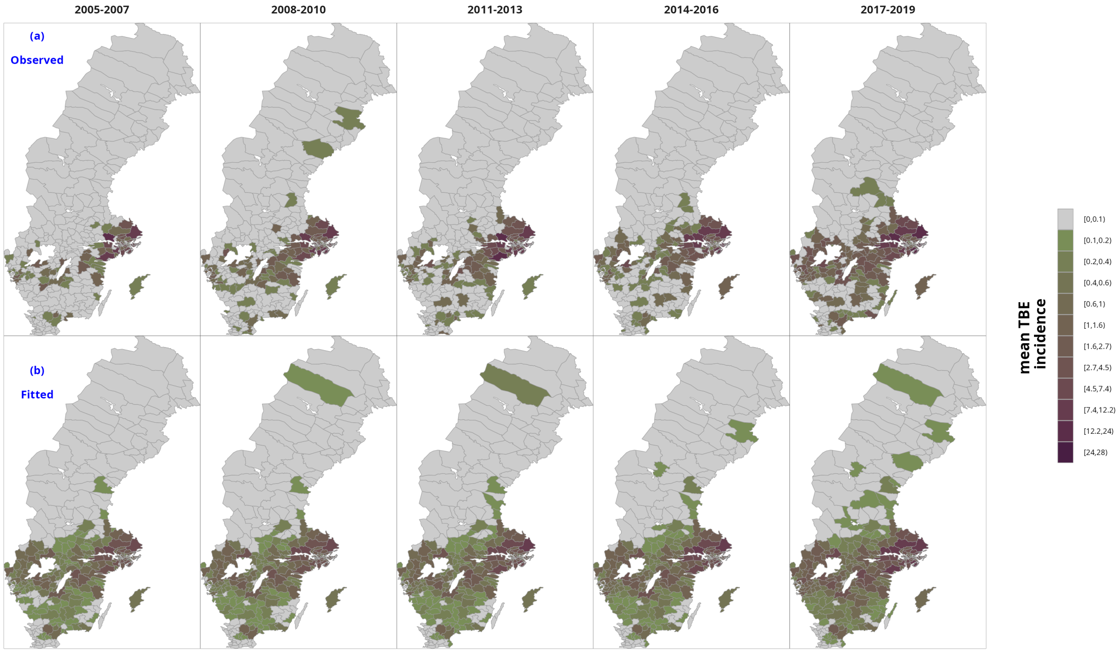

Figure S21: Observed (a) vs Fitted (b) mean annual TBE incidence in Sweden for period, 2005-2019 by period.

References

1. Cornes, R. C., van der Schrier, G., van den Besselaar, E. J. M. & Jones, P. D. An Ensemble Version of the E-OBS Temperature and Precipitation Data Sets. *Journal of Geophysical Research: Atmospheres* **123,** 9391–9409. ISSN: 2169-897X. https://agupubs.onlinelibrary.wiley.com/doi/abs/10.1029/2017JD028200 (2018).
2. *European Commission Directorate-General Joint Research Centre. Normalised Difference Vegetation Index 1999-2020 (raster 1 km), global, 10-daily - version 3* Dataset. https://globalland.vito.be/geonetwork/srv/api/records/clms_global_ndvi_1km_v3_10daily.
3. *European Commission Directorate-General Joint Research Centre. Normalised Difference Vegetation Index 2020-present (raster 300 m), global, 10-daily - version 2* Dataset. https://globalland.vito.be/geonetwork/srv/api/records/clms_global_ndvi_300m_v2_10daily.
4. *European Commission Directorate-General Joint Research Centre. Leaf Area Index 1999-2020 (raster 1 km), global, 10-daily - version 2* Dataset. https://globalland.vito.be/geonetwork/srv/api/records/clms_global_lai_1km_v2_10daily.
5. *European Commission Directorate-General Joint Research Centre. Leaf Area Index 2014-present (raster 300 m), global, 10-daily - version 1* Dataset. https://globalland.vito.be/geonetwork/srv/api/records/clms_global_lai_300m_v1_10daily.
6. *European Commission Directorate-General Joint Research Centre. Fraction of Vegetation Cover 1999-2020 (raster 1 km), global, 10-daily - version 2* Dataset. https://globalland.vito.be/geonetwork/srv/api/records/clms_global_fcover_1km_v2_10daily.
7. *European Commission Directorate-General Joint Research Centre. Fraction of Vegetation Cover 2014-present (raster 300 m), global, 10-daily - version 1* Dataset. https://globalland.vito.be/geonetwork/srv/api/records/clms_global_fcover_300m_v1_10daily.
8. Achazi, K. *et al.* Rodents as sentinels for the prevalence of tick-borne encephalitis virus. *Vector Borne Zoonotic Dis* **11,** 641–7. ISSN: 1530-3667 (Print) 1530-3667 (2011).
9. Institute, S. N. V. *Rapportera vilda djur* Web Page. 2024. https://rapporteravilt.sva.se/.
10. *Conservation status of habitat types and species: datasets from Article 17, Habitats Directive 92/43/EEC reporting (2013-2018) - PUBLIC VERSION - Aug. 2020* Dataset. https://www.eea.europa.eu/en/datahub/datahubitem-view/d8b47719-9213-485a-845b-db1bfe93598d?activeAccordion=1082742.

11. Cervellini, M. *et al.* Diversity of European habitat types is correlated with geography more than climate and human pressure. *Ecology and evolution* **11,** 18111–18124. ISSN: 2045-7758 (2021).

12. Rue, H., Martino, S. & Chopin, N. Approximate Bayesian inference for latent Gaussian models by using integrated nested Laplace approximations. *Journal of the Royal Statistical Society: Series B (Statistical Methodology)* **71,** 319–392. ISSN: 1369-7412. https://rss.onlinelibrary.wiley.com/doi/abs/10.1111/j.1467-9868.2008.00700.x (2009).

13. Lindgren, F., Rue, H. & Lindström, J. An explicit link between Gaussian fields and Gaussian Markov random fields: the stochastic partial differential equation approach. *Journal of the Royal Statistical Society: Series B (Statistical Methodology)* **73,** 423–498. ISSN: 1369-7412. https://rss.onlinelibrary.wiley.com/doi/abs/10.1111/j.1467-9868.2011.00777.x%20https://rss.onlinelibrary.wiley.com/doi/pdfdirect/10.1111/j.1467-9868.2011.00777.x?download=true (2011).

14. Lindgren, F. & Rue, H. Bayesian Spatial Modelling with R-INLA. *2015* **63,** 25. ISSN: 1548-7660. https://www.jstatsoft.org/v063/i19 (2015).

15. R Core Team. *R: A Language and Environment for Statistical Computing* R Foundation for Statistical Computing (Vienna, Austria, 2025). https://www.R-project.org/.

16. Simpson, D., Illian, J. B., Lindgren, F., Sørbye, S. H. & Rue, H. Going off grid: computationally efficient inference for log-Gaussian Cox processes. *Biometrika* **103,** 49–70. ISSN: 0006-3444. https://doi.org/10.1093/biomet/asv064%20https://watermark.silverchair.com/asv064.pdf? (2016).

17. Righetto, A. J., Faes, C., Vandendijck, Y. & Ribeiro, P. J. On the choice of the mesh for the analysis of geostatistical data using R-INLA. *Communications in Statistics - Theory and Methods* **49,** 203–220. ISSN: 0361-0926. https://doi.org/10.1080/03610926.2018.1536209%20https://www.tandfonline.com/doi/full/10.1080/03610926.2018.1536209 (2020).

18. Naimi, B., Hamm, N. A. S., Groen, T. A., Skidmore, A. K. & Toxopeus, A. G. Where is positional uncertainty a problem for species distribution modelling? *Ecography* **37,** 191–203. ISSN: 0906-7590. https://nsojournals.onlinelibrary.wiley.com/doi/abs/10.1111/j.1600-0587.2013.00205.x (2014).

19. Fuglstad, G.-A., Simpson, D., Lindgren, F. & Rue, H. Constructing priors that penalize the complexity of Gaussian random fields. *Journal of the American Statistical Association* **114,** 445–452. ISSN: 0162-1459 (2019).

20. Stefanoff, P. *et al.* A Predictive Model Has Identified Tick-Borne Encephalitis High-Risk Areas in Regions Where No Cases Were Reported Previously, Poland, 1999-2012. *Int J Environ Res Public Health* **15.** ISSN: 1661-7827 (Print) 1660-4601 (2018).

21. Hönig, V. *et al.* Model of risk of exposure to Lyme borreliosis and tick-borne encephalitis virus-infected ticks in the border area of the Czech Republic (South Bohemia) and Germany (Lower Bavaria and Upper Palatinate). *International journal of environmental research and public health* **16,** 1173. ISSN: 1660-4601. https://pmc.ncbi.nlm.nih.gov/articles/PMC6479554/ (2019).

22. Kjær, L. J. *et al.* Predicting and mapping human risk of exposure to Ixodes ricinus nymphs using climatic and environmental data, Denmark, Norway and Sweden, 2016. *Eurosurveillance* **24,** 1800101. https://pmc.ncbi.nlm.nih.gov/articles/PMC6402176/ (2019).

23. Brugger, K. *et al.* A density map of the tick-borne encephalitis and Lyme borreliosis vector Ixodes ricinus (Acari: Ixodidae) for Germany. *Journal of medical entomology* **53,** 1292–1302. ISSN: 1938-2928 (2016).

24. Dagostin, F. *et al.* Ecological and environmental factors affecting the risk of tick-borne encephalitis in Europe, 2017 to 2021. *Eurosurveillance* **28,** 2300121. https://pmc.ncbi.nlm.nih.gov/articles/PMC10588310/ (2023).

25. DOMŞA, C., Mihalca, A. D. & Sandor, A. D. Modeling the distribution of Ixodes ricinus in Romania. *North-Western Journal of Zoology* **14.** ISSN: 1584-9074 (2018).

26. Lindgren, E. & Gustafson, R. Tick-borne encephalitis in Sweden and climate change. *Lancet* **358,** 16–8.

27. Rosà, R. *et al.* Effect of climate and land use on the spatio-temporal variability of tick-borne bacteria in Europe. *International journal of environmental research and public health* **15,** 732. ISSN: 1660-4601. https://pmc.ncbi.nlm.nih.gov/articles/PMC5923774/ (2018).

28. Knap, N. & Avšič-Županc, T. Factors affecting the ecology of tick-borne encephalitis in Slovenia. *Epidemiology and Infection* **143,** 2059–2067. ISSN: 0950-2688 (2015).

29. Shchuchinova, L. D., Kozlova, I. V. & Zlobin, V. I. Influence of altitude on tick-borne encephalitis infection risk in the natural foci of the Altai Republic, Southern Siberia. *Ticks and Tick-borne Diseases* **6,** 322–329. ISSN: 1877-959X. https://www.sciencedirect.com/science/article/pii/S1877959X1500031X%20https://www.sciencedirect.com/science/article/abs/pii/S1877959X1500031X?via%3Dihub (2015).

30. Hönig, V. *et al.* Ticks and tick-borne pathogens in South Bohemia (Czech Republic)–Spatial variability in Ixodes ricinus abundance, Borrelia burgdorferi and tick-borne encephalitis virus prevalence. *Ticks and tick-borne diseases* **6,** 559–567. ISSN: 1877-959X. https://www.sciencedirect.com/science/article/abs/pii/S1877959X15000783?via%3Dihub (2015).

31. Kiffner, C. *et al.* Determinants of tick-borne encephalitis in counties of southern Germany, 2001-2008. *International Journal of Health Geographics* **9,** 42. ISSN: 1476-072X (2010).

32. Uusitalo, R. *et al.* Modelling habitat suitability for occurrence of human tick-borne encephalitis (TBE) cases in Finland. *Ticks and Tick-borne Diseases* **11,** 101457. ISSN: 1877-959X. `https://www.sciencedirect.com/science/article/pii/S1877959X19305114?via%3Dihub` (2020).

33. Dagostin, F. *et al.* High habitat richness reduces the risk of tick-borne encephalitis in Europe: A multi-scale study. *One Health* **18,** 100669. ISSN: 2352-7714. `https://www.sciencedirect.com/science/article/pii/S2352771423001891` (2024).

34. CARPI, G., CAGNACCI, F., NETELER, M. & RIZZOLI, A. Tick infestation on roe deer in relation to geographic and remotely sensed climatic variables in a tick-borne encephalitis endemic area. *Epidemiology and Infection* **136,** 1416–1424. ISSN: 1469-4409 (2008).

35. Jaenson, T. G. T. & Lindgren, E. The range of Ixodes ricinus and the risk of contracting Lyme borreliosis will increase northwards when the vegetation period becomes longer. *Ticks and Tick-borne Diseases* **2,** 44–49. ISSN: 1877-959X. `https://www.sciencedirect.com/science/article/pii/S1877959X10000919` (2011).

36. Jaenson, T. G. T. *et al.* The importance of wildlife in the ecology and epidemiology of the TBE virus in Sweden: incidence of human TBE correlates with abundance of deer and hares. *Parasites and Vectors* **11,** 477.

37. Zeimes, C. B., Olsson, G. E., Hjertqvist, M. & Vanwambeke, S. O. Shaping zoonosis risk: landscape ecology vs. landscape attractiveness for people, the case of tick-borne encephalitis in Sweden. *Parasites and vectors* **7,** 370. ISSN: 1756-3305 (2014).
